# Metal-Free Ferromagnetism in Triangulene Two-Dimensional Frameworks

**DOI:** 10.1021/jacs.5c21206

**Published:** 2026-03-30

**Authors:** Hongde Yu, Thomas Heine

**Affiliations:** † Faculty of Chemistry and Food Chemistry, 9169 Technische Universität Dresden, Bergstraße 66c, 01062 Dresden, Germany; ‡ Institute of Resource Ecology, Helmholtz Zentrum Dresden-Rossendorf, Permoserstraße 15, 04318 Leipzig, Germany; § Department of Chemistry, Yonsei University, Seodaemun-gu, Seoul 120-749, Republic of Korea

## Abstract

Achieving room-temperature ferromagnetism in purely organic two-dimensional (2D) materials remains a fundamental challenge. Here, we introduce a mixed-topology strategy to induce strong ferromagnetic (FM) coupling in metal-free 2D frameworks. By covalently connecting non-Kekulé polycyclic aromatic hydrocarbon (PAH) radicals with distinct sublattice topologies, we rationally break inversion and time-reversal symmetries and thereby unlock intrinsic FM order in π-conjugated 2D networks. Leveraging this concept, we computationally designed a family of 32 FM 2D frameworks featuring spin-1/2 and hybrid spin-1/2–spin-1 honeycomb lattices. First-principles calculations revealed that these organic 2D frameworks are robust FM semiconductors with tunable spin-dependent bandgaps (0.9–3.8 eV). Remarkably, they exhibit record-high FM couplings of up to 127 meV, spin-splitting energies exceeding 2 eV, and crossover (Curie) temperatures above 550 K, ensuring ferromagnetic order stability far beyond room temperature. The strong FM interaction originates from substantial overlap between half-filled, delocalized π-orbitals, which enhances direct magnetic exchange while suppressing antiferromagnetic superexchange. These findings establish an effective design principle for metal-free FM semiconductors and pave the way for flexible magnets for next-generation spintronics and quantum technologies.

## Graphical abstract

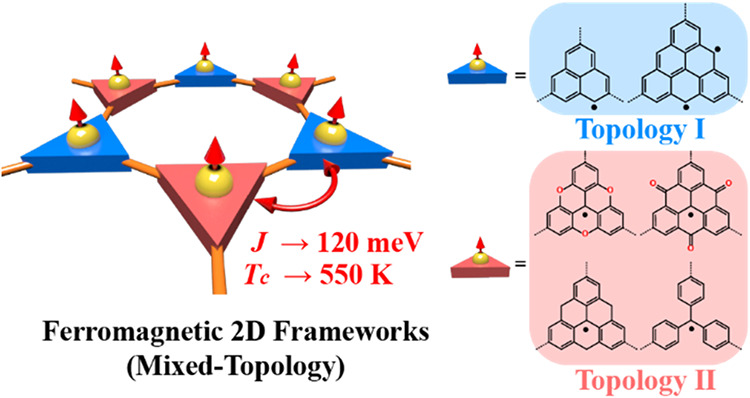

## Introduction

Metal-free magnetic materials are garnering growing attention as promising candidates for next-generation spintronic and quantum devices, owing to their lightweight character, environmental sustainability, and outstanding chemical tunability.^1–5^ Unlike conventional inorganic magnets, where magnetism originates from localized d- or f-orbitals of metal atoms, metal-free magnetism emerges from strong electron correlation within partially filled, delocalized π-orbitals.^2,6^ This distinctive π-electron magnetism has been observed in diverse platforms, including defected graphene,^7,8^ magic-angle twisted bilayer graphene,^9,10^ graphene nanoribbons,^3,11,12^ and low-dimensional polymers,^2,5,13,14^ which exhibit exotic quantum phases such as fractional quantum Hall effect,^15,16^ unconventional superconductivity,^17–19^ and correlated insulating states.^2,17,20^ Among these, conjugated two-dimensional (2D) frameworks have emerged as versatile platforms for engineering correlated π-magnetism.^1,6^ They are constructed by covalently linking molecular building blocks into precisely defined topologies including monolayer 2D polymers and layer-stacked covalent organic frameworks (COFs). Most 2D frameworks, however, are made of closed-shell molecules and are thus intrinsically diamagnetic.^1,21^ Incorporating open-shell units can preserve the spin polarization of the monomers but typically yields antiferromagnetic (AFM) ordering,^2,4,5^ due to strong electronic coupling and dominant superexchange interactions that favor antiparallel spin alignment.^1,22–24^ However, metal-free ferromagnetic (FM) materials with macroscopic magnetization and robust spin splitting are highly desirable for external manipulation of spin degrees of freedom.^3^ Realizing room-temperature ferromagnetism in purely organic systems would represent a major breakthrough,^3,9,10^ enabling a new generation of flexible magnetic materials. Despite intensive efforts, achieving room-temperature FM in organic 2D frameworks remains a formidable challenge.^25–28^

Overcoming this challenge requires not only the development of π-conjugated radicals as spin units, but also effective strategies to control magnetic interactions across extended π-frameworks.^5,6,29^ Recent advances have enabled various low-dimensional magnetic architectures, particularly those based on non-Kekulé polycyclic aromatic hydrocarbon (PAH) radicals, such as triangulenes^30,31^ and Clar’s goblets.^13,32^ These systems have facilitated the exploration of correlated spin phenomena, including spinon excitations and Haldane phases with fractional edge states in one-dimensional (1D) spin chains, and Mott–Hubbard insulating behavior in magnetic 2D networks.^2,4,5,33–37^ However, in such π-conjugated systems, through-bond magnetic interactions between spin centers remain predominantly AFM.^2,4,5,33^

Significant efforts have been devoted to inducing ferromagnetism in extended π-conjugated systems.^3,25,26,29,38–40^ For instance, Janus graphene nanoribbons with asymmetric edges have been shown to host FM ground states.^3^ Lieb’s theorem^41^ (i.e., Ovchinnikov rule^42^) establishes that introducing sublattice imbalance yields a nonzero total spin quantum number *S =* (*N*_A_ *– N*_B_)/2, where *N*_A_ and *N*_B_ are the numbers of sites for the A and B sublattices^43,44^ (Figure 1). Achieving FM coupling above 10 meV, however, remains significantly challenging.^6,29,38–40^ State-of-the-art systems typically exhibit FM couplings of only ∼1 meV in oligomers employing *meta*-phenylene linkers^38,39^ and ∼7 meV in 1D spin chains based on dibenzotriangulene,^29,40^ primarily due to the inefficient communication between spin orbitals. This persistent limitation highlights the requirement for an efficient molecular design principle capable of enabling strong through-bond FM coupling and, ultimately, supporting room-temperature, metal-free 2D ferromagnets.

**Figure 1:**
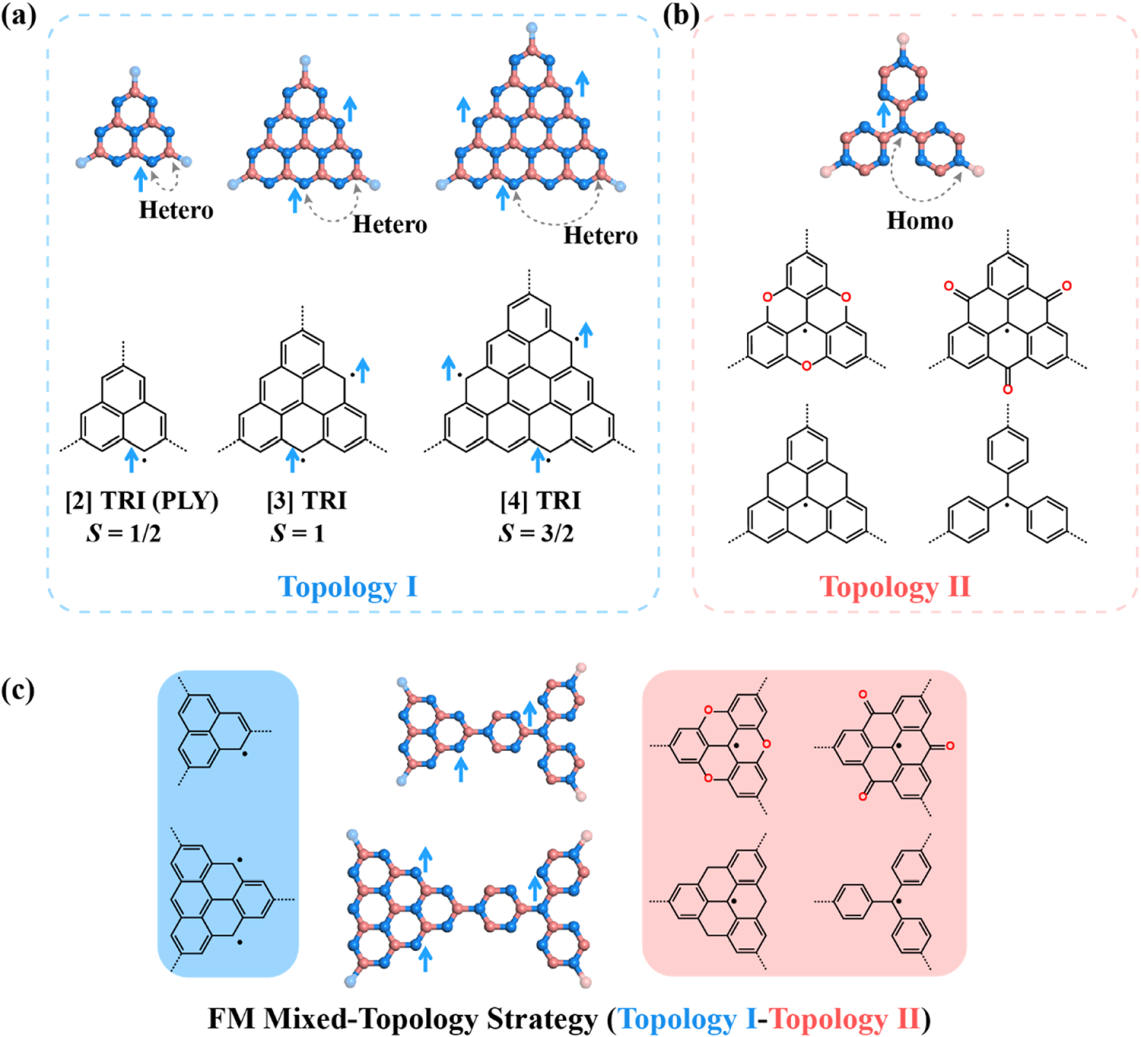
Mixed-topology strategy for achieving metal-free ferromagnetism (FM) in organic 2D frameworks with a honeycomb lattice. Classification of radical monomers into (a) Topology I and (b) Topology II, according to sublattice topologies of the connecting and the spin-bearing sites. (a) Topology I monomers feature majority spins on one sublattice and connecting (corner) sites on the opposite sublattice (heterospin configuration). (b) Topology II monomers exhibit both majority spins and connecting sites on the same sublattice (homospin configuration). (c) Schematic representation of the mixed-topology strategy, wherein Topology I and Topology II monomers are covalently linked to form binary 2D frameworks with parallel spin alignment. Color coding: blue and red denote sublattices A and B, respectively. Connecting atoms of the nearest neighbors are marked in light blue or red, respectively.

Triangulene, the smallest triplet-ground-state nanographene, serves as a prototypical multiradical building unit for exploring metal-free magnetism in 2D honeycomb frameworks^45^ (Figure 1). Here, we introduce a mixed-topology design strategy that enables strong FM coupling and room-temperature FM in organic 2D frameworks. This approach utilizes radical monomers with distinct sublattice topologies—denoted as Topology I (hetero) and Topology II (homo)—to construct binary triangulene-based networks, where the topologies are defined by the sublattices of the connecting sites relative to the spin-bearing sites (Figure 1). In addition to selectively aligning majority spins, this strategy promotes efficient spin–orbital overlap between delocalized, half-filled π-orbitals, thereby yielding strong FM couplings across the lattice. Using this design principle, we construct 32 representative 2D frameworks and demonstrate via first-principles calculations that all of them are FM semiconductors. These systems show unprecedented FM couplings of up to 127 meV and crossover (Curie) temperatures well above room temperature, reaching 556 K. From a molecular perspective, this pronounced FM coupling originates from strong direct exchange between adjacent spin centers. Owing to broken inversion symmetry and the consequent lifting of Kramers spin degeneracy, these FM 2D lattices exhibit large spin-splitting energies (1–2 eV), together with tunable spin-dependent bandgaps (0.9–3.8 eV). This work establishes an effective blueprint for metal-free, chemically tunable organic magnets and opens new opportunities for flexible spintronic and quantum devices.

## Results and Discussion

### Mixed-Topology Strategy for Ferromagnetic 2D Frameworks

As illustrated in Figure 1a, triangulene (TRI)-based radical monomers can be classified into two distinct sublattice topologies (i.e., Topology I (hetero) and Topology II (homo)), according to the sublattice identities of their majority-spin sites and the corner sites (connecting sites). The corner sites are typically brominated to enable polymerization into extended structures such as 1D spin chains and 2D lattices,^2,4,6^ whereas edge sites are functionalized^6,46^ (Figures 1 and S1). In Topology I, the majority-spin sites and the corner sites reside on opposite sublattices (i.e., hetero) of the underlying bipartite lattice (Figure 1a). In contrast, Topology II monomers feature both the dominant spin and the corner sites located on the same sublattice (i.e., homo) (Figure 1b). A variety of triangular nanographenes with zigzag edges fall into Topology I, including phenalenyl (PLY or [2]­TRI),^47,48^ [3]­TRI, [4]­TRI,^49^ and [5]­TRI.^50^ For these systems, both the total spin quantum number (*S*) and the number of energy-degenerate singly occupied molecular orbitals (SOMOs) (i.e., zero-mode numbers) increase linearly with edge extension, in agreement with Lieb’s theorem^6,31,43,44,51^ (Figure 1a). In comparison, many heterotriangulenes exemplify Topology II, such as trioxatriangulene (TAM),^52^ trioxotriangulene (TOT),^53^ trihydrotriangulene (TRIH),^54^ and triphenylmethyl (TPM)^55^ (Figure 1b). These radicals have been synthesized via both in-solution and on-surface approaches.^47,48^ They generally exhibit *D*_3*h*_ symmetry with delocalized spin density across a planar, fully π-conjugated carbon skeleton (Figure S2). TPM represents an exception: steric hindrance between adjacent hydrogen atoms on the peripheral benzene rings forces the molecule into a nonplanar geometry with *C*_3_ symmetry^56^ (Figures 1a and S2).

By covalently linking TRI-based radical units through their corner sites, diverse π-conjugated magnetic architectures can be constructed, including spin dimers,^57,58^ quantum rings,^59^ 1D spin chains, and 2D frameworks.^2,6,33^ We note that, when monomers with distinct sublattice topologies—specifically a Topology I unit connected to a Topology II unit—are covalently joined (Topology I–Topology II), the unpaired spins on adjacent units preferentially align in parallel, as per Lieb’s theorem (Figure 1c). This will result in high-spin ground states with *S* = *S*_1_ + *S*_2_, where the *S*_1_ and *S*_2_ are the spin multiplicities of the constituent monomers (Figure 1c and Table 1). This behavior contrasts sharply with the widely studied systems composed of spin units sharing the same sublattice topology (i.e., Topology I–Topology I or Topology II–Topology II), which generally display AFM or ferrimagnetic interactions and consequently low-spin ground states of *S* = |*S*_1_ – *S*_2_| ^2,6,26,33,34,59,60^ (Figures S3–S5). These results indicate that our mixed-topology design strategy enables parallel spin alignments and through-bond FM coupling, providing a route toward achieving intrinsic metal-free ferromagnetism in 2D π-conjugated frameworks. Building on this concept, we designed 32 FM 2D frameworks assembled from stable (hetero)­triangulene radical monomers (Figure 2a). These spin units are connected either directly or through π-conjugated linear linkers, including ethynylene (–C ≡ C–, CC), butadiyne (–C≡C–C≡C–, CCCC), and *para*-phenylene (Ph) groups (Figures 2a and S6–S13), facilitating delocalized spin density throughout the lattice (Figure 3).

**Figure 2:**
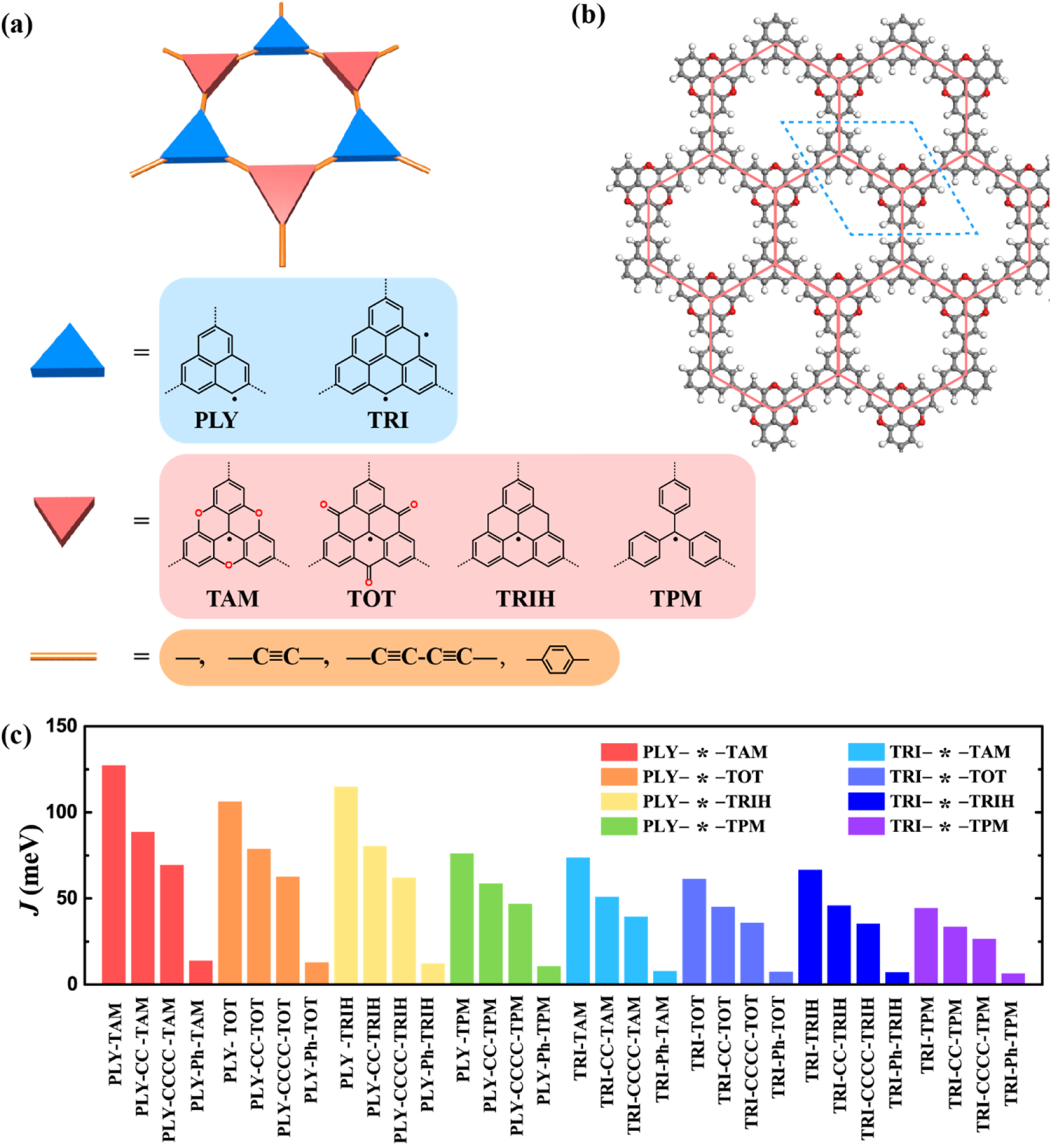
Structures and magnetic couplings of FM 2D frameworks. (a) Structure illustration of FM binary 2D frameworks, connecting Topology I monomers (PLY and TRI) with Topology II monomers (TAM, TOT, TRIH, and TPM) via conjugated linkers—acetylene, diacetylene, or *para*-phenylene—to construct extended 2D frameworks. (b) Atomic structure of a representative binary 2D framework, [PLY-TAM], showing the periodic arrangement of Topology I and Topology II units in a honeycomb lattice geometry. (c) Predicted magnetic couplings (*J*) for 32 FM 2D frameworks, calculated using density functional theory (PBE0 level).

**Figure 3:**
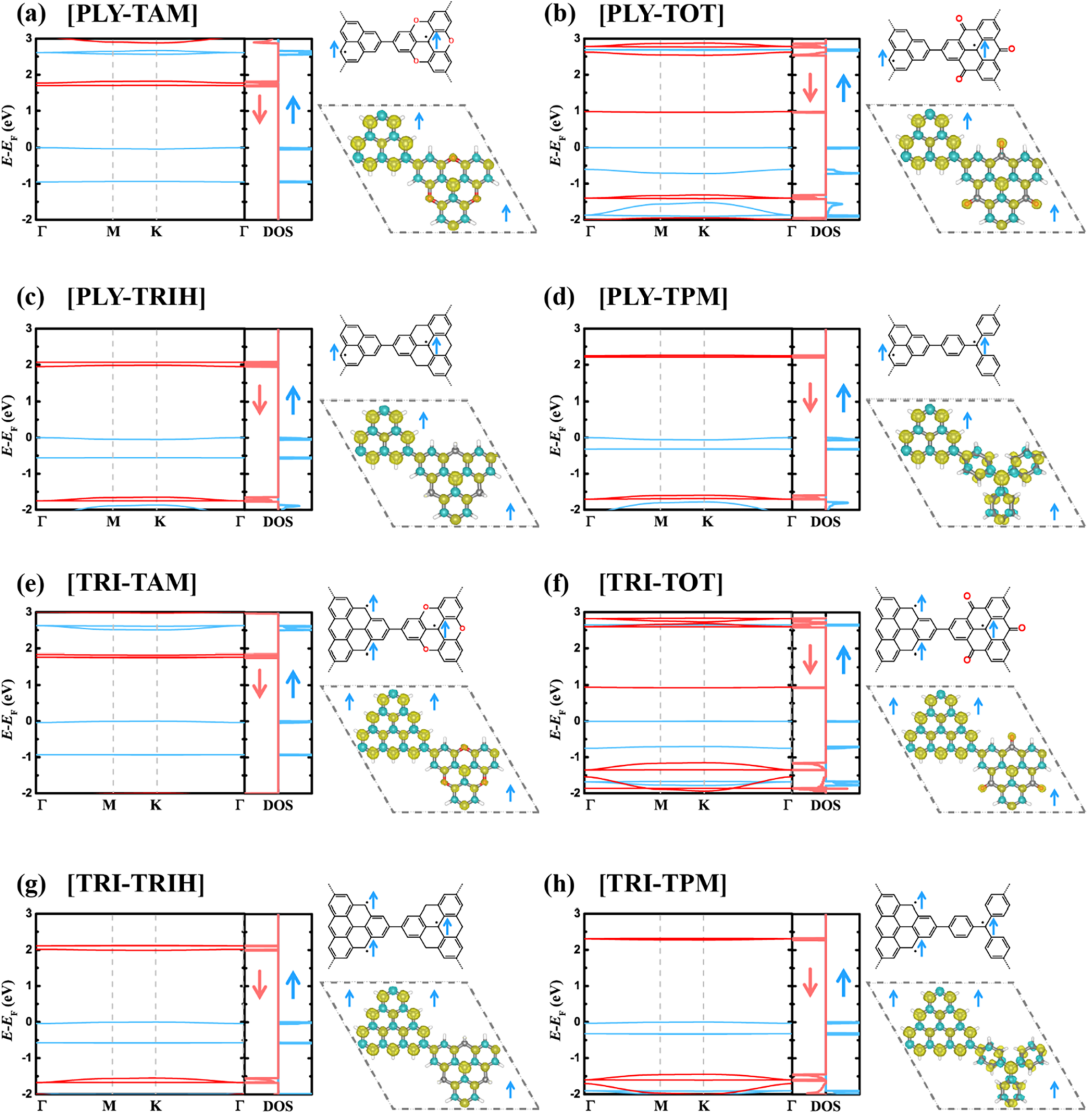
Spin-polarized electronic structures of FM 2D frameworks. (a–h) Spin-polarized band structures, densities of states (DOS), molecular structures, and spin density distributions for [PLY-TAM], [PLY-TOT], [PLY-TRIH], [PLY-TPM], [TRI-TAM], [TRI-TOT], [TRI-TRIH], and [TRI-TPM], respectively. Blue and red lines denote spin-up and spin-down channels, respectively.

**Table 1:** Magnetic and Electronic Properties of Representative FM 2D Frameworks, Where the Monomers Are Coupled Directly^a^

	*J* (meV)	*S*	*M* (μ_B_)	*E*_split_ (eV)	*E*_g_^↑↑^ (eV)	*E*_g_^↓↓^ (eV)	*E*_g_^↑↓^ (eV)	*T*_c_ (K)	*S*_o_	Δϵ (eV)	Δ*E*_S–L_ (eV)
[PLY-TAM]	127	1/2, 1/2	2	2.11	2.56	3.81	1.70	556	0.23	0.76	1.56
[PLY-TOT]	106	1/2, 1/2	2	1.31	2.67	2.27	0.96	470	0.27	1.36	0.59
[PLY-TRIH]	115	1/2, 1/2	2	1.64	3.19	3.63	1.98	506	0.21	0.48	1.85
[PLY-TPM]	76	1/2, 1/2	2	1.60	3.56	3.81	2.21	336	0.18	0.07	2.26
[TRI-TAM]	74	1, 1/2	3	1.99	2.51	3.75	1.75	570	0.18	0.88	1.58
[TRI-TOT]	61	1, 1/2	3	1.15	2.64	2.08	0.93	470	0.24	1.25	0.69
[TRI-TRIH]	66	1, 1/2	3	1.55	3.09	3.54	1.99	508	0.18	0.58	1.88
[TRI-TPM]	44	1, 1/2	3	1.45	3.45	3.72	2.27	338	0.21	0.17	2.29

a The magnetic couplings (*J*), spin quantum numbers (*S*) of spin units, total magnetic moments (*M*) per unit cell, spin-splitting energies (*E*_split_), spin-polarized bandgaps for spin-up and spin-down channels (*E*_g_^↑↑^ and *E*_g_^↓↓^), spin-flip gaps (*E*_g_^↑↓^), and crossover (Curie) temperatures (*T*_c_) are shown. To provide molecular insights, spin overlap integrals (*S*_o_), chemical potential (on-site energy) offsets (Δϵ), and SOMO-to-LUMO energy gaps (Δ*E*_S–L_) of the building units are included. All results are calculated at the PBE0 level. The *J* values are calculated using eq 2. See the Computational Methods section in the Supporting Information for details.

### Magnetic Interactions in Ferromagnetic 2D Frameworks

All 32 2D frameworks adopt a honeycomb lattice, reminiscent of graphene and hexagonal boron nitride (h-BN) (Figures 2b and S14). This honeycomb lattice is energetically more favorable than other 2D (e.g., the square lattice) or 3D isomers^22,60–63^ (Figure S15). Because of the difference in monomer sizes, an alternating arrangement in the binary 2D lattice is energetically favored, as it minimizes lattice distortion and strain compared to nonuniform (or random) distributions^60^ (Figure S16). *Ab initio* molecular dynamics simulations demonstrate the robust thermal stability of [PLY-TAM], with the 2D in-plane structure remaining intact after 10 ps of dynamics in the NVT ensemble up to 500 K (Figure S17) (see computational details in the Supporting Information). This stability arises primarily from the strong, covalently conjugated C–C bonds within the structure.^2^ The half-filled, degenerate π-orbitals located on each spin unit introduce strong electron–electron correlations, driving spontaneous spin polarization. To validate our mixed-topology design principle, we systematically investigated the magnetic properties of these 2D lattices using first-principles calculations, specifically spin-polarized density functional theory (see Methods for computational details). For every system studied, the FM state is energetically more favorable than both the AFM (or ferrimagnetic) and diamagnetic states, in agreement with our design concept (Table 1 and Figure 3). In the context of magnetic interaction, the Heisenberg–Dirac–van Vleck (HDVV) Hamiltonian is defined as

1 $$Ĥ=-\Sigma_{\left\langle i,j\right\rangle }J_{\mathrm{ij}}{Ŝ}_{i}{Ŝ}_{j}$$

where *J*_*ij*_ is the magnetic coupling that describes the strength and nature of the interaction between spin units *i* and *j*. A positive *J* value corresponds to FM coupling, whereas a negative *J* value corresponds to AFM coupling. The *J* values were obtained by mapping the energy difference between the AFM and FM states onto the HDVV Hamiltonian. For a system with *z* neighboring spin pairs and local spins *S*_1_ and *S*_2_ within a unit cell, the magnetic coupling is calculated by^64^

2 $$J=\left(E_{\mathrm{AFM}}-E_{\mathrm{FM}}\right)/\left(2{\mathrm{zS}}_{1}S_{2}\right)$$

As shown in Figures S18 and S19, the magnitude of spin density remains comparable between the FM and AFM states. This negligible difference validates the use of the Heisenberg Hamiltonian, consistent with its widespread application in prior studies of open-shell polycyclic aromatic hydrocarbon systems.^2,4,5,25,34,60^ The resulting *J* values span a wide range, from 44 to 127 meV for directly linked 2D frameworks (Table 1 and Figure 2c). Notably, [PLY-TAM] exhibits an unprecedented FM coupling of 127 meV, representing the record-high through-bond FM interaction in purely organic 2D systems to date^25,26,28^ (Table 1). Other PLY-based systems, including [PLY-TOT], [PLY-TRIH], and [PLY-TPM], also show exceptionally strong FM couplings above 70 meV. These *J* values substantially exceed those observed in previously reported 2D magnetic materials, such as Cr_2_Ge_2_Te_6_ and CrI_3_, with *J* values on the order of 4 meV.^3,29,38–40,65–67^ Although a direct numerical comparison of FM coupling strength is nontrivial because of the different local spin quantum numbers (*S* = 1/2 or mixed *S* = 1/2 and 1 in these 2D frameworks versus *S* = 3/2 in CrI_3_), the FM coupling in our 2D frameworks exhibit a markedly larger energy scale, consistent with the enhanced *J* value compared to conventional d-electron-based 2D inorganic ferromagnets (see Supporting Note in the Supporting Information). Although the nearest-neighbor FM coupling is substantial, the next-nearest-neighbor interaction is negligible (at the sub-meV level), resulting in |*J*_1_|/|*J*_2_| ratios ranging from approximately 100 to 1000 (Table S1). In contrast to the uniform spin-1/2 networks characteristic of PLY-based frameworks, TRI-based lattices form hybrid spin architectures composed of alternating spin-1 (TRI) and spin-1/2 (Topology II) units (Table 1). We further find that the magnitude of *J* decreases systematically with increasing linker length from direct bonding to −CC–, −CCCC–, and −Ph–, due to the reduced spatial overlap between spin orbitals (Table 1, Figures 2c and S20–S43). This trend suggests that linkers containing an odd number of carbon atoms, such as *meta*-phenylene or −CCC–, should likewise produce weak magnetic interactions, consistent with the ∼1 meV coupling observed in *meta*-phenylene-linked triangulene dimers. The stability of different spin states in acetylene-bridged phenalenyl systems with different connection topologies has been investigated.^68^

Although Lieb’s theorem, derived from the standard Hubbard model for half-filled bipartite lattices, provides qualitative insight into the emergence of net magnetization,^41^ it does not offer a quantitative description of FM coupling primarily because the intersite direct magnetic exchange is not considered. To address this limitation, we introduce an extended Hubbard Hamiltonian that incorporates both direct exchange and chemical potential:

H^=−t∑⟨i,j⟩,σ(ci,σ†cj,σ+cj,σ†ci,σ)+U∑ini↑ni↓+∑iϵini+K∑⟨i,j⟩∑σ,σ′ci,σ†cj,σ′†ci,σ′cj,σ 3

Here, *t* and *U* denote the intersite hopping integral (i.e., electronic coupling) and on-site Coulomb repulsion, respectively, as in the conventional Hubbard model. The additional terms ϵ and *K* represent the on-site energy (chemical potential) and the direct magnetic exchange, respectively. The ϵ term, as in the tight-binding model, is crucial for describing binary lattices such as h-BN and the mixed-topology 2D frameworks studied here. The interplay between chemical potential offset Δϵ and *U* determines the accessible quantum phases: a band-insulating state emerges when Δϵ ≫ *U*, as in h-BN, whereas a Mott–Hubbard insulating phase is favored when *U* ≫ Δϵ, as in homogeneous AFM 2D frameworks (Δϵ = 0), such as [TRI] and [PLY],^6,25–27^ as well as binary 2D systems studied here (Table 1). For example, Δϵ is 0.76 eV in [PLY-TAM], due to the substantial SOMO energy mismatch between adjacent spin units, which remains considerably smaller than the on-site repulsion *U* = 2.66 eV for the PLY unit.^26^ Moreover, the combination of a large Δϵ and sublattice imbalance strongly suppresses the hopping integral *t*. In [PLY-TAM], *t* is reduced to ∼1 meV (Figure S44), in stark contrast to homogeneous AFM systems with Δϵ = 0, such as TAM–TAM (*t* = 0.62 eV) and PLY–PLY (*t* = 0.39 eV).^69^

According to the Goodenough–Kanamori rules^70^ and this extended model Hamiltonian, the overall magnetic coupling *J* arises from the competition between the FM direct exchange *K* and the AFM kinetic superexchange −4*t* ^2^/*U*. As *t* is extremely small in these binary 2D frameworks, the AFM superexchange term becomes negligible. Instead, FM direct exchange, originating from the direct spatial overlap between nearest spin orbitals, dominates the magnetic interactions (Table 1). The magnitude of this direct overlap is reflected in the SOMO orbital overlap integrals (*S*_o_), which are substantial in these systems, such as [PLY-TAM] (*S*_o_ = 0.23) and [PLY-TOT] (*S*_o_ = 0.27) (Table 1). This pronounced spin–orbital overlap and the resulting strong FM direct exchange are rooted in the highly delocalized π-orbitals in these conjugated 2D frameworks. This behavior contrasts sharply with inorganic magnetic materials, where localized d- or f-orbitals typically lead to weak FM coupling—partially explaining why robust ferromagnetism in 2D systems remains rare.^65,66^

### Spin-Polarized Electronic Structures for FM 2D Frameworks

We further demonstrate that these binary 2D frameworks are FM semiconductors, featuring spin-polarized flat bands and spin-dependent bandgaps (Figures 3 and S20–S43). The flat bands located near the Fermi level correspond to electronic states that are energetically localized yet delocalized in momentum space. Unlike atomic flat bands, which typically arise from dangling bonds with negligible intersite interactions,^71^ these π-electron flat bands originate from correlated molecular orbitals with substantial wave function overlap. Within such bands, electron–electron interactions dominate over kinetic energy, giving rise to a high density of states at the Fermi level and enabling strong correlation phenomena^71^ (Figure 3). The coexistence of flat bands and strong orbital overlap establishes these frameworks as promising platforms for realizing a variety of correlated quantum phases, including fractional quantum Hall states and unconventional superconductivity analogous to those observed in magic-angle twisted bilayer graphene and kagome-lattice materials.^17,72,73^ Similar spin-polarized flat bands have also been reported in Mott–Hubbard AFM insulators, such as [TRI], [PLY], and [TAM].^25,26,34^ However, in these AFM systems, the spin-up and spin-down channels remain energetically degenerate due to preserved inversion and time-reversal symmetries.^34,74^ In sharp contrast, all of the FM 2D frameworks reported here exhibit pronounced spin splitting energy, arising from the inversion-symmetry breaking, which lifts the Kramers degeneracy^75^ (Table 1, Figures 3 and 4d). In such systems, the spin-splitting energies (*E*_split_) range from 1.15 to 2.11 eV (Table 1 and Figure 3). These values far exceed those of previously reported magnetic 2D polymers,^34^ opening opportunities for spintronic applications such as organic spin valves and spin-selective transport devices.

**Figure 4:**
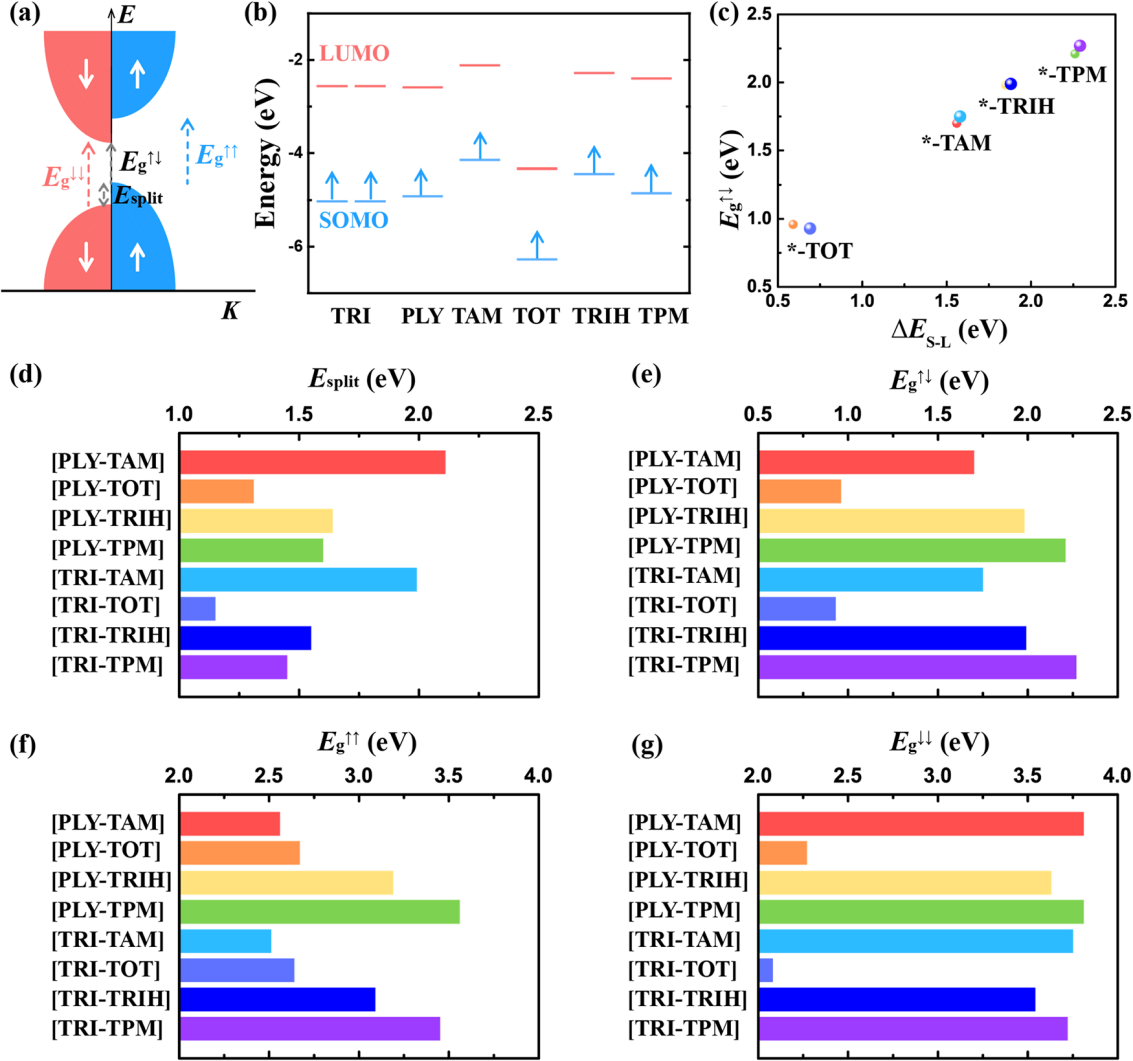
Magnetic properties of FM 2D frameworks. (a) Schematic illustration of spin-polarized band structures, illustrating the definitions of spin-splitting energy (*E*_split_), spin-flip bandgaps (*E*_g_^↑↓^), and spin-polarized bandgaps for spin-up and spin-down channels (*E*_g_^↑↑^ and *E*_g_^↓↓^). (b) Frontier molecular orbital energies of radical monomers, showing singly occupied molecular orbitals (SOMOs, blue) and lowest unoccupied molecular orbitals (LUMOs, red). (c) Correlation between spin-flip bandgap (*E*_g_^↑↓^) and the SOMO–LUMO energy difference (Δ*E*_S–L_) across the FM 2D frameworks. (d–g) Predicted *E*_split_, *E*_g_^↑↓^, *E*_g_^↑↑^, and *E*_g_^↓↓^ for representative FM 2D frameworks.

In addition to their large spin-splitting, these FM 2D frameworks show tunable spin-dependent bandgaps, including both spin-flip and spin-polarized (spin-conserving) transitions (Table 1, Figures 3, and 4e–g). The spin-flip bandgap (*E*_g_^↑↓^) is defined as the lowest energy excitation between states of opposite spin channels and is particularly relevant for electrical gating, where both spin species can be accessed (Figure 4a). In contrast, the spin-polarized bandgaps (*E*_g_^↑↑^ and *E*_g_^↓↓^) correspond to the minimal excitation energies within a single spin channel and thus describe spin-polarized optical transitions (Figure 4a). Across the binary 2D frameworks, *E*_g_^↑↓^ spans a broad range from 0.93 to 2.21 eV (Table 1 and Figure 3). Remarkably, for pairs of polymers containing the same Topology II monomer, the PLY-based and TRI-based frameworks exhibit comparable *E*_g_^↑↓^. For instance, [PLY-TRIH] and [TRI-TRIH] feature similar *E*_g_^↑↓^ of 1.64 and 1.55 eV, respectively (Table 1). This trend indicates that the Topology II unit predominantly governs the spin-flip bandgap (Figure 4b and Table S2). Owing to the staggered alignment between SOMO and the lowest unoccupied molecular orbitals (LUMO) levels of the two monomers, *E*_g_^↑↓^ are primarily determined by the LUMO of the Topology II unit (Figure 4b). Moreover, we identify a positive correlation between the monomer-level SOMO–LUMO energy separation (Δ*E*_S–L_) and *E*_g_^↑↓^, suggesting that spin-flip bandgaps can be rationally tuned through molecular design (Figure 4c). In comparison, *E*_g_^↑↑^ and *E*_g_^↓↓^ are significantly larger, ranging from 2.0 to 3.8 eV, and display a strong dependence on the Topology II monomer (Table 1, Figure 4f,g). These spin-dependent bandgaps enable versatile spin manipulation via external stimuli, such as electrical gating or optical excitation. For instance, in [PLY-TAM], a gate voltage of approximately 1.8 V can shift the Fermi level to selectively activate the spin-down conduction channel, thereby enabling spin-polarized charge transport. Combined with the inherently weak spin–orbit coupling and the long spin coherence times in carbon-based materials, these FM 2D frameworks represent promising platforms for high-fidelity spintronic and quantum information devices.

### Thermal Stability and Feasibility of Ferromagnetic Order in 2D Frameworks

We note the long-standing theoretical argument regarding the existence of FM order in low-dimensional systems. In particular, purely organic materials composed exclusively of light elements (C, H, and O), such as nanographenes and metal-free 2D frameworks, typically show intrinsically weak spin–orbit coupling and negligible magnetic anisotropy.^76,77^ According to the Mermin–Wagner theorem, continuous symmetries preclude long-range magnetic order in infinite 2D systems at any finite temperature.^78^ However, numerous experimental studies have demonstrated robust FM order in finite-sized 1D and 2D systems, such as cobalt atomic chains,^79^ zigzag-edged,^11^ and Janus graphene nanoribbons.^3^ This is because, in finite-sized systems, isotropic 2D magnets can still retain FM order over experimentally accessible scales, corresponding to a magnetic correlation length.^80^ Specifically, the correlation length ξ scales exponentially with the ratio *J*/*T*, following ξ ∝ exp­(*cJ*/*T*), where *c* is a material-dependent constant.^81^ Recent Monte Carlo (MC) simulations demonstrate that in finite-size 2D systems, exchange interactions constitute the dominant driving force for magnetic ordering. Although a true phase transition, corresponding to a well-defined Curie temperature *T*_c_, is absent in the isotropic Heisenberg model in the thermodynamic limit, a characteristic crossover temperature can still be identified, separating a regime of pronounced short-range magnetic order with finite intrinsic magnetization from a fully disordered paramagnetic state. However, for historical reasons, the Curie temperature is widely used in the literature to describe finite-size systems and systems with negligible (or weak) magnetic anisotropy (e.g., Cr_2_Ge_2_Te_6_).^66,82^ It has been shown that the inclusion of magnetic anisotropy only weakly affects this crossover temperature.^78^ Although the intrinsic magnetic anisotropy of the metal-free 2D framework is small, it may be enhanced by proximity effects, as these materials are commonly synthesized on Au(111) substrates with pronounced spin–orbit coupling.^83,84^

To evaluate the thermal stability of the FM ordering and estimate the crossover temperature *T*_*x*_ in these 2D frameworks, we performed MC simulations to determine their Curie temperatures (*T*_c_), as described in the computational details of Supporting Information (Figure 5a). In finite-sized systems, these *T*_c_ values provide a reasonable estimate of the crossover temperature *T*_*x*_ at which magnetic order and pronounced magnetization emerge. As illustrated in Figure 5b,c, the calculated saturated magnetic moment approaches the ideal value of 2 μB per unit cell (25.2 emu/g), consistent with fully spin-polarized π-radicals in [PLY-TAM] frameworks. For comparison, CrI_3_ has a saturated magnetization of 6 μB per unit cell (38.7 emu/g). This intrinsic value stands in stark contrast to the much smaller magnetization associated with dilute transition-metal impurities in organic materials, which often arise from residual catalyst during synthesis.^85^ As evidenced by Table 1 and Figures 5a and S45–S46, all directly coupled systems, including [PLY-TAM], [PLY-TOT], [PLY-TRIH], [PLY-TPM], [TRI-TAM], [TRI-TOT], [TRI-TRIH], and [TRI-TPM], exhibit *T*_c_ well above room temperature, ranging from 338 to 570 K. These remarkably high values arise primarily from the exceptionally large FM coupling in these systems. As summarized in Table 1, *T*_c_ exhibits a positive correlation with magnetic coupling *J*. Larger *J* values render the magnetic order more resistant to thermal disruption, resulting in higher Curie or crossover temperatures.^86,87^ Among them, [TRI-TAM] shows the highest *T*_c_ of 570 K, closely followed by [PLY-TAM] with *T*_c_ = 556 K, driven by its exceptionally large *J* of 127 meV (Figures 5a and S45, S46 and Table 1). These *T*_c_ values are unprecedented for metal-free materials and substantially exceed those of well-known inorganic 2D van der Waals ferromagnets, including Cr_2_Ge_2_Te_6_ (*J* ≈ 4 meV, *T*_c_ ≈ 66 K) and CrI_3_ (*J* ≈ 4 meV, *T*_c_ ≈ 45 K).^65,66^ Consequently, in systems with large *J*, as in the 2D frameworks reported here, magnetic order persists up to a crossover temperature that remains appreciable over mesoscopic and even macroscopic length scales, far exceeding typical device dimensions. This has been demonstrated by recent large-scale MC simulations of isotropic 2D van der Waals magnets.^80^ This work shows that finite-size effects enable the stabilization of finite-temperature magnetic order in realistic laboratory flakes without requiring magnetic anisotropy. Given that state-of-the-art organic 2D frameworks synthesized via bottom-up on-surface methods typically yield crystalline domains ranging from several nanometers to several tens of nanometers,^22,62,63^ the theoretical predictions presented in this work are directly relevant to current experimental capabilities and hold strong potential for validation on existing nanotechnology platforms.

**Figure 5:**
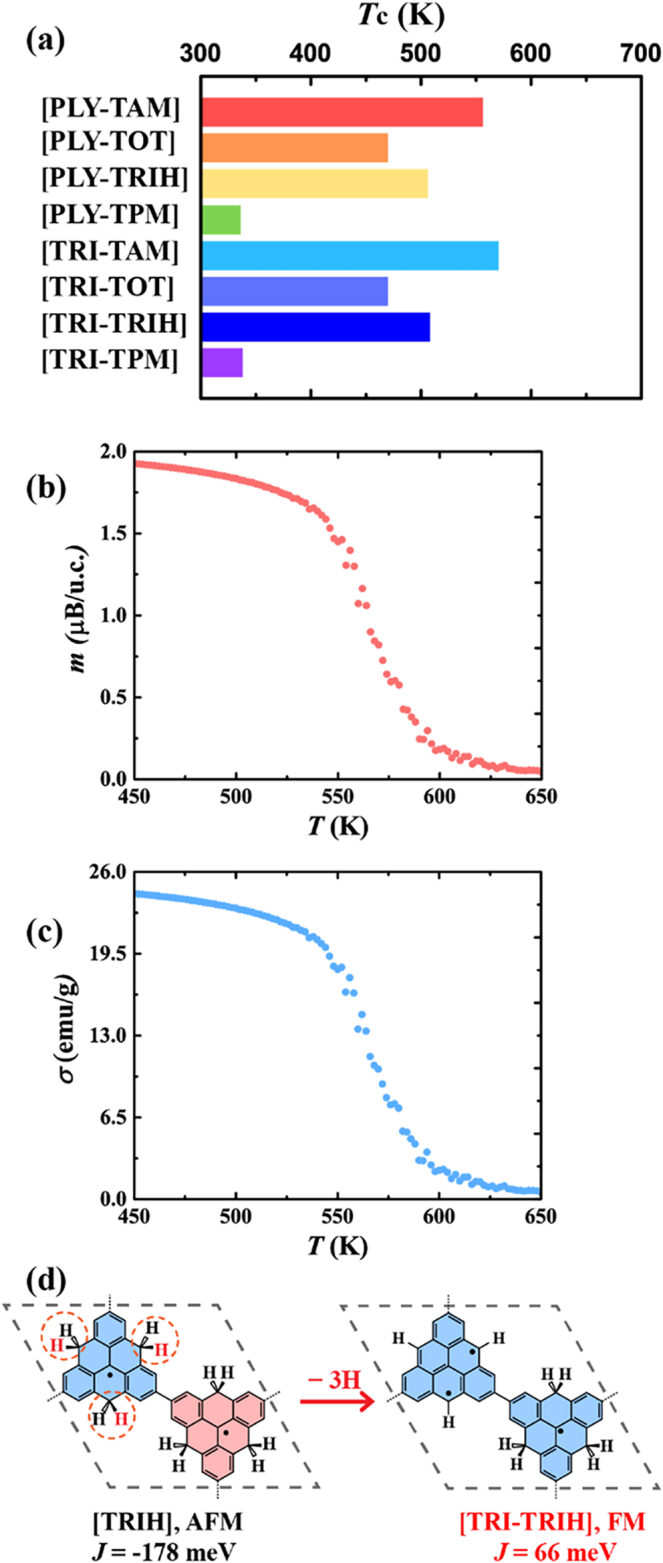
(a) Predicted Curie temperatures (*T*_c_) for representative FM 2D frameworks, indicating the crossover (Curie) temperature. Temperature-dependent magnetization from Monte Carlo simulations of [PLY-TAM] in units of (b) Bohr magnetons per unit cell and (c) emu/g. (d) Conceptual approach for realizing FM 2D frameworks via selective dehydrogenation, using a scanning tunneling microscope (STM).

Regarding experimental feasibility, these 2D frameworks can be realized through two strategies: (i) conventional solution-phase synthesis using Suzuki–Miyaura cross-coupling reaction between the corresponding halide and organoboron precursors^88^ (Figures S47 and S48) or (ii) on-surface synthesis via tip-induced, site-selective dehydrogenation of homogeneous diamagnetic 2D frameworks precursors using a scanning tunneling microscope (STM) (Figure 5d). The latter approach has been extensively demonstrated in previous studies for a broad range of π-radical monomers, including Clar’s goblet and Olympicene, and for their polymeric structures.^4,89,90^ Notably, Suzuki–Miyaura coupling has been extensively used for the synthesis of structurally well-defined 1D conjugated polymers with controlled repeat-unit sequences, such as alternating donor–acceptor polymers.^91,92^ Given the inherent air- and moisture-sensitivity of these polycyclic π-radical systems, device fabrication must be conducted under strictly controlled environments, typically ultrahigh vacuum, inert atmosphere, or through encapsulation with h-BN overlayers to prevent rapid quenching.^76,77^ Importantly, our design strategy for half-filled, bipartite lattices, in agreement with Lieb’s theorem, can also extend to FM 1D spin chains. This capability enables the experimental exploration of 1D strongly correlated phenomena, such as spin-wave propagation and magnon excitations, within highly tunable organic platforms.

## Conclusions

In summary, we have established a mixed-topology design strategy to achieve room-temperature ferromagnetism in π-conjugated 2D frameworks. By covalently linking π-radical monomers with complementary sublattice topologies, we rationally break the inversion symmetry and enforce parallel spin alignment across the lattice. This design yields strong through-bond FM coupling, driven by substantial spin–orbital overlap and dominant direct magnetic exchange while simultaneously suppressing electronic coupling and AFM superexchange. Through systematic first-principles calculations of 32 binary 2D frameworks, we identify a novel class of metal-free FM semiconductors with remarkable magnetic characteristics, including FM coupling up to 127 meV and Curie temperatures exceeding 550 K. These materials further feature spin-polarized flat bands, large spin-splitting energies (1–2 eV), and tunable spin-dependent bandgaps (0.9–3.8 eV). More broadly, although many approaches that induce sublattice imbalance can introduce nonzero *S*, the resulting *J* values can be too small to sustain long-range FM order, particularly in systems with long linkers or spatially separated spin centers, which often yield only paramagnetic behavior. In contrast, our mixed-topology strategy provides a robust and experimentally accessible route to achieving strong FM coupling. Our findings establish an effective framework for designing room-temperature, metal-free ferromagnets and chart a path forward for integrating lightweight and flexible quantum materials into next-generation spintronic, optoelectronic, and quantum information technologies.

## Supplementary Material



## Notes

Computational methods, crystal structures, band structures, density of states, spin density distributions, and Monte Carlo simulation results for all 2D frameworks (PDF)
