# Supplementary material for: Metal-Free Ferromagnetism in Triangulene Two-Dimensional Frameworks
Source: J Am Chem Soc. 2026 Mar 30;148(13):13822–33. doi: 10.1021/jacs.5c21206 (PMC13067274; doi:10.1021/jacs.5c21206)
Supplement: Supplementary file 1 [file ja5c21206_si_001.pdf]

Metal-free ferromagnetism in triangulene two-dimensional frameworks

Hongde Yu and Thomas Heine

Corresponding author: Thomas Heine, thomas.heine@tu-dresden.de

This PDF file includes:

Computational methods
Figure S1. Radicals and diamagnetic precursors for realizing extended architectures via Ullmann coupling.
Figure S2. Spin densities of (hetero-)triangulene monomers.
Figure S3. Chemical structures of [TPM-TRIH] and [TRI-PLY] 2D crystals.
Figure S4. Magnetic properties of [TRI-PLY].
Figure S5. Magnetic properties of [TRIH-TPM].
Figure S6. Chemical structures of [PLY-TAM], [PLY-TOT] and [PLY-TRIH] and [PLY-TPM].
Figure S7. Chemical structures of [PLY-CC-TAM], [PLY-CC-TOT] and [PLY-CC-TRIH] and [PLY-CC-TPM].
Figure S8. Chemical structures of [PLY-CCCC-TAM], [PLY-CCCC-TOT] and [PLY-CCCC-TRIH] and [PLY-CCCC-TPM].
Figure S9. Chemical structures of [PLY-Ph-TAM], [PLY-Ph-TOT] and [PLY- Ph-TRIH] and [PLY-Ph-TPM].
Figure S10. Chemical structures of [TRI-TAM], [TRI-TOT] and [TRI-TRIH] and [TRI-TPM].
Figure S11. Chemical structures of [TRI-CC-TAM], [TRI-CC-TOT] and [TRI-CC-TRIH] and [TRI-CC-TPM].
Figure S12. Chemical structures of [TRI-CCCC-TAM], [TRI-CCCC-TOT] and [TRI-CCCC-TRIH] and [TRI-CCCC-TPM].
Figure S13. Chemical structures of [TRI-Ph-TAM], [TRI-Ph-TOT] and [TRI- Ph-TRIH] and [TRI-Ph-TPM].
Figure S14. Structure illustration of [TRI-TAM], representing the lattice of TRI-based 2D frameworks.
Figure S15. Structures and energies of different isomers.
Figure S16. Schematic illustration of the chemical structures for the alternating monomer arrangement and non-alternating (non-uniform) configurations in a binary 2D framework.
Figure S17. Snapshots of the crystal structures of a [PLY-TAM] supercell (2×2) obtained from *ab initio* molecular dynamics simulations in the NVT ensemble after 10 ps.
Figure S18. The spin density isosurfaces for the AFM and FM states of [PLY-TAM], [PLY-TOT], [PLY-TRIH], [PLY-TPM].

Figure S19. The spin density isosurfaces for the AFM and FM states of [TRI-TAM], [TRI-TOT], [TRI-TRIH], and [TRI-TPM].
Figure S20. Magnetic properties of [PLY-CC-TAM].
Figure S21. Magnetic properties of [PLY-CC-TOT].
Figure S22. Magnetic properties of [PLY-CC-TRIH].
Figure S23. Magnetic properties of [PLY-CC-TPM].
Figure S24. Magnetic properties of [PLY-CCCC-TAM].
Figure S25. Magnetic properties of [PLY-CCCC-TOT].
Figure S26. Magnetic properties of [PLY-CCCC-TRIH].
Figure S27. Magnetic properties of [PLY-CCCC-TPM].
Figure S28. Magnetic properties of [PLY-Ph-TAM].
Figure S29. Magnetic properties of [PLY-Ph-TOT].
Figure S30. Magnetic properties of [PLY-Ph-TRIH].
Figure S31. Magnetic properties of [PLY-Ph-TPM].
Figure S32. Magnetic properties of [TRI-CC-TAM].
Figure S33. Magnetic properties of [TRI-CC-TOT].
Figure S34. Magnetic properties of [TRI-CC-TRIH].
Figure S35. Magnetic properties of [TRI-CC-TPM].
Figure S36. Magnetic properties of [TRI-CCCC-TAM].
Figure S37. Magnetic properties of [TRI-CCCC-TOT].
Figure S38. Magnetic properties of [TRI-CCCC-TRIH].
Figure S39. Magnetic properties of [TRI-CCCC-TPM].
Figure S40. Magnetic properties of [TRI-Ph-TAM].
Figure S41. Magnetic properties of [TRI-Ph-TOT].
Figure S42. Magnetic properties of [TRI-Ph-TRIH].
Figure S43. Magnetic properties of [TRI-Ph-TPM].
Figure S44. Band structures and density of states (DOS) of the diamagnetic states for [PLY-TAM], [PLY-TOT], [PLY-TRIH], and [PLY-TPM].
Figure S45. Monte Carlo simulations for [PLY-TAM], [PLY-TOT], [PLY-TRIH], and [PLY-TPM].
Figure S46. Monte Carlo simulations for [TRI-TAM], [TRI-TOT], [TRI-TRIH], and [TRI-TPM].
Figure S47. Radicals and diamagnetic precursors for realizing binary 2D frameworks via Suzuki-Miyaura coupling reaction.
Figure S48. Proposed synthetic routes toward binary 2D covalent frameworks.
Figure S49. Illustration of the spin arrangements for the FM, AFM and zigzag-type AFM (AFM-zz) configurations.
Figure S50. Spin density distribution of [PLY-TAM] for FM, AFM, and zigzag-type AFM (AFM-zz) spin configurations

Table S1. Magnetic coupling for nearest-neighbor and next-nearest-neighbor spin pairs in representative ferromagnetic 2D frameworks.
Table S2. Frontier orbital energies of building blocks.
Table S3. Magnetic couplings calculated at the PBE0 and HSE06 levels.

Computational methods:

Density functional theory (DFT) calculations:

The structural optimizations of all O2DCs were performed within the framework of density functional theory (DFT) using the Vienna Ab Initio Simulation Package (VASP 5.4.4).[1] A plane-wave cutoff energy of 400 eV was adopted, and the electron-ion interactions were described by the projector augmented wave (PAW) method.[2] The Perdew-Burke-Ernzerhof (PBE)[3] exchange-correlation functional within the generalized gradient approximation was employed, together with Grimme's D3 dispersion correction to account for van der Waals interactions.[4] The optimization criteria were set to a force threshold of 0.005 eV $Å^{-1}$, and an electronic energy convergence of 10^{-5} eV per self-consistent field cycle. A Monkhorst-Pack *k*-point grid of 8 × 8 × 1 was used for sampling the Brillouin zone during structural relaxations. Hybrid functional calculations were subsequently performed at the PBE0 level using the POB-TZVP basis set to obtain the band structure, density of states (DOS) and spin density, as implemented in the CRYSTAL17 software package,[5] with a k-point mesh of 16 × 16 × 1. Based on our prior benchmark study, the predicted magnetic couplings (J) of PBE0 functional closely align with high-level multi-reference calculations in metal-free systems.[6] Additionally, we provide Heyd–Scuseria–Ernzerhof (HSE06) results for comparison, which yields comparable J values. (Table S3) Given that the systems comprise only C, H, and O elements, spin-orbit coupling (SOC) effects were neglected. J values were evaluated based on the energy difference between the ferromagnetic (FM) and antiferromagnetic (AFM) states, following the expression $J = (E_{AFM}\text{-}E_{FM})/2zS_1S_2$, where z is the number of nearest-neighbor spin pairs per spin center, (e.g., z = 3 for honeycomb lattices), and S_1 and S_2 are the spin quantum numbers of the spin centers in the unit cell, (e.g., S = 1 for TRI, S = 1/2 for PLY, TOT, TAM, TRIH and TPM). For FM (high-spin) configurations, we used unrestricted Kohn-Sham DFT (UKS) calculations with proper spin polarization. For AFM or ferrimagnetic (i.e., low-spin) configurations, we used broken-symmetry DFT (BS-DFT) calculations, in which spin symmetry is explicitly broken to obtain an approximate representation of the AFM (open-shell singlet) state. BS-DFT has been widely used in computational chemistry.[7–10] To evaluate the next-nearest-neighbor magnetic coupling (J_2), the total energies of three spin configurations, i.e., ferromagnetic (FM), antiferromagnetic (AFM), and zigzag-type antiferromagnetic (AFM-zz), were calculated using a $\sqrt{2} \times \sqrt{2}$ supercell. As shown in Figures S49 and S50, the supercell contains four spin sites, including two sites with spin quantum number S_1 and two sites with spin quantum number S_2. Based on the HDVV Hamiltonian (eqn. 1), the total energies of the three spin arrangements can be expressed as

$$\boldsymbol{E_{FM} = -1/2 \times [4 \times 3 \times J_1 S_1 S_2 + 2 \times 6 \times J_2 (S_1^2 + S_2^2)]}$$
$$\boldsymbol{E_{AFM} = -1/2 \times [4 \times 3 \times (-J_1) S_1 S_2 + 2 \times 6 \times J_2 (S_1^2 + S_2^2)]}$$
$$\boldsymbol{E_{AFM-zz} = -1/2 \times [4 \times J_1 S_1 S_2 + 2 \times 2 \times (-J_2)(S_1^2 + S_2^2)]}$$

where J_1 and J_2 denote the nearest-neighbor and next-nearest-neighbor magnetic coupling constants, respectively. From these expressions, the J_2 value can be derived from the energy differences between the AFM and AFM-zz states as:

$$J_2 = [(E_{AFM} - E_{AFM-zz}) - 8J_1S_iS_j]/(-8(S_1^2 + S_2^2)).$$

The electronic coupling parameter (t) was approximated from the bandwidth (W) using $W = 2zt$, extracted from the band structures of diamagnetic states. The on-site energy offset in Table 1 is calculated by the SOMO energy difference of radical building blocks. Overlap integrals between adjacent monomers were computed using dimer models within the unit cell via the Multiwfn program.[11] Frontier orbital energies (SOMO and LUMO) were obtained from PBE0/def2-TZVP single-point calculations using the Gaussian 16 software package.[12,13] All optimized geometries necessary to reproduce the results are available through the Zenodo repository.[14]

Molecular dynamics simulations:
Ab initio molecular dynamics simulations were performed on a 2 × 2 supercell of [PLY-TAM] using the CP2K software package.[15] The Goedecker-Teter-Hutter (GTH) pseudopotentials[16,17] were employed in conjunction with double-ζ valence polarized basis sets (DZVP-MOLOPT-SR-GTH). The SCF convergence criterion was set to 1×10^{-5} for the density matrix. Orbital transformation (OT)[18] was applied for SCF optimization. Molecular dynamics were carried out in the NVT ensemble using the canonical sampling through velocity rescaling (CSVR) thermostat. Each trajectory consisted of 10,000 steps with a time step of 1 fs, resulting in a total simulation time of 10 ps.

Monte Carlo (MC) simulations:

Given the high computational cost associated with performing Monte Carlo simulations of the isotropic Heisenberg model in finite-size systems, we employed the critical temperature of the infinite 2D Ising model, T_c, as a reference temperature to qualitatively estimate the crossover temperature range from short-range magnetic order to the disordered regime in the isotropic sample with finite-size. In the thermodynamic limit, the 2D Ising model exhibits a true phase transition, whereas in a finite $L \times L$ system this transition is rounded into a broadened crossover due to finite-size effects, with the associated crossover temperature T_x (L) gradually approaching T_c as the system size increases. In realistic finite systems, although the isotropic 2D Heisenberg model does not support true infinite magnetic order, exchange-driven short-range correlation can remain stable over a finite temperature window.[19] Because the Ising model represents, under the same exchange interaction, the discrete-symmetry limit most favorable for stabilizing magnetic order, its critical temperature provides a reasonable empirical upper bound for the temperature range over which short-range order persists in the isotropic Heisenberg model with finite size.[20] For experimentally relevant micro-scale flakes with finite lateral dimensions, this estimate offers a useful order-of-magnitude reference for assessing magnetic stability. We have used MC simulations with the metropolis algorithm to determine the Curie temperature of all the FM O2DCs. The specific electronic heat capacity (C_v) has been calculated by $C_v = \frac{<E^2> - <E>^2}{k_B T^2}$. We have used a 40 × 40 × 1 supercell with periodic boundary conditions in the simulation and

calculated 50 trajectories at every temperature. For each trajectory, we have calculated 100000000 steps while the last 90000000 steps are used to average and generate the data.

Supplementary Note. Comparison of Ferromagnetic Coupling Strength in Metal-Free 2D Frameworks and CrI_3.

The FM coupling in the present metal-free 2D frameworks is described using the standard Heisenberg Hamiltonian, $\hat{H}=-\sum_{<i,\,j>} J\hat{S}_i\hat{S}_j$, consistent with the convention widely adopted for both organic radical-based and inorganic *d*-electron systems.[10,21–23] Under this definition, the nearest-neighbor FM coupling constant J in monolayer CrI_3 is typically reported to be approximately 2-4 meV, depending on the DFT methodology and the Hubbard U parameter.[24,25]

The overall magnetic performance depends on multiple factors, including the raw J values, spin quantum numbers (S = 1/2 or mixed S = 1/2 and S = 1 in binary 2D frameworks versus S = 3/2 for Cr^{3+} in CrI_3) and coordination number z. Within the mean-field approximation for a homogeneous spin system, the Curie temperature scales as $T_c \propto J z S(S+1)$.[26]

To facilitate a more meaningful comparison of the intrinsic FM coupling strength between metal-free 2D frameworks and CrI_3, the effective quantity $J z S(S+1)$ is evaluated here. For the representative [PLY-TAM] framework, the honeycomb lattice yields z = 3 and S = 1/2 for the radical centers, resulting in a larger $J z S(S+1)$ value of 286 meV, compared to that of 45 meV in CrI_3 (where z = 3, S = 3/2, and typical J = 4 meV). The effective quantity for [PLY-TAM] is roughly five times larger, driven primarily by the significantly strong nearest-neighbor J.

This substantial FM J in the metal-free 2D frameworks originates from direct through-bond overlap of adjacent π-orbitals, which enables efficient direct exchange. In contrast, magnetic coupling in inorganic ferromagnets such as CrI_3 relies on indirect superexchange mediated by ligands (e.g., Cr-I-Cr pathways), which is inherently less efficient owing to the spatial separation of localized Cr 3*d* orbitals.

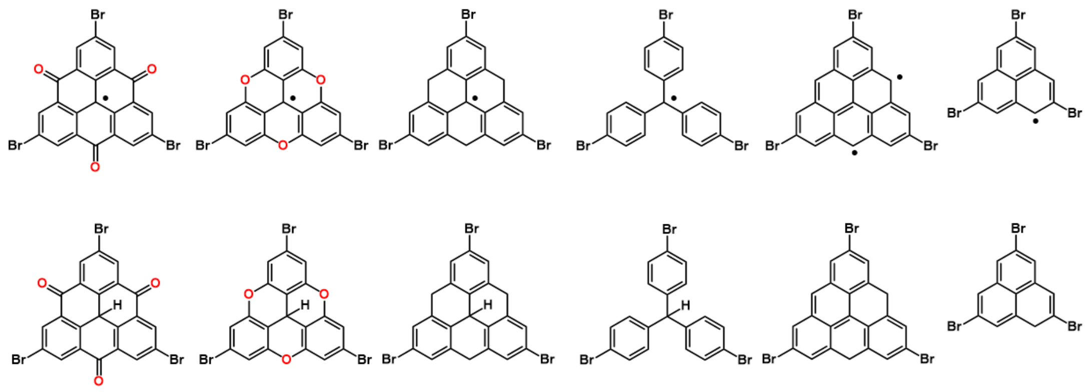

Figure S1. Radicals and diamagnetic precursors for realizing extended architectures via Ullmann coupling.

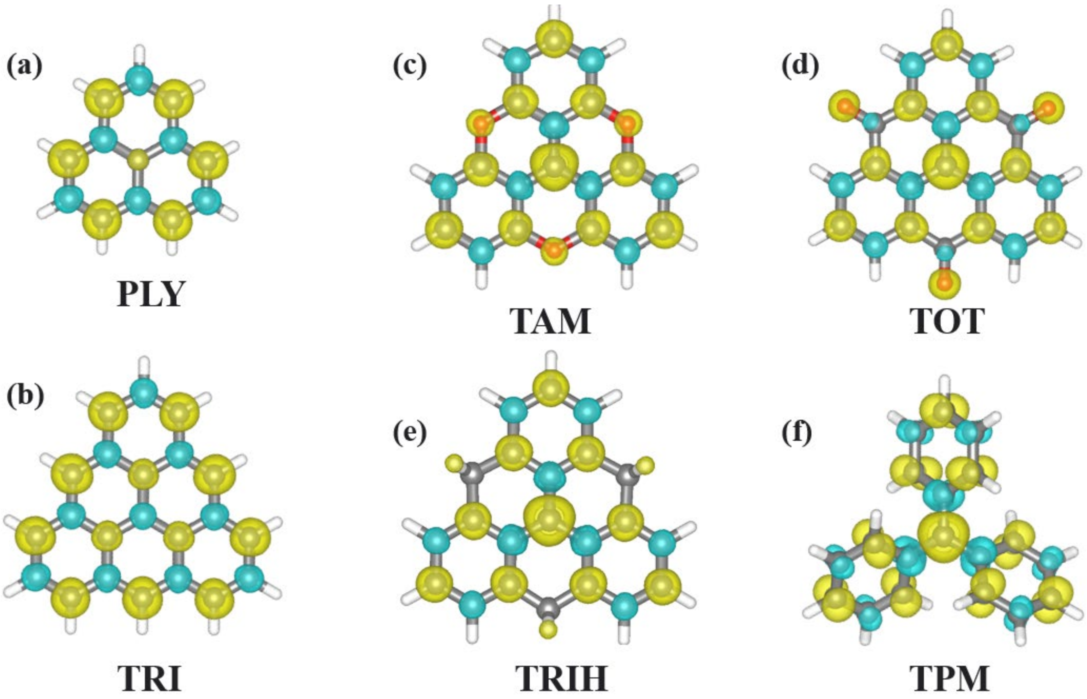

Figure S2. Spin densities of (hetero-)triangulene monomers.

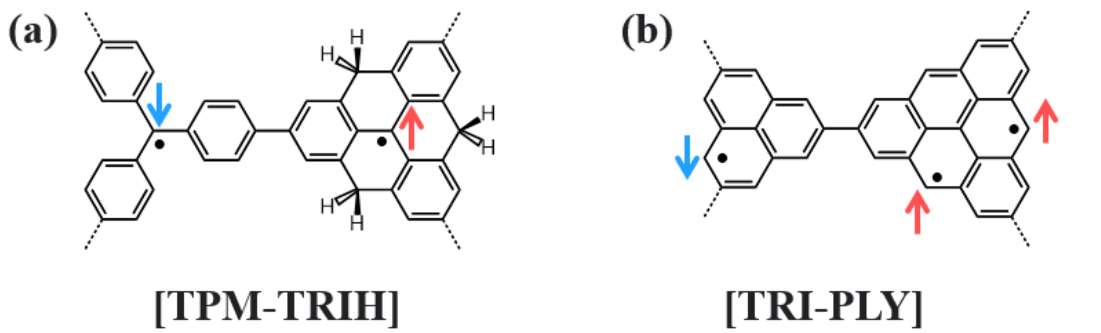

Figure S3. Chemical structures of [TPM-TRIH] and [TRI-PLY] 2D polymer. The monomers are antiferromagnetically coupling according to Lieb's theorem.

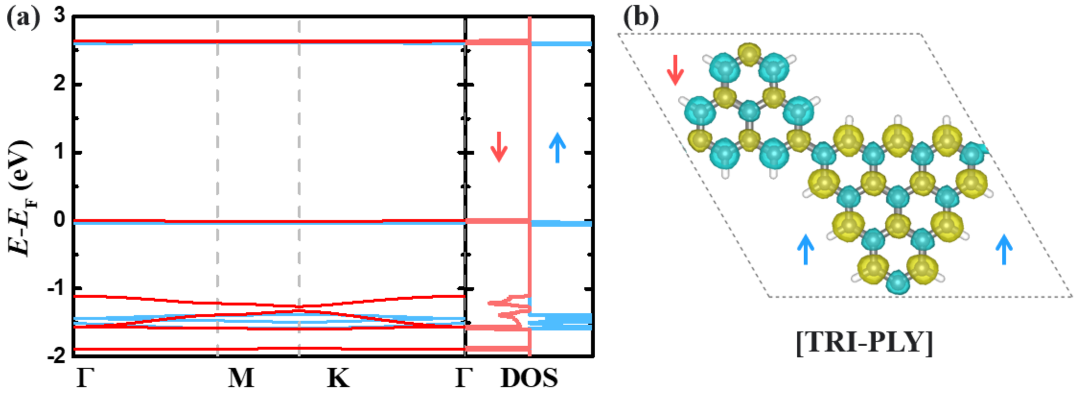

Figure S4. Magnetic properties of [TRI-PLY] for (a) spin-polarized band structure and (b) spin density calculated at the PBE0 level.

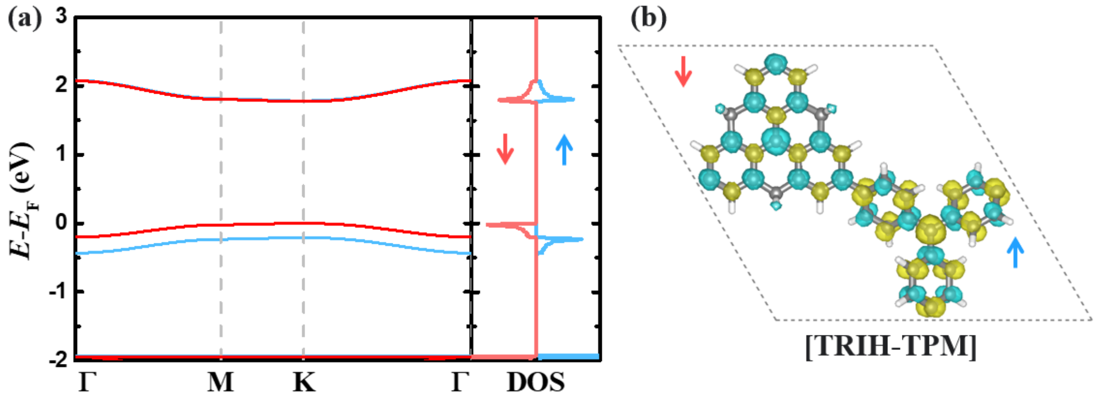

Figure S5. Magnetic properties of [TRIH-TPM] for (a) spin-polarized band structure and (b) spin density calculated at the PBE0 level.

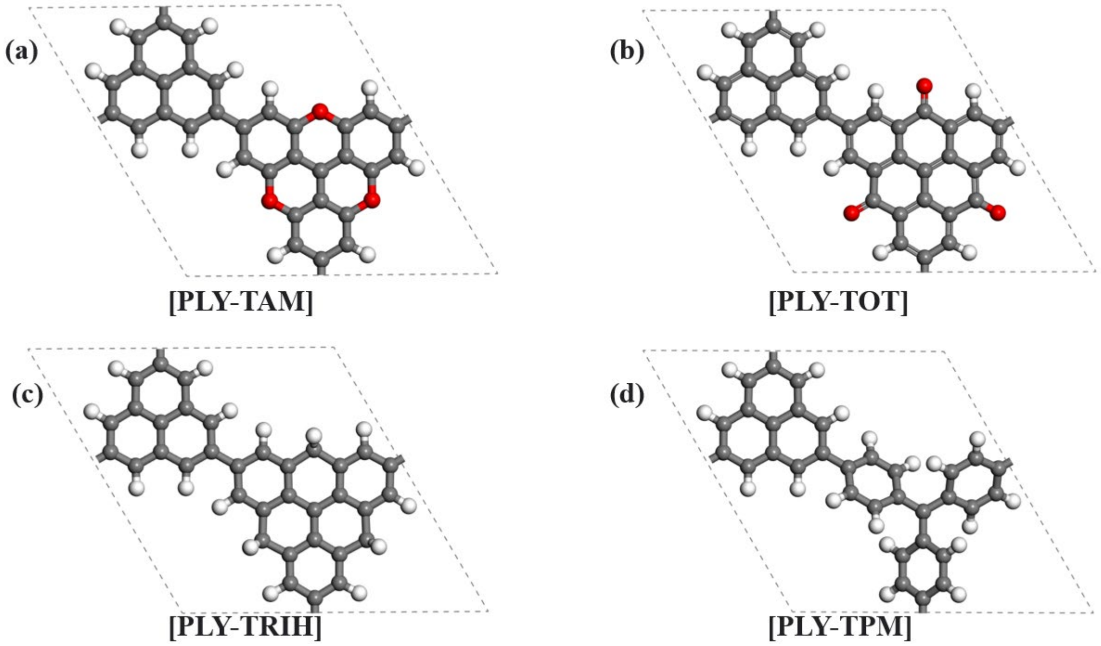

Figure S6. Chemical structures of [PLY-TAM], [PLY-TOT] and [PLY-TRIH] and [PLY-TPM]. (a)-(d) correspond to [PLY-TAM], [PLY-TOT] and [PLY-TRIH] and [PLY-TPM], respectively.

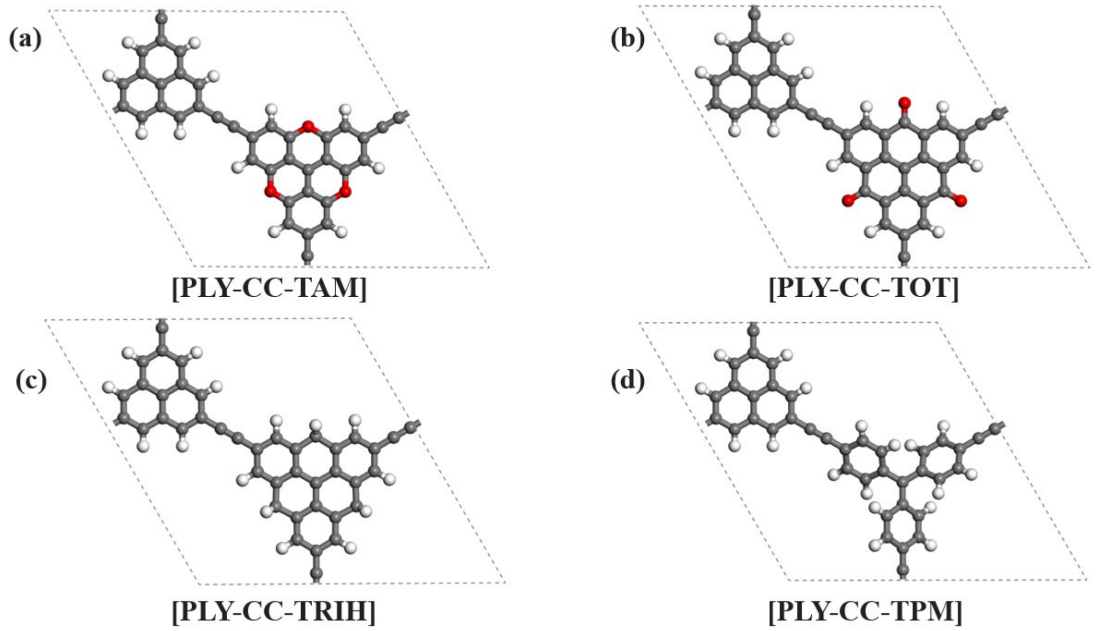

Figure S7. Chemical structures of [PLY-CC-TAM], [PLY-CC-TOT] and [PLY-CC-TRIH] and [PLY-CC-TPM]. (a)-(d) correspond to [PLY-CC-TAM], [PLY-CC-TOT] and [PLY-CC-TRIH] and [PLY-CC-TPM], respectively.

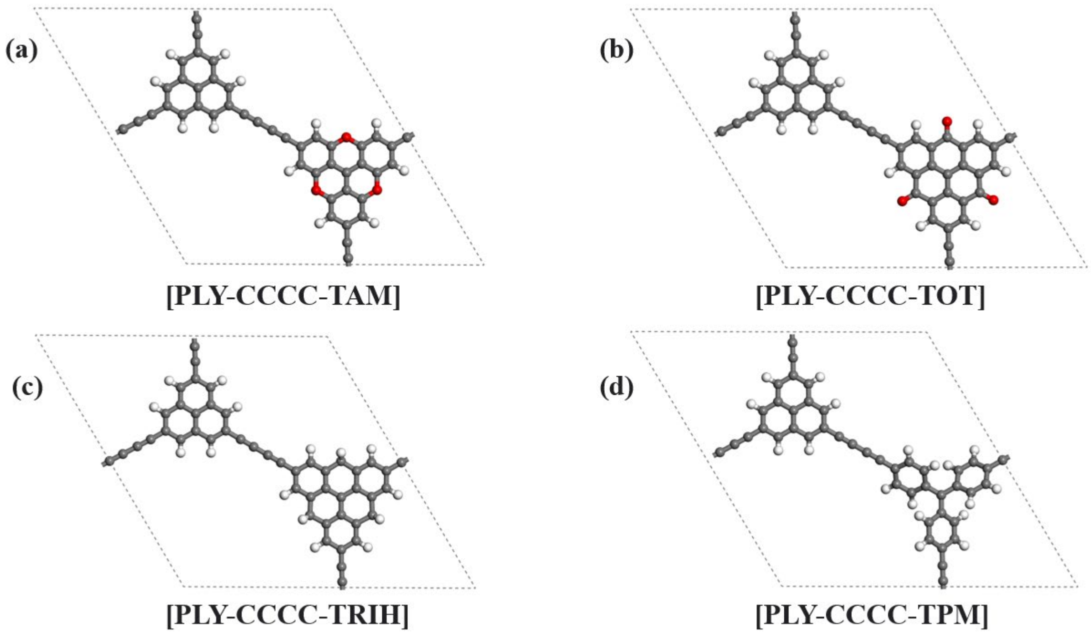

Figure S8. Chemical structures of [PLY-CCCC-TAM], [PLY-CCCC-TOT] and [PLY-CCCC-TRIH] and [PLY-CCCC-TPM]. (a)-(d) correspond to [PLY-CCCC-TAM], [PLY-CCCC-TOT] and [PLY-CCCC-TRIH] and [PLY-CCCC-TPM], respectively.

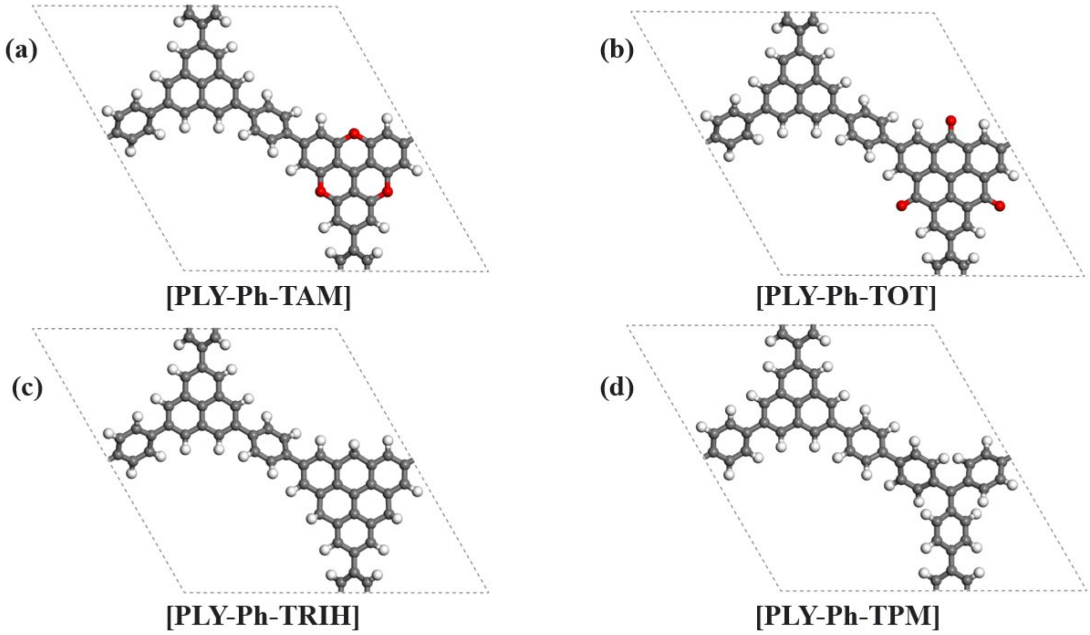

Figure S9. Chemical structures of [PLY-Ph-TAM], [PLY-Ph-TOT] and [PLY- Ph-TRIH] and [PLY-Ph-TPM]. (a)-(d) correspond to [PLY-Ph-TAM], [PLY-Ph-TOT] and [PLY-Ph-TRIH] and [PLY-Ph-TPM], respectively.

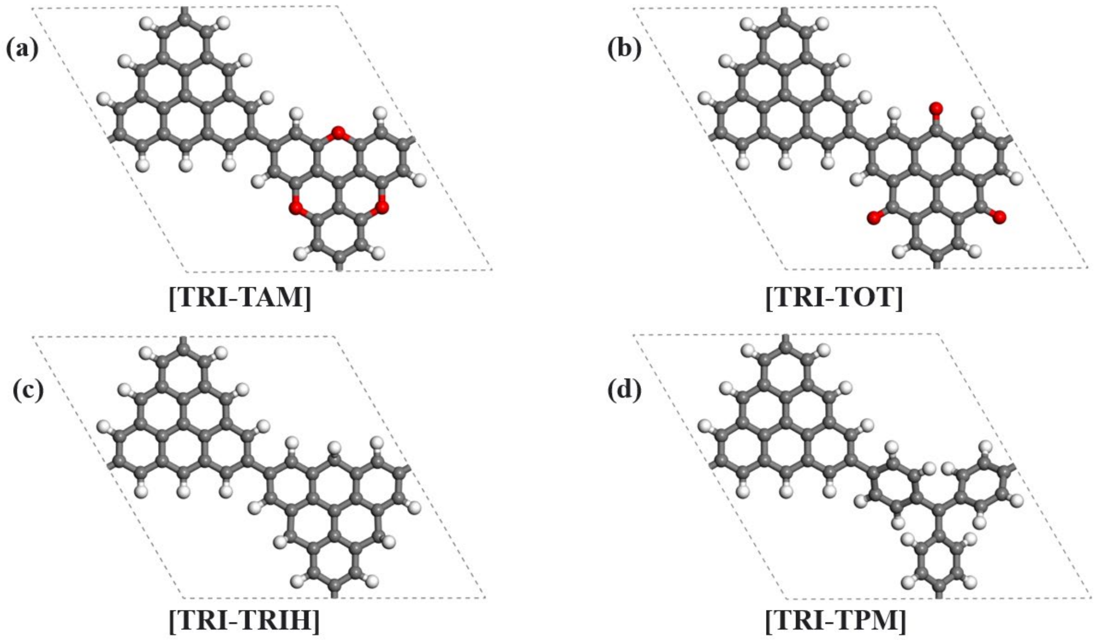

Figure S10. Chemical structures of [TRI-TAM], [TRI-TOT] and [TRI-TRIH] and [TRI-TPM]. (a)-(d) correspond to [TRI-TAM], [TRI-TOT] and [TRI-TRIH] and [TRI-TPM], respectively.

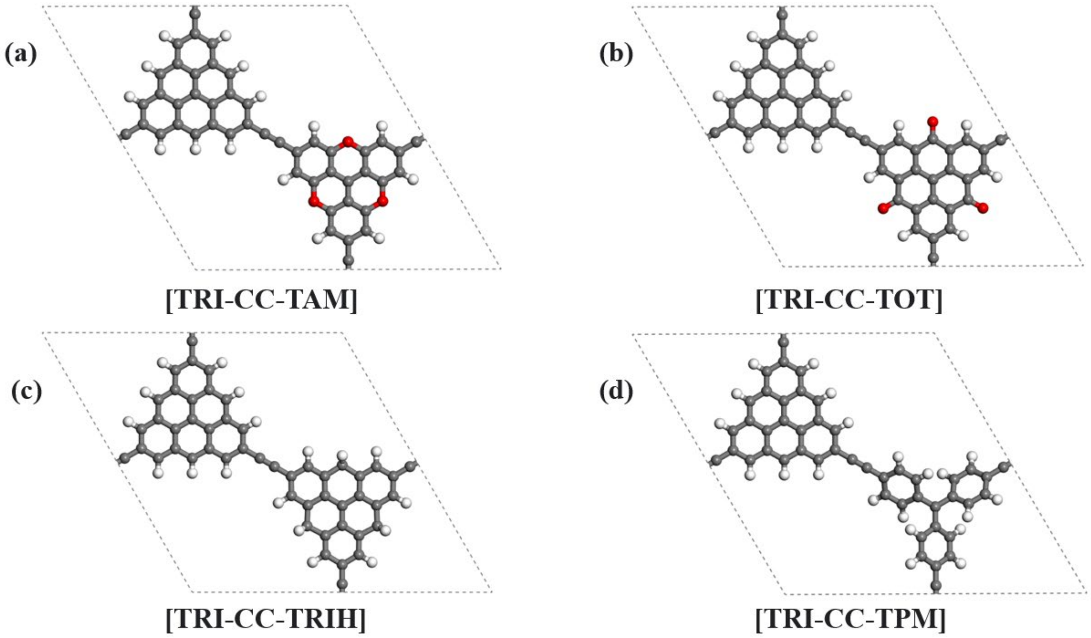

Figure S11. Chemical structures of [TRI-CC-TAM], [TRI-CC-TOT] and [TRI-CC-TRIH] and [TRI-CC-TPM]. (a)-(d) correspond to [TRI-CC-TAM], [TRI-CC-TOT] and [TRI-CC-TRIH] and [TRI-CC-TPM], respectively.

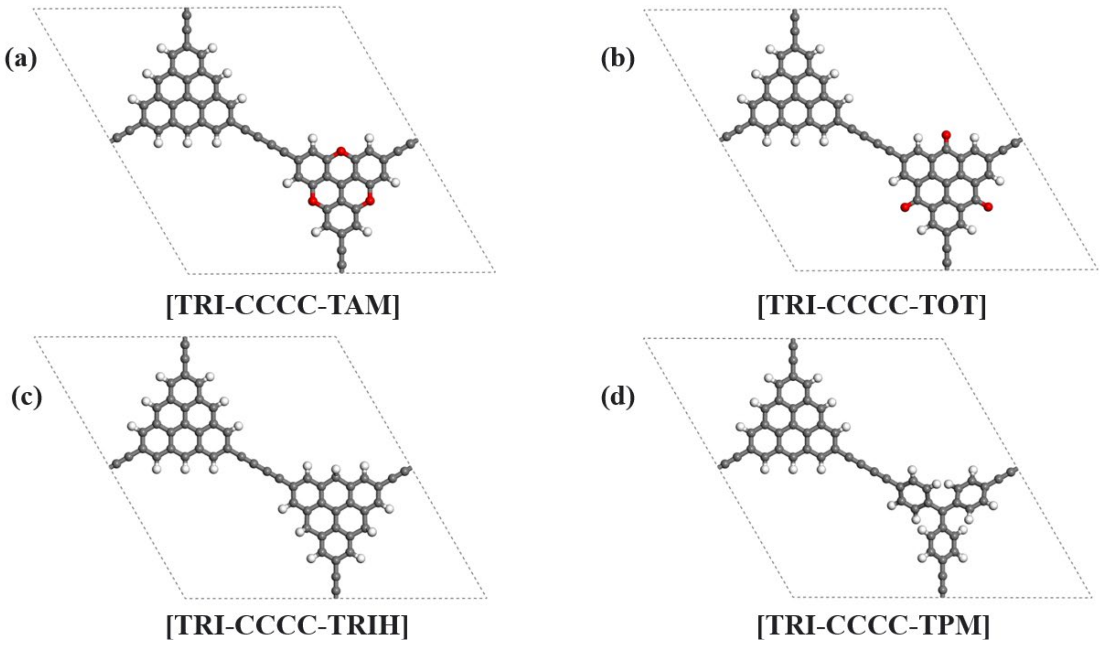

Figure S12. Chemical structures of [TRI-CCCC-TAM], [TRI-CCCC-TOT] and [TRI-CCCC-TRIH] and [TRI-CCCC-TPM]. (a)-(d) correspond to [TRI-CCCC-TAM], [TRI-CCCC-TOT] and [TRI-CCCC-TRIH] and [TRI-CCCC-TPM], respectively.

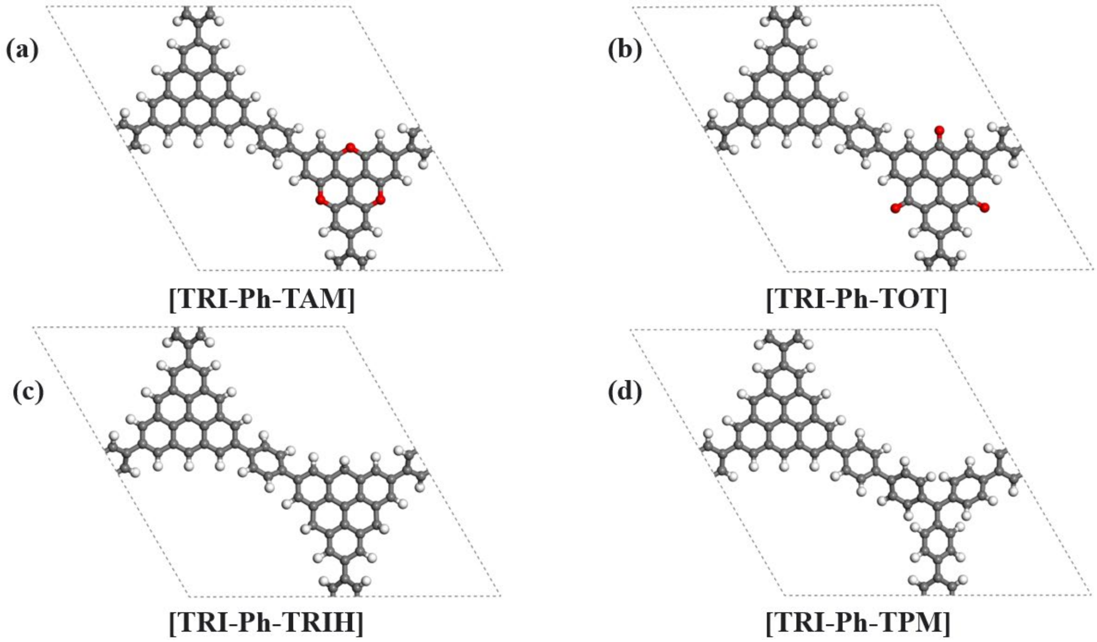

Figure S13. Chemical structures of [TRI-Ph-TAM], [TRI-Ph-TOT] and [TRI- Ph-TRIH] and [TRI-Ph-TPM]. (a)-(d) correspond to [TRI-Ph-TAM], [TRI-Ph-TOT] and [TRI-Ph-TRIH] and [TRI-Ph-TPM], respectively.

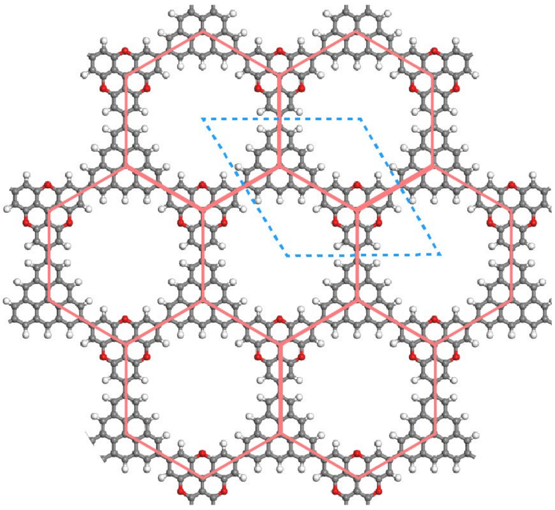

Figure S14. Structure illustration of [TRI-TAM], representing the lattice of TRI-based 2D frameworks.

(a) (b)

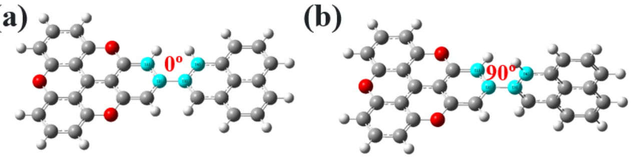

Planar dimer | Twisted dimer

(c)

	Total Energy
**Planar dimer (2D)**	**0**
**Twisted dimer (3D)**	**7.4 kJ/mol**

(d) (e)

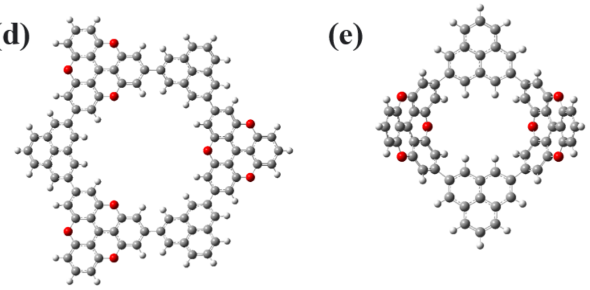

Six-membered ring (honeycomb lattice) | **Four-membered ring (square lattice)**

(f)

	Total Energy / formula (dimer)
**Six-membered ring**	**0**
**Four-membered ring**	**75.4 kJ/mol**

Figure S15. Structures and energies of different isomers. Planar (φ = 0º) and twisted dimer (φ = 90º) represents the 2D and 3D isomers, respectively. Six-membered and four-membered rings represent honeycomb and square lattices, respectively.

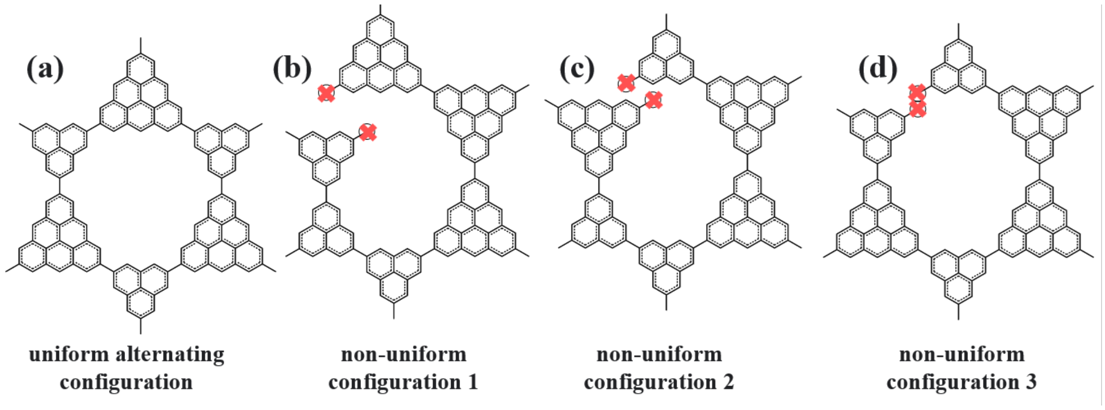

Figure S16. Schematic illustration of the chemical structures for the alternating monomer arrangement and non-alternating (non-uniform) configurations in a binary 2D framework.

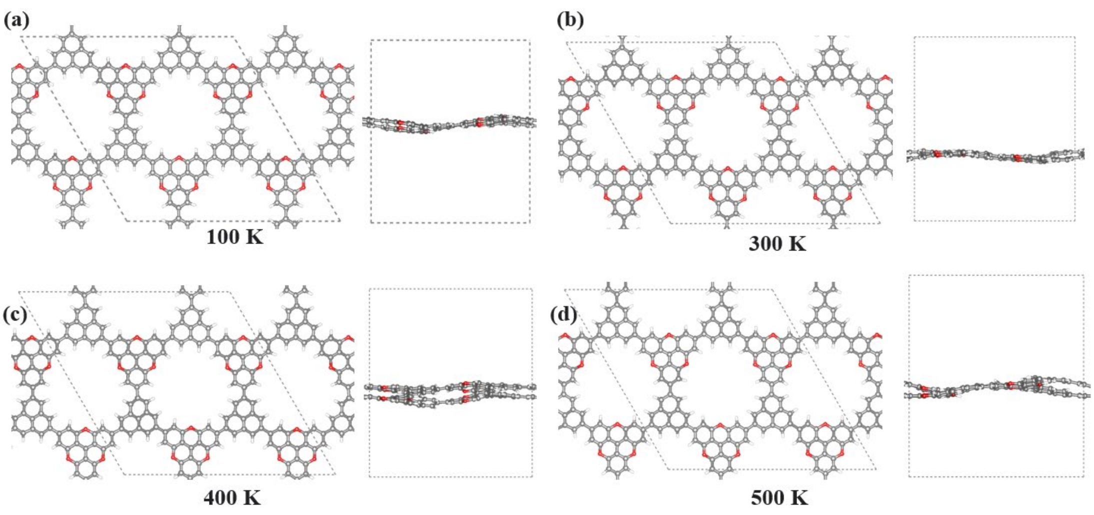

Figure S17. Snapshots of the crystal structures of a [PLY-TAM] supercell (2×2) obtained from *ab initio* molecular dynamics simulations in the NVT ensemble after 10 ps at (a) 100 K, (b) 300 K, (c) 400 K, and (d) 500 K. Each panel shows both top and side views.

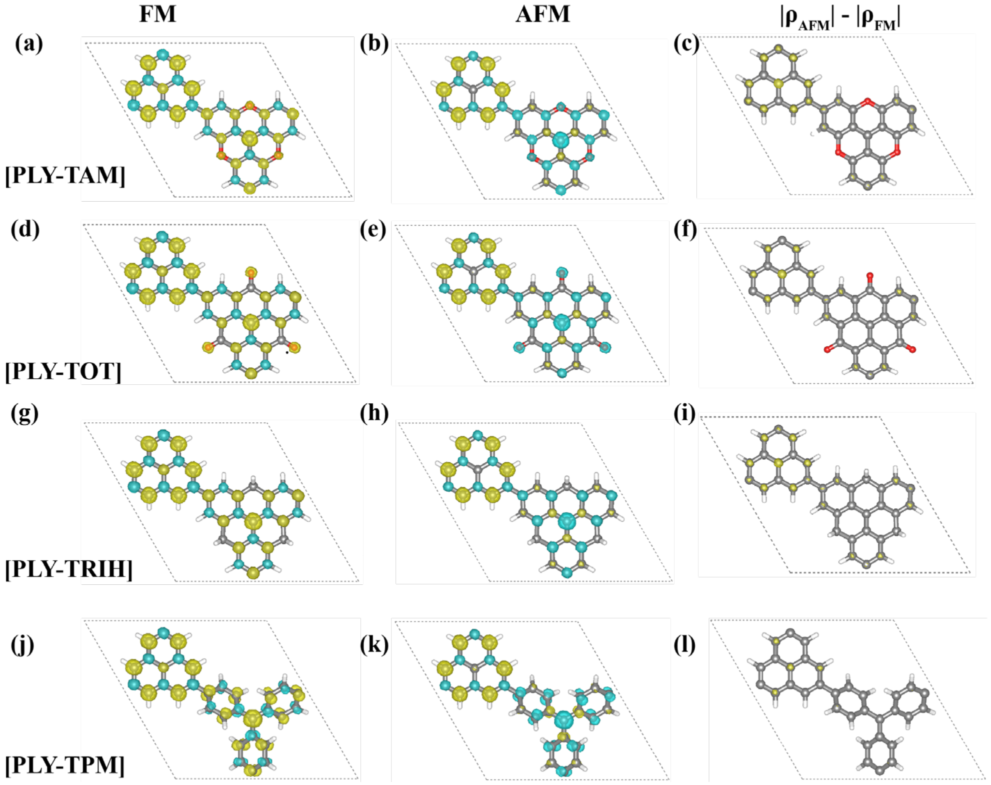

Figure S18. The spin density isosurfaces (isovalue = 0.005 e/bohr^3) for the AFM and FM states of [PLY-TAM], [PLY-TOT], [PLY-TRIH], [PLY-TPM]. Yellow and blue isosurfaces represent up and down spin density, respectively. For comparison, the corresponding $|\rho_{AFM}| - |\rho_{FM}|$ plots are included to illustrate their differences.

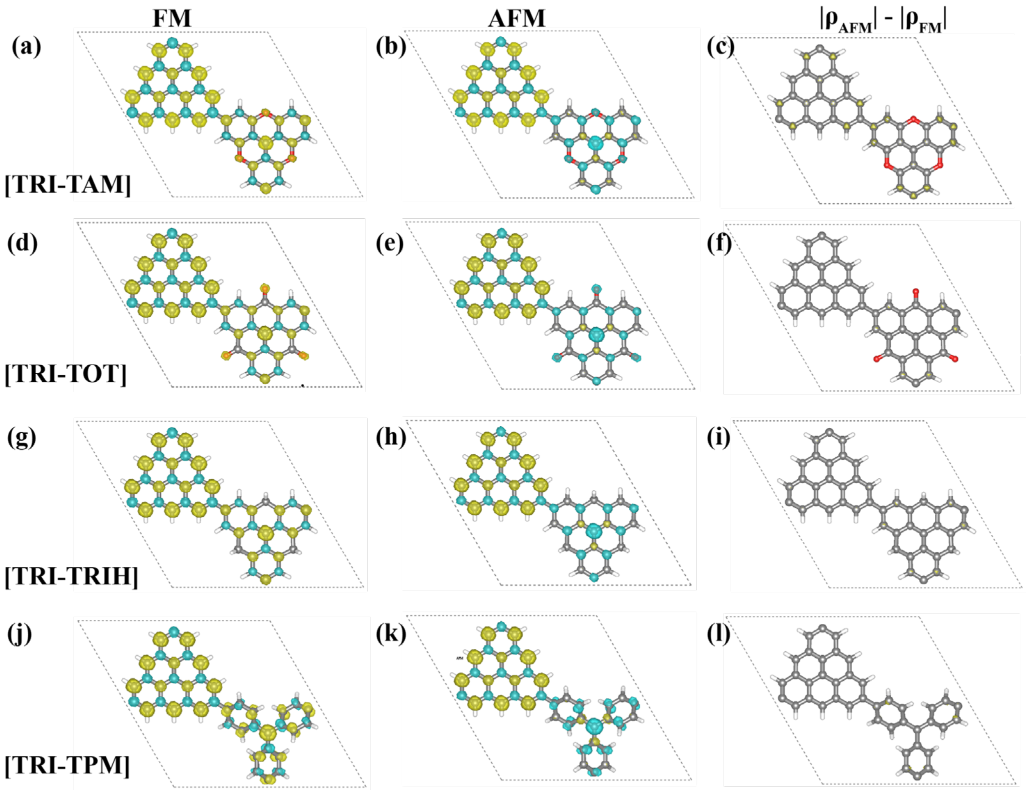

Figure S19. The spin density isosurfaces (isovalue = 0.005 e/bohr^3) for the AFM and FM states of [TRI-TAM], [TRI-TOT], [TRI-TRIH], and [TRI-TPM]. Yellow and blue isosurfaces represent up and down spin density, respectively. For comparison, the corresponding $|\rho_{AFM}|$ - $|\rho_{FM}|$ plots are included to illustrate their differences.

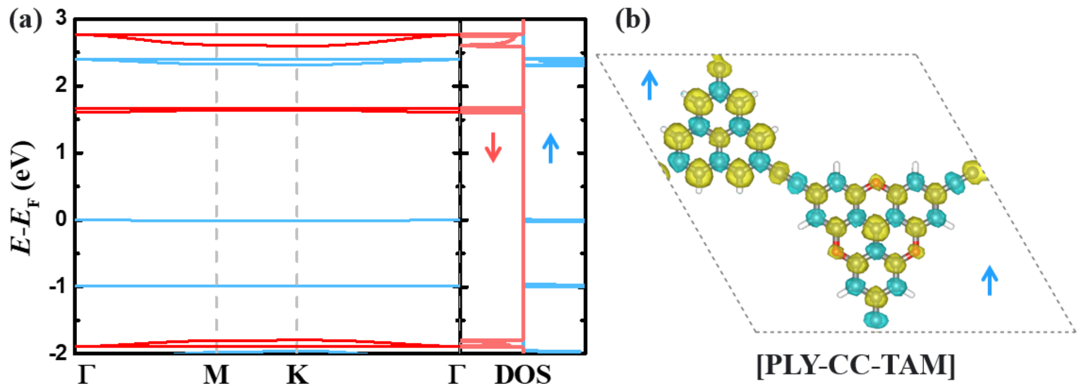

Figure S20. Magnetic properties of [PLY-CC-TAM] for (a) spin-polarized band structure and (b) spin density calculated at the PBE0 level.

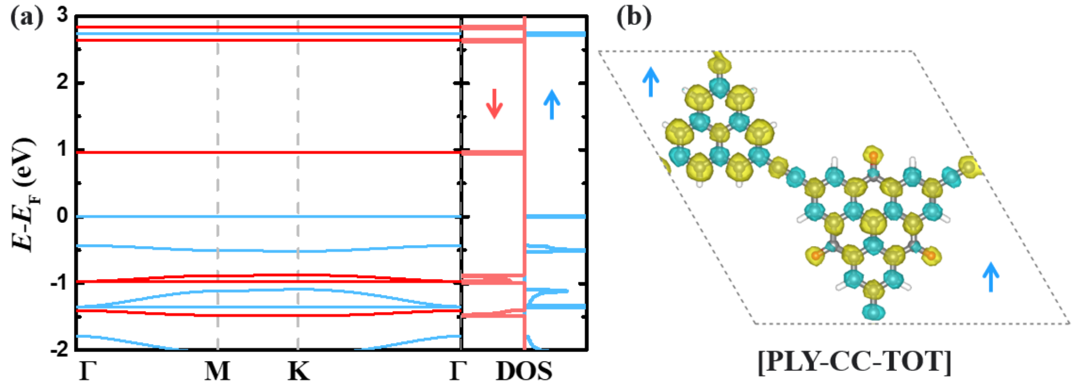

Figure S21. Magnetic properties of [PLY-CC-TOT] for (a) spin-polarized band structure and (b) spin density calculated at the PBE0 level.

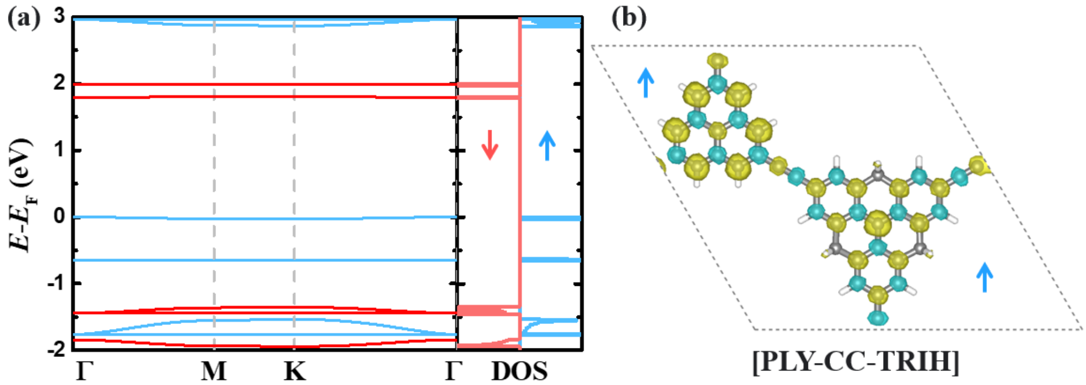

Figure S22. Magnetic properties of [PLY-CC-TRIH] for (a) spin-polarized band structure and (b) spin density calculated at the PBE0 level.

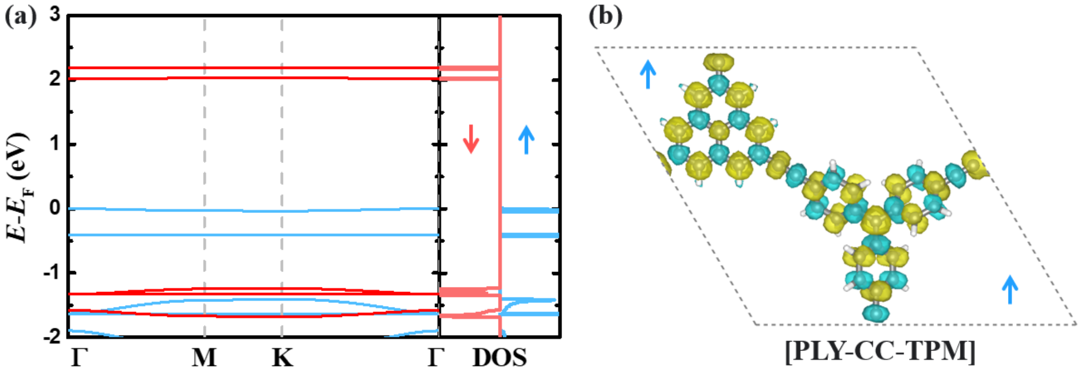

Figure S23. Magnetic properties of [PLY-CC-TPM] for (a) spin-polarized band structure and (b) spin density calculated at the PBE0 level.

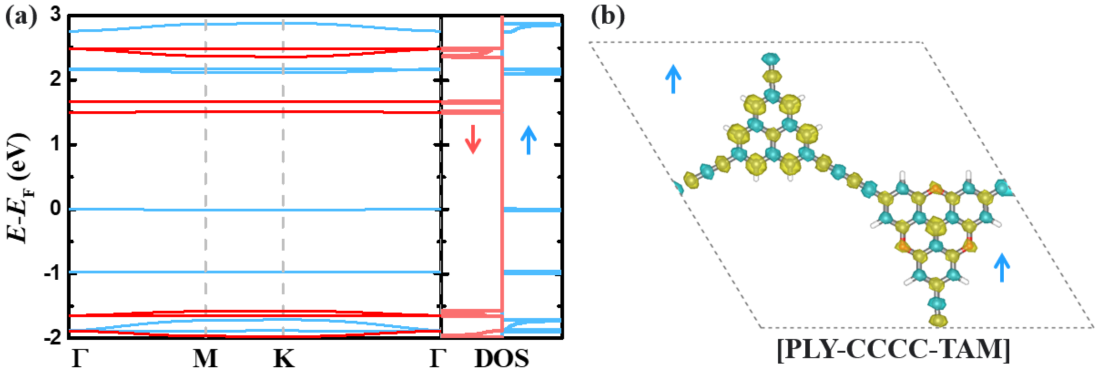

Figure S24. Magnetic properties of [PLY-CCCC-TAM] for (a) spin-polarized band structure and (b) spin density calculated at the PBE0 level.

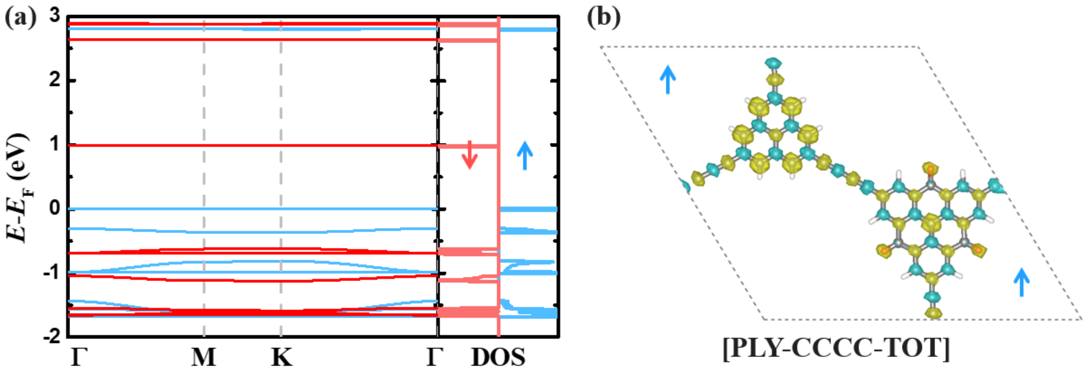

Figure S25. Magnetic properties of [PLY-CCCC-TOT] for (a) spin-polarized band structure and (b) spin density calculated at the PBE0 level.

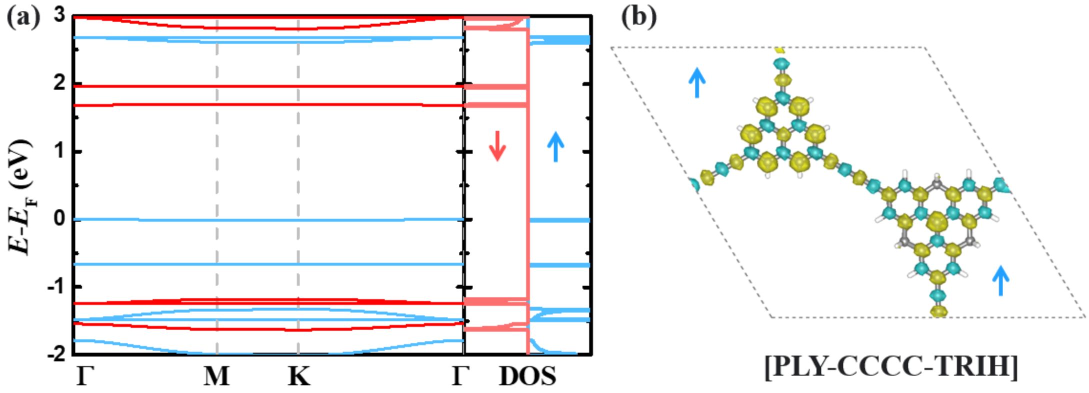

Figure S26. Magnetic properties of [PLY-CCCC-TRIH] for (a) spin-polarized band structure and (b) spin density calculated at the PBE0 level.

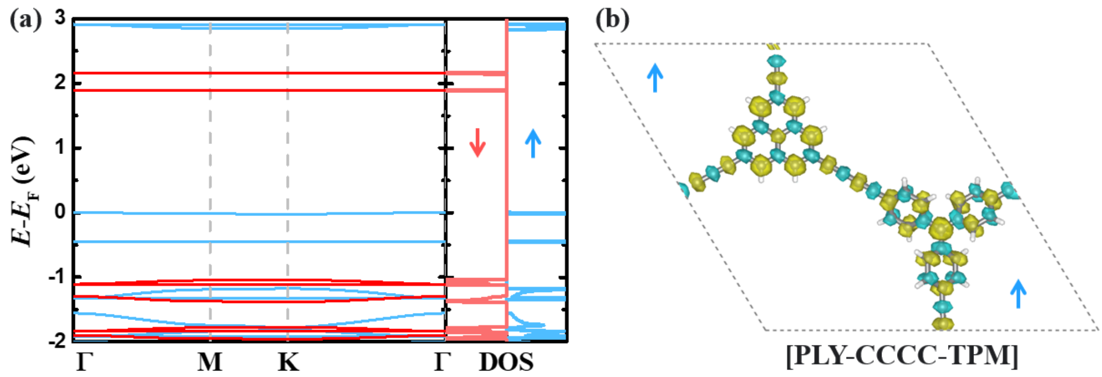

Figure S27. Magnetic properties of [PLY-CCCC-TPM] for (a) spin-polarized band structure and (b) spin density calculated at the PBE0 level.

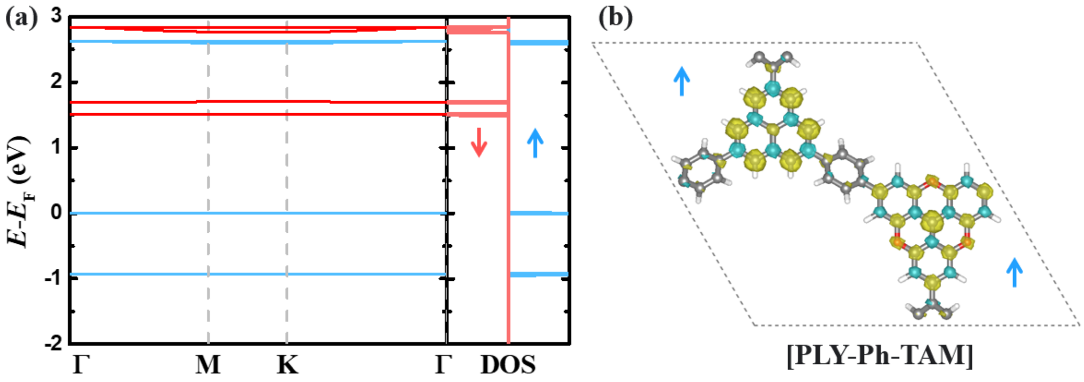

Figure S28. Magnetic properties of [PLY-Ph-TAM] for (a) spin-polarized band structure and (b) spin density calculated at the PBE0 level.

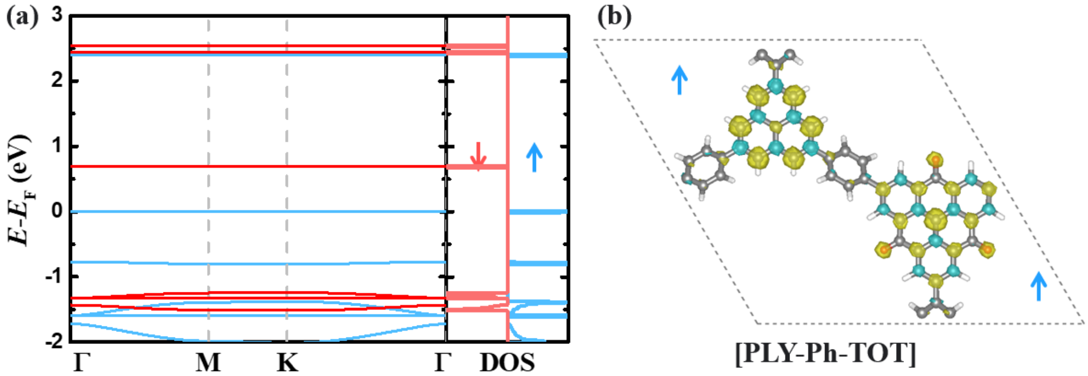

Figure S29. Magnetic properties of [PLY-Ph-TOT] for (a) spin-polarized band structure and (b) spin density calculated at the PBE0 level.

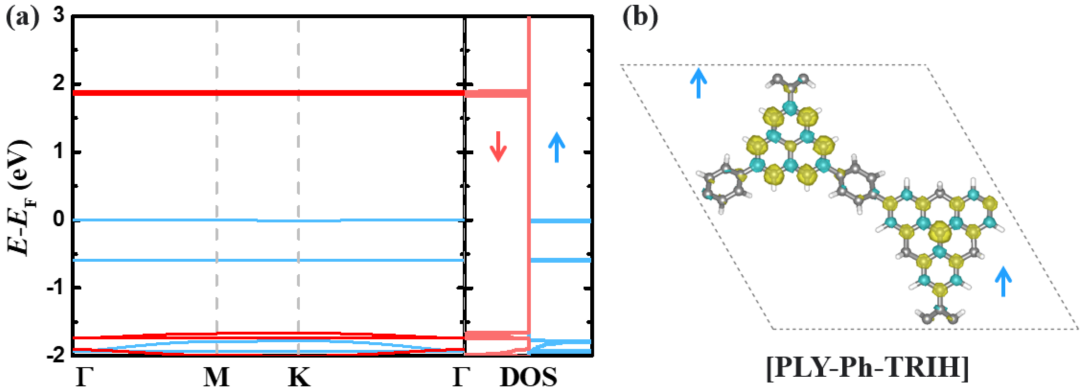

Figure S30. Magnetic properties of [PLY-Ph-TRIH] for (a) spin-polarized band structure and (b) spin density calculated at the PBE0 level.

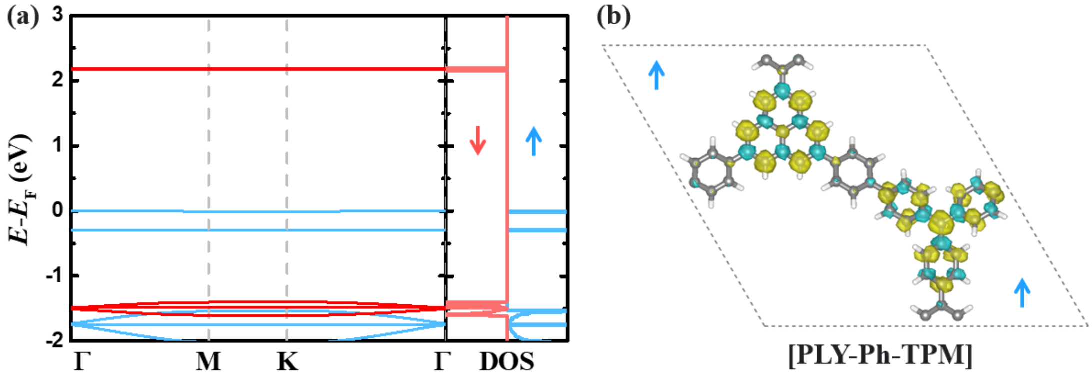

Figure S31. Magnetic properties of [PLY-Ph-TPM] for (a) spin-polarized band structure and (b) spin density calculated at the PBE0 level.

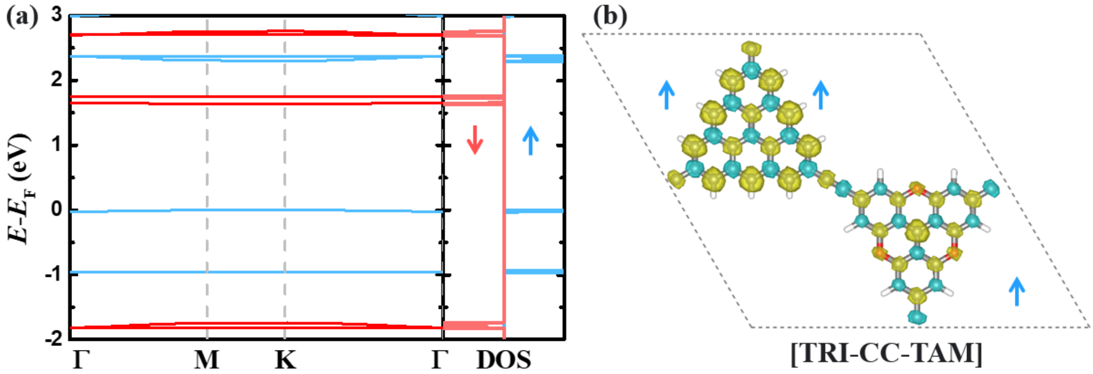

Figure S32. Magnetic properties of [TRI-CC-TAM] for (a) spin-polarized band structure and (b) spin density calculated at the PBE0 level.

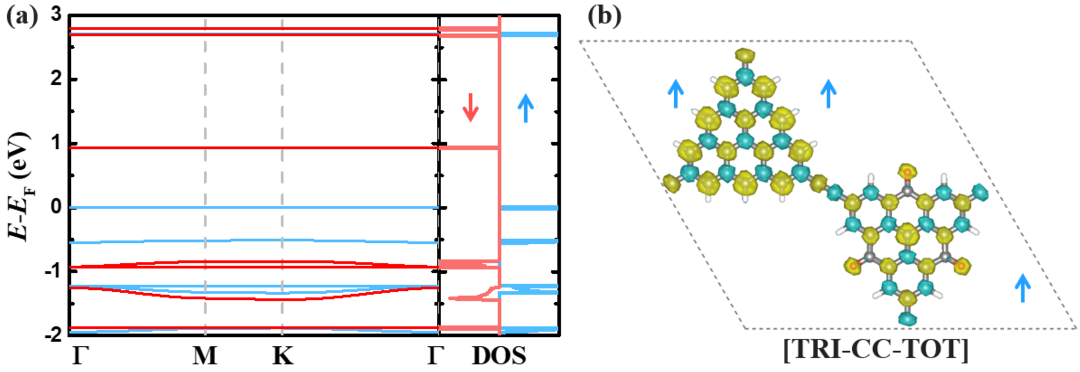

Figure S33. Magnetic properties of [TRI-CC-TOT] for (a) spin-polarized band structure and (b) spin density calculated at the PBE0 level.

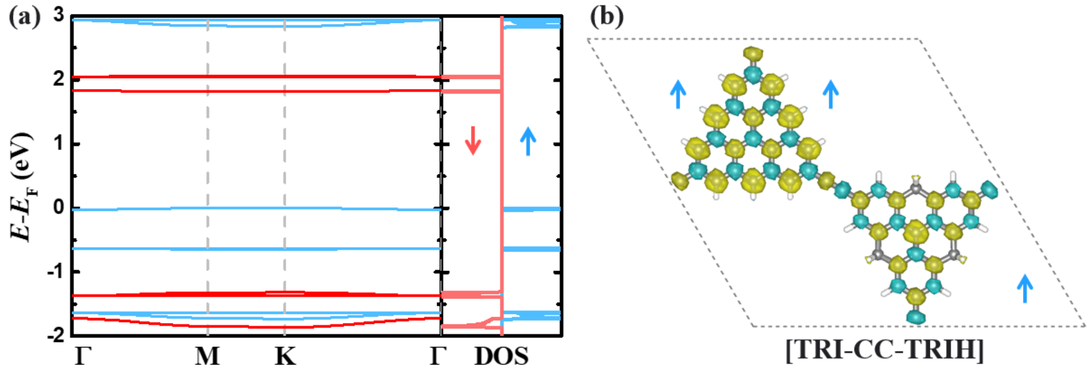

Figure S34. Magnetic properties of [TRI-CC-TRIH] for (a) spin-polarized band structure and (b) spin density calculated at the PBE0 level.

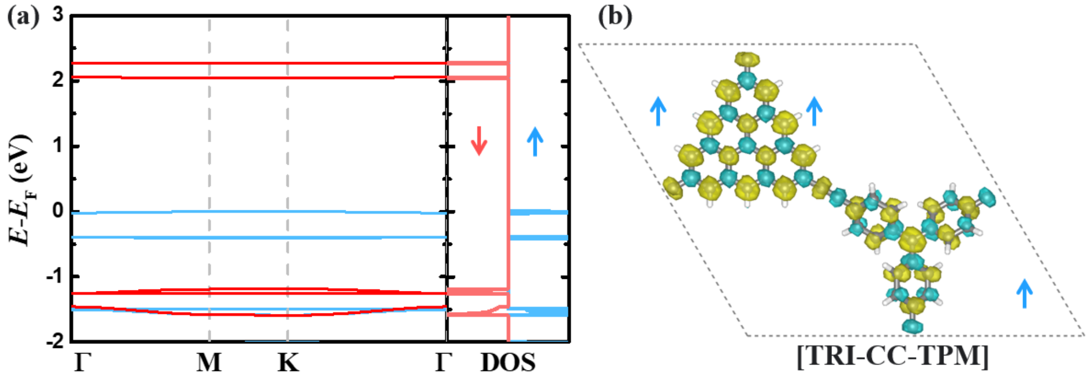

Figure S35. Magnetic properties of [TRI-CC-TPM] for (a) spin-polarized band structure and (b) spin density calculated at the PBE0 level.

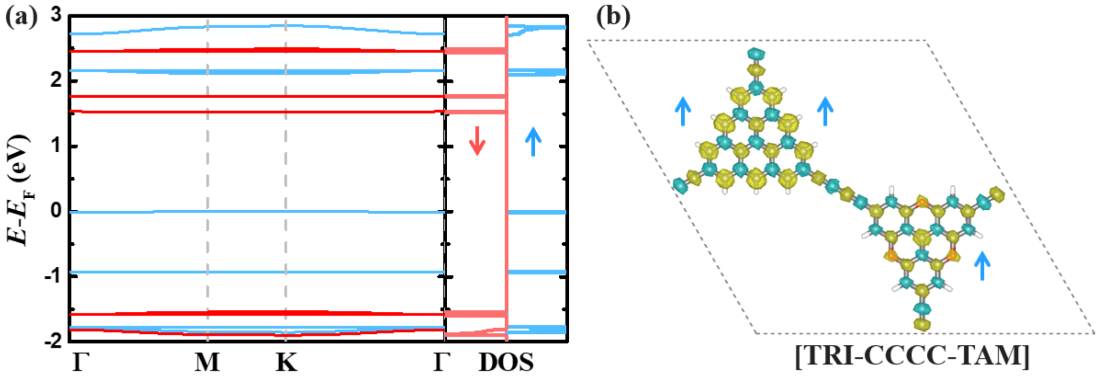

Figure S36. Magnetic properties of [TRI-CCCC-TAM] for (a) spin-polarized band structure and (b) spin density calculated at the PBE0 level.

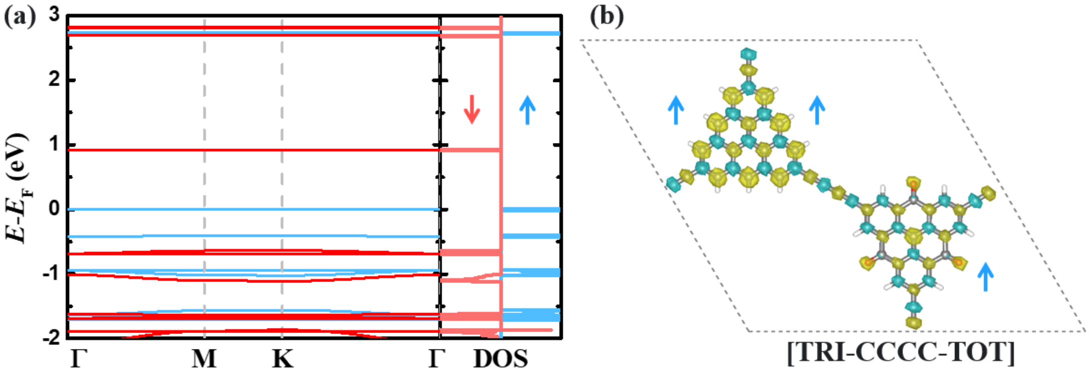

Figure S37. Magnetic properties of [TRI-CCCC-TOT] for (a) spin-polarized band structure and (b) spin density calculated at the PBE0 level.

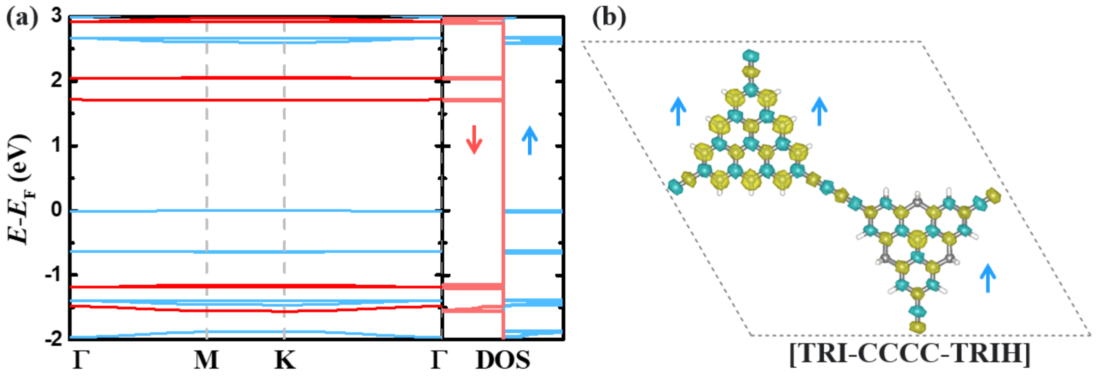

Figure S38. Magnetic properties of [TRI-CCCC-TRIH] for (a) spin-polarized band structure and (b) spin density calculated at the PBE0 level.

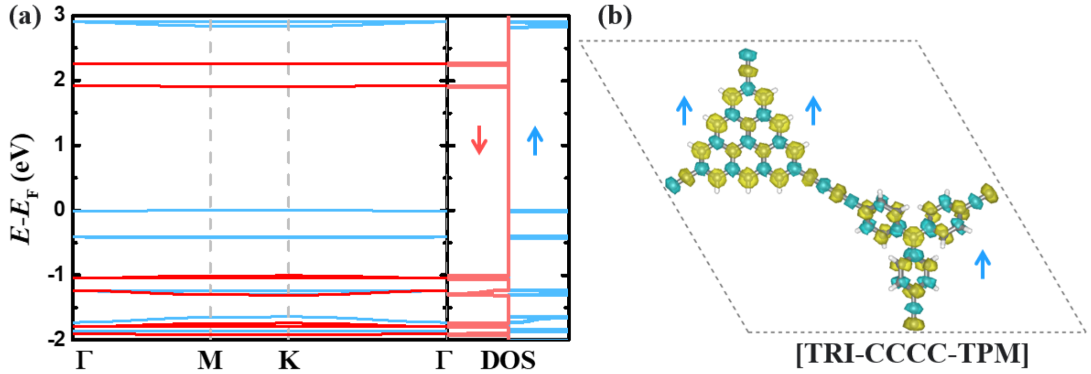

Figure S39. Magnetic properties of [TRI-CCCC-TPM] for (a) spin-polarized band structure and (b) spin density calculated at the PBE0 level.

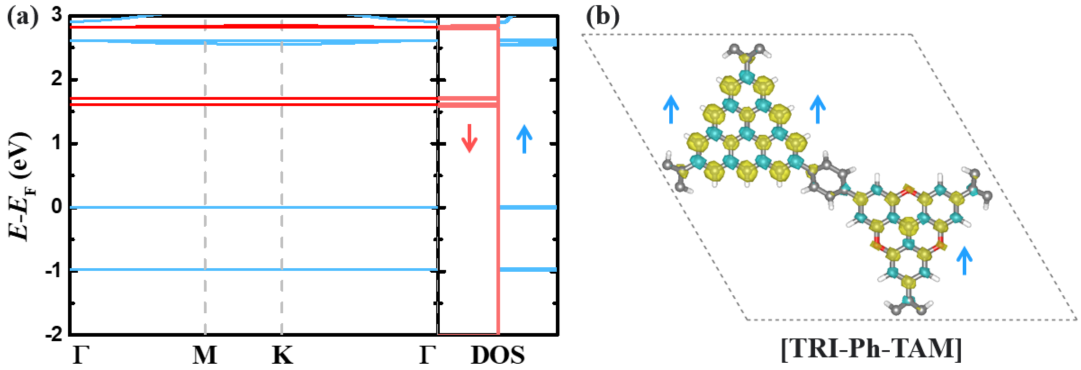

Figure S40. Magnetic properties of [TRI-Ph-TAM] for (a) spin-polarized band structure and (b) spin density calculated at the PBE0 level.

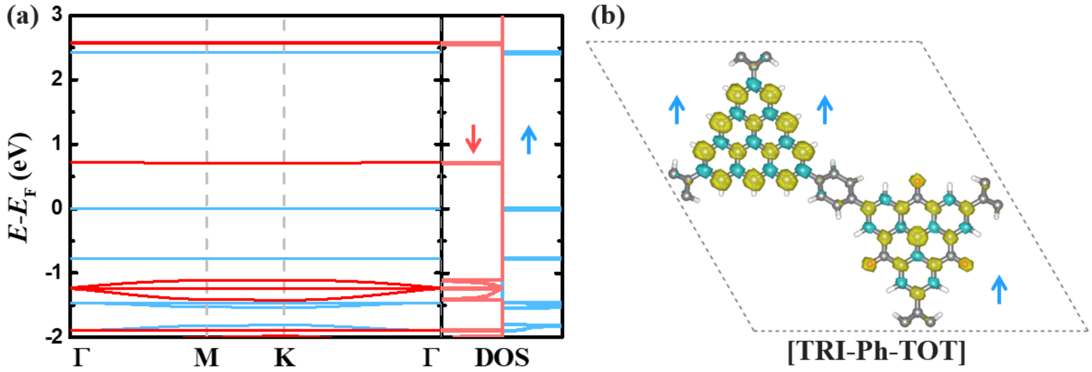

Figure S41. Magnetic properties of [TRI-Ph-TOT] for (a) spin-polarized band structure and (b) spin density calculated at the PBE0 level.

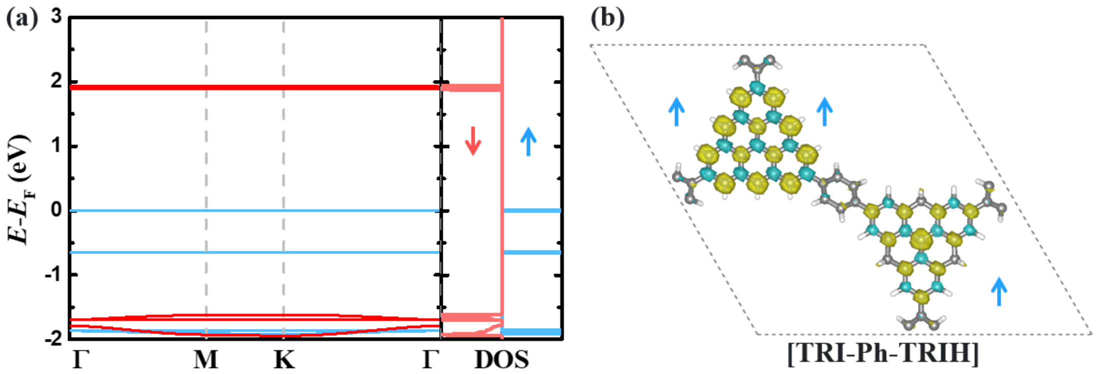

Figure S42. Magnetic properties of [TRI-Ph-TRIH] for (a) spin-polarized band structure and (b) spin density calculated at the PBE0 level.

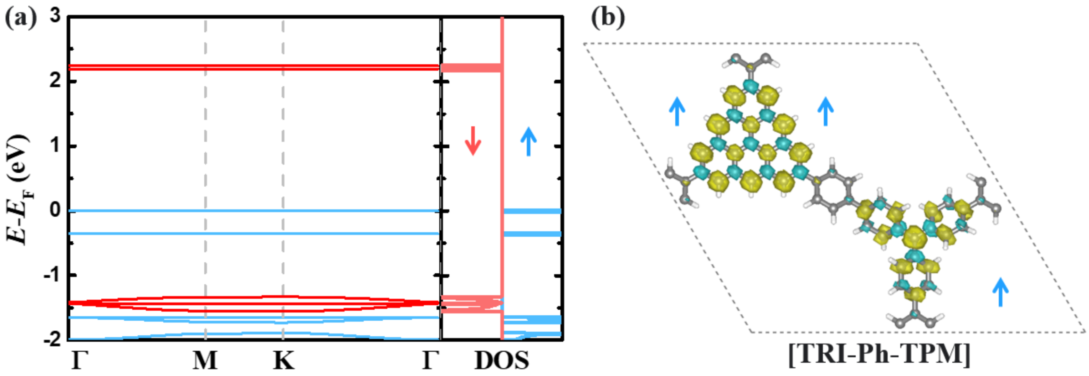

Figure S43. Magnetic properties of [TRI-Ph-TPM] for (a) spin-polarized band structure and (b) spin density calculated at the PBE0 level.

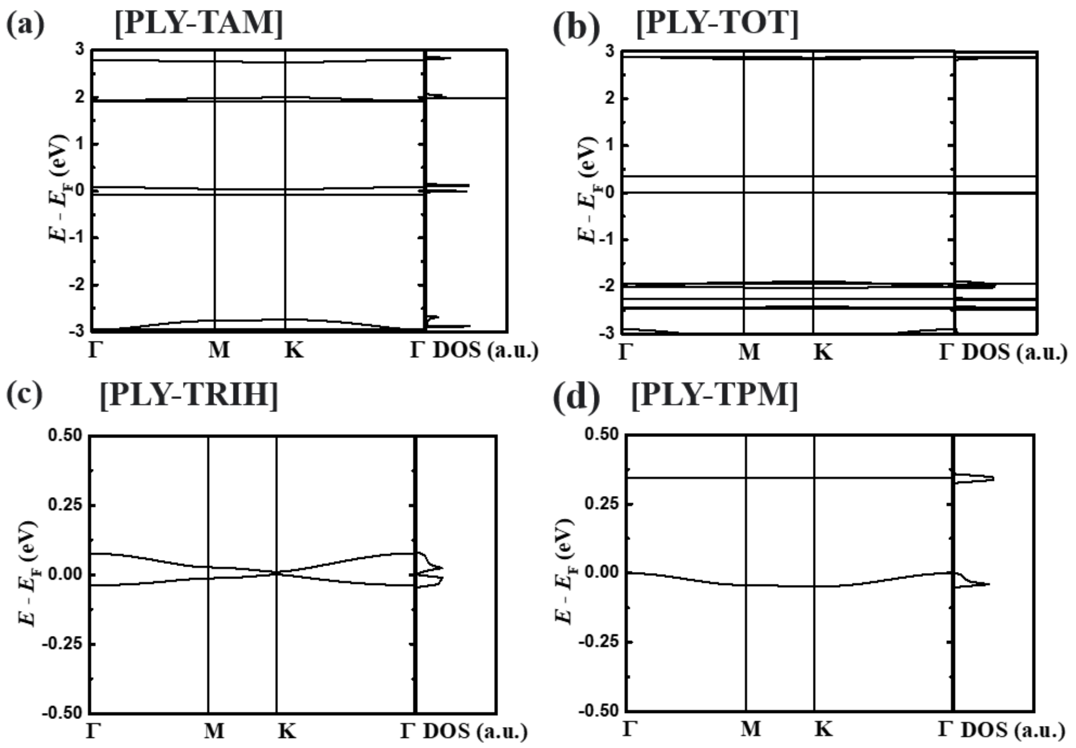

Figure S44. Band structures and density of states (DOS) of the diamagnetic states for (a) [PLY-TAM], (b) [PLY-TOT], (c) [PLY-TRIH], and (d) [PLY-TPM].

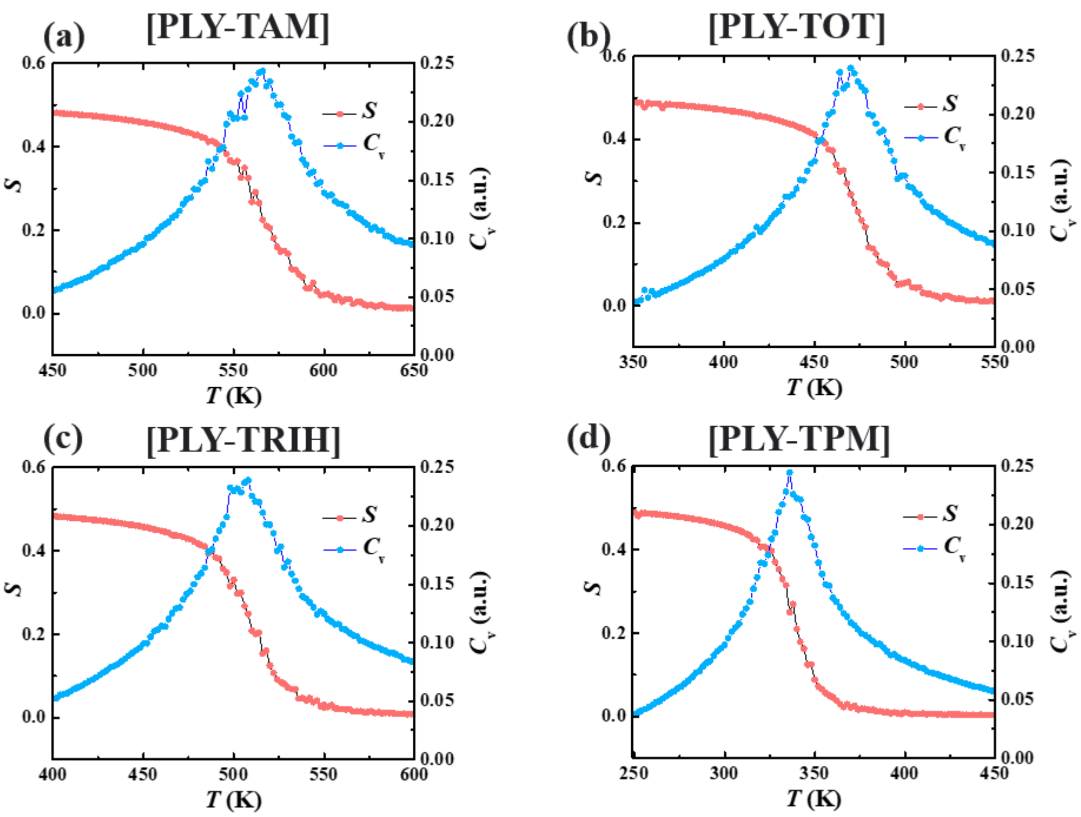

Figure S45. Monte Carlo simulations for (a) [PLY-TAM], (b) [PLY-TOT], (c) [PLY-TRIH], and (d) [PLY-TPM]. Normalized spin (S) per site and electronic heat capacity (C_v) are shown. a.u.: arbitrary units.

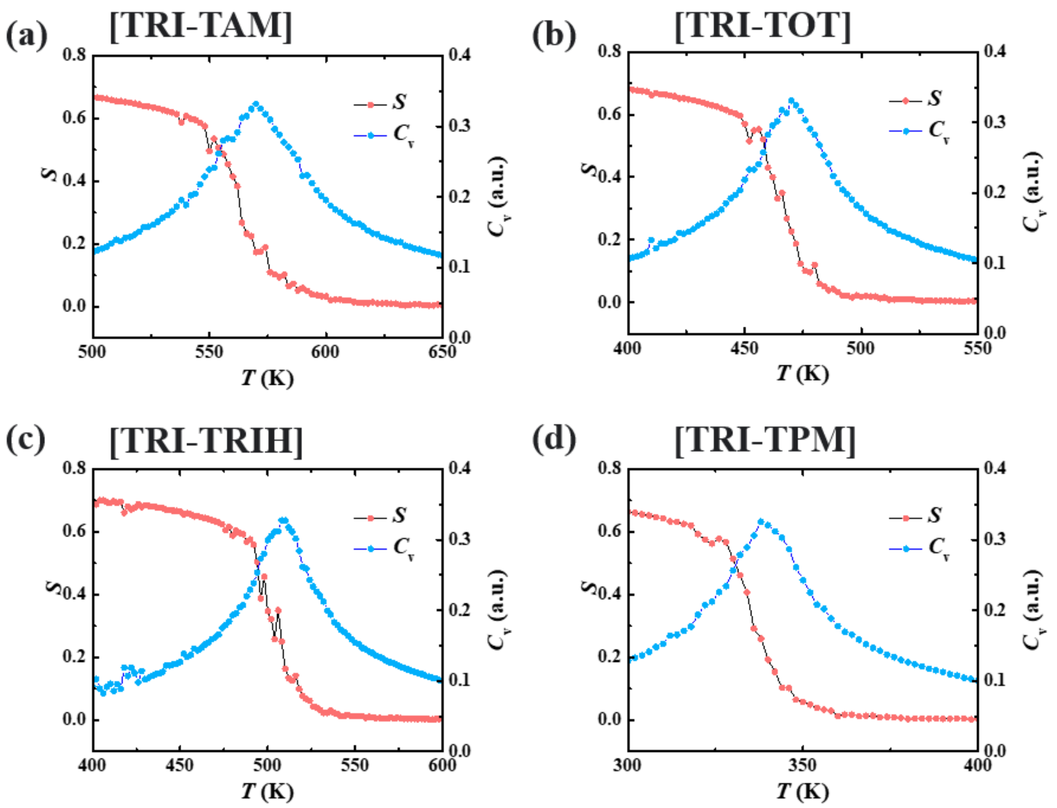

Figure S46. Monte Carlo simulations for (a) [TRI-TAM], (b) [TRI-TOT], (c) [TRI-TRIH], and (d) [TRI-TPM]. Normalized spin (*S*) per site and electronic heat capacity (C_v) are shown. a.u.: arbitrary units.

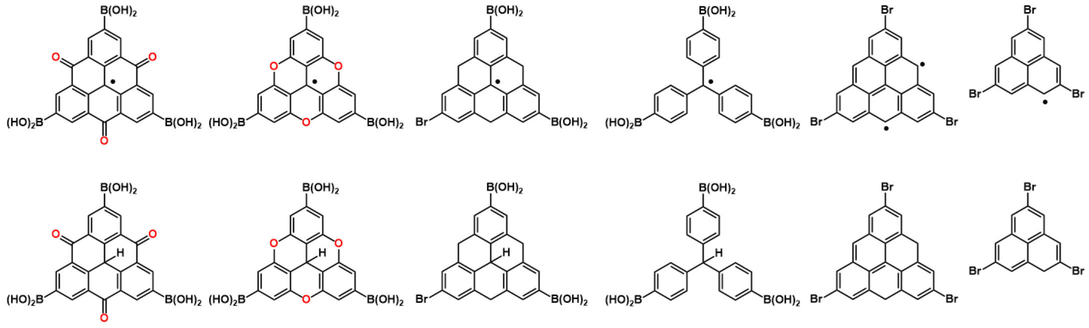

Figure S47. Radicals and diamagnetic precursors for realizing binary 2D frameworks via Suzuki-Miyaura coupling reaction.

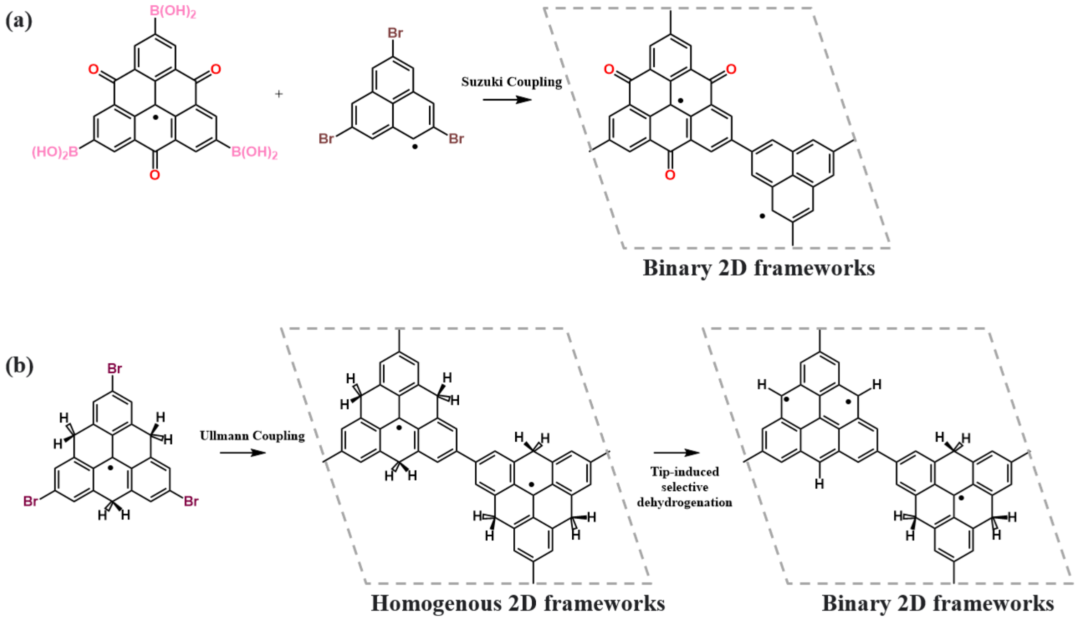

Figure S48. Proposed synthetic routes toward binary 2D covalent frameworks. (a) Suzuki-Miyaura cross-coupling between boronate- and bromide-functionalized molecular precursors. (b) Tip-induced dehydrogenation of a homogeneous 2D framework obtained via Ullmann polymerization.

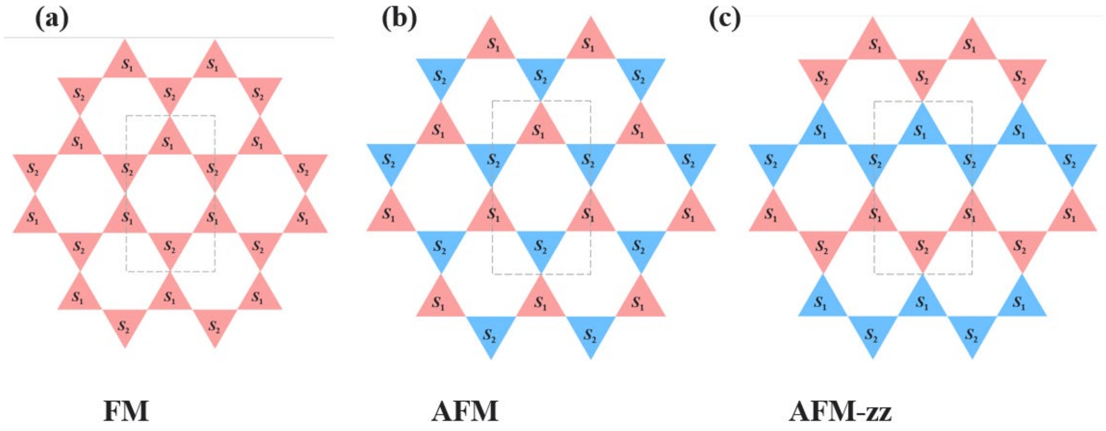

Figure S49. Illustration of the spin arrangements for the FM, AFM and zigzag-type AFM (AFM-zz) configurations in a $\sqrt{2} \times \sqrt{2}$ supercell, where red and blue represent spin-up and spin-down units. S_1 and S_2 are the spin quantum number of each spin center.

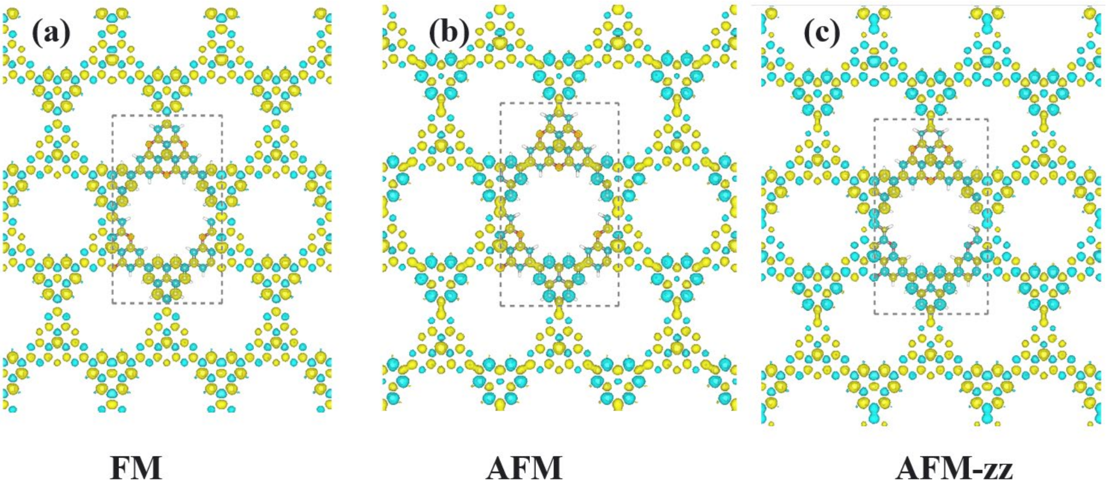

Figure S50. Spin density distribution of [PLY-TAM] for FM, AFM, and zigzag-type AFM (AFM-zz) spin configurations in a $\sqrt{2} \times \sqrt{2}$ supercell.

Table S1. Magnetic coupling for nearest-neighbor (J_1) and next-nearest-neighbor (J_2) spin pairs in representative ferromagnetic 2D frameworks.

	J_1 (meV)	J_2 (meV)	$\|J_1\|/\|J_2\|$
[PLY-TAM]	127	-0.1	1009
[PLY-TOT]	106	-0.1	1018
[PLY-TRIH]	115	0.3	359
[PLY-TPM]	76	0.1	834
[TRI-TAM]	74	-0.7	100
[TRI-TOT]	61	-0.5	120
[TRI-TRIH]	66	-0.4	165
[TRI-TPM]	44	-0.2	265

Table S2. Frontier orbital energies (SOMO and LUMO) calculated at PBE0/def2-TZVP level.

	E (SOMO) (eV)	*E* (LUMO) (eV)
TRI	-5.03	-2.56
PLY	-4.92	-2.59
TAM	-4.15	-2.11
TOT	-6.27	-4.33
TRIH	-4.44	-2.28
TPM	-4.85	-2.40

Table S3. Magnetic couplings *J* calculated at the PBE0 and HSE06 levels.

J (meV)	PBE0	HSE06
[PLY-TAM]	127	122
[PLY-TOT]	106	100
[PLY-TRIH]	115	110
[PLY-TPM]	76	74
[TRI-TAM]	74	71
[TRI-TOT]	61	56
[TRI-TRIH]	66	64
[TRI-TPM]	44	43

References

(1) Kresse, G.; Furthmüller, J. Efficient iterative schemes for ab initio total-energy calculations using a plane-wave basis set. *Phys. Rev. B* **1996**, *54* (16), 11169.

(2) Blöchl, P. E. Projector augmented-wave method. *Phys. Rev. B* **1994**, *50* (24), 17953.

(3) Perdew, J. P.; Burke, K.; Ernzerhof, M. Generalized gradient approximation made simple. *Phys. Rev. Lett.* **1996**, *77* (18), 3865.

(4) Grimme, S.; Antony, J.; Ehrlich, S.; Krieg, H. A consistent and accurate ab initio parametrization of density functional dispersion correction (DFT-D) for the 94 elements H-Pu. *J. Chem. Phys.* **2010**, *132* (15), 154104.

(5) Dovesi, R.; Erba, A.; Orlando, R.; Zicovich-Wilson, C. M.; Civalleri, B.; Maschio, L.; Rérat, M.; Casassa, S.; Baima, J.; Salustro, S. Quantum-mechanical condensed matter simulations with CRYSTAL. *Wiley Interdiscip. Rev. Comput. Mol. Sci.* **2018**, *8* (4), e1360.

(6) Yu, H.; Sun, J.; Heine, T. Predicting magnetic coupling and spin-polarization energy in triangulene analogues. *Journal of chemical theory and computation* **2023**, *19* (12), 3486–3497.

(7) Noodleman, L.; Davidson, E. R. Ligand spin polarization and antiferromagnetic coupling in transition metal dimers. *Chemical Physics* **1986**, *109* (1), 131–143.

(8) Noodleman, L. Valence bond description of antiferromagnetic coupling in transition metal dimers. *J. Chem. Phys.* **1981**, *74* (10), 5737–5743.

(9) Neese, F. Definition of corresponding orbitals and the diradical character in broken symmetry DFT calculations on spin coupled systems. *Journal of Physics and Chemistry of Solids* **2004**, *65* (4), 781–785.

(10) Cramer, C. J.; Truhlar, D. G. Density functional theory for transition metals and transition metal chemistry. *Physical chemistry chemical physics : PCCP* **2009**, *11* (46), 10757–10816.

(11) Lu, T.; Chen, F. Multiwfn: A multifunctional wavefunction analyzer. *Journal of computational chemistry* **2012**, *33* (5), 580–592.

(12) Frisch, M. J.; Trucks, G. W.; Schlegel, H. B.; Scuseria, G. E.; Robb, M. A.; Cheeseman, J. R.; Scalmani, G.; Barone, V.; Petersson, G. A.; Nakatsuji, H.; Li, X.; Caricato, M.; Marenich, A. V.; Bloino, J.; Janesko, B. G.; Gomperts, R.; Mennucci, B.; Hratchian, H. P.; Ortiz, J. V.; Izmaylov, A. F.; Sonnenberg, J. L.; Williams; Ding, F.; Lipparini, F.; Egidi, F.; Goings, J.; Peng,

B.; Petrone, A.; Henderson, T.; Ranasinghe, D.; Zakrzewski, V. G.; Gao, J.; Rega, N.; Zheng, G.; Liang, W.; Hada, M.; Ehara, M.; Toyota, K.; Fukuda, R.; Hasegawa, J.; Ishida, M.; Nakajima, T.; Honda, Y.; Kitao, O.; Nakai, H.; Vreven, T.; Throssell, K.; Montgomery Jr., J. A.; Peralta, J. E.; Ogliaro, F.; Bearpark, M. J.; Heyd, J. J.; Brothers, E. N.; Kudin, K. N.; Staroverov, V. N.; Keith, T. A.; Kobayashi, R.; Normand, J.; Raghavachari, K.; Rendell, A. P.; Burant, J. C.; Iyengar, S. S.; Tomasi, J.; Cossi, M.; Millam, J. M.; Klene, M.; Adamo, C.; Cammi, R.; Ochterski, J. W.; Martin, R. L.; Morokuma, K.; Farkas, O.; Foresman, J. B.; Fox, D. J. *Gaussian 16 Rev. C.01*, 2016.

(13) Adamo, C.; Barone, V. Toward reliable density functional methods without adjustable parameters: The PBE0 model. *J. Chem. Phys.* **1999**, *110* (13), 6158–6170.

(14) Yu, H.; Heine, T. *Ferromagnetic organic 2D crystals*; Zenodo, 2025.

(15) Kühne, T. D.; Iannuzzi, M.; Del Ben, M.; Rybkin, V. V.; Seewald, P.; Stein, F.; Laino, T.; Khaliullin, R. Z.; Schütt, O.; Schiffmann, F.; Golze, D.; Wilhelm, J.; Chulkov, S.; Bani-Hashemian, M. H.; Weber, V.; Borštnik, U.; Taillefumier, M.; Jakobovits, A. S.; Lazzaro, A.; Pabst, H.; Müller, T.; Schade, R.; Guidon, M.; Andermatt, S.; Holmberg, N.; Schenter, G. K.; Hehn, A.; Bussy, A.; Belleflamme, F.; Tabacchi, G.; Glöß, A.; Lass, M.; Bethune, I.; Mundy, C. J.; Plessl, C.; Watkins, M.; VandeVondele, J.; Krack, M.; Hutter, J. CP2K: An electronic structure and molecular dynamics software package - Quickstep: Efficient and accurate electronic structure calculations. *J. Chem. Phys.* **2020**, *152* (19), 194103.

(16) Goedecker, S.; Teter, M.; Hutter, J. Separable dual-space Gaussian pseudopotentials. *Physical review. B, Condensed matter* **1996**, *54* (3), 1703–1710.

(17) Hartwigsen, C.; Goedecker, S.; Hutter, J. Relativistic separable dual-space Gaussian pseudopotentials from H to Rn. *Phys. Rev. B* **1998**, *58* (7), 3641–3662.

(18) VandeVondele, J.; Hutter, J. An efficient orbital transformation method for electronic structure calculations. *J. Chem. Phys.* **2003**, *118* (10), 4365–4369.

(19) Mermin, N. D.; Wagner, H. Absence of ferromagnetism or antiferromagnetism in one-or two-dimensional isotropic Heisenberg models. *Phys. Rev. Lett.* **1966**, *17* (22), 1133.

(20) Bramwell, S. T.; Holdsworth, P. C. W. Universality in two-dimensional magnetic systems. *Journal of Applied Physics* **1993**, *73* (10), 6096–6098.

(21) Ortiz, R.; Catarina, G.; Fernández-Rossier, J. Theory of triangulene two-dimensional crystals. *2D Materials* **2022**, *10* (1), 15015.

(22) Mishra, S.; Catarina, G.; Wu, F.; Ortiz, R.; Jacob, D.; Eimre, K.; Ma, J.; Pignedoli, C. A.; Feng, X.; Ruffieux, P.; Fernández-Rossier, J.; Fasel, R. Observation of fractional edge excitations in nanographene spin chains. *Nature* **2021**, *598* (7880), 287–292.

(23) Pavliček, N.; Mistry, A.; Majzik, Z.; Moll, N.; Meyer, G.; Fox, D. J.; Gross, L. Synthesis and characterization of triangulene. *Nat. Nanotechnol.* **2017**, *12* (4), 308–311.

(24) Jiang, X.; Liu, Q.; Xing, J.; Liu, N.; Guo, Y.; Liu, Z.; Zhao, J. Recent progress on 2D magnets: Fundamental mechanism, structural design and modification. *Appl. Phys. Rev.* **2021**, *8* (3).

(25) Huang, B.; McGuire, M. A.; May, A. F.; Di Xiao; Jarillo-Herrero, P.; Xu, X. Emergent phenomena and proximity effects in two-dimensional magnets and heterostructures. *Nat. Mater.* **2020**, *19* (12), 1276–1289.

(26) Coey, J. M. D. *Magnetism and magnetic materials*; Cambridge university press, 2010.

[PLY-TAM]

a = 17.20, 17.20, c = 29.40, alpha = 90.0, beta = 90.0, gamma = 120.0

C	0.66721432	0.47445797	0.50000000
C	0.33310291	0.52312297	0.50000000
C	0.52555072	0.19277118	0.50000000
C	0.47688439	0.81000029	0.50000000
C	0.80720863	0.33277301	0.50000000
C	0.19002041	0.66686667	0.50000000
C	0.47687703	0.66689709	0.50000000
C	0.52554203	0.33278568	0.50000000
C	0.18999971	0.52311561	0.50000000
C	0.80722882	0.47444927	0.50000000
C	0.33313333	0.80997959	0.50000000
C	0.66722699	0.19279137	0.50000000
C	0.62126088	0.52117443	0.50000000
C	0.38228702	0.47751178	0.50000000
C	0.47884045	0.10009104	0.50000000
C	0.52250562	0.90480607	0.50000000
C	0.89987818	0.37872030	0.50000000
C	0.09521226	0.61768550	0.50000000
C	0.52248822	0.61771298	0.50000000
C	0.47882557	0.37873912	0.50000000
C	0.09519393	0.47749437	0.50000000
C	0.89990896	0.52115955	0.50000000
C	0.38231450	0.90478774	0.50000000
C	0.62127969	0.10012182	0.50000000
C	0.61980700	0.38019300	0.50000000
C	0.38092030	0.61907970	0.50000000
C	0.61981422	0.23963219	0.50000000
C	0.38093161	0.76184099	0.50000000
C	0.76036781	0.38018578	0.50000000
C	0.23815901	0.61906839	0.50000000
C	0.52613681	0.47386319	0.50000000
C	0.47666748	0.52333252	0.50000000
C	0.52616587	0.05231126	0.50000000
C	0.47669519	0.95336185	0.50000000
C	0.94768874	0.47383413	0.50000000
C	0.04663814	0.52330481	0.50000000
C	0.23843754	0.47689993	0.50000000
C	0.52310007	0.76156246	0.50000000
C	0.23846677	0.76153323	0.50000000
C	0.66665761	0.33334239	0.50000000
C	0.33333870	0.66666130	0.50000000
O	0.76064266	0.52128440	0.50000000

O 0.47871560 0.23935734 0.50000000
O 0.76064455 0.23935545 0.50000000
H 0.65996226 0.59415738 0.50000000
H 0.34369204 0.40437981 0.50000000
H 0.40585699 0.06579773 0.50000000
H 0.59563365 0.93932937 0.50000000
H 0.93417336 0.34004288 0.50000000
H 0.06069755 0.65629333 0.50000000
H 0.59562019 0.65630796 0.50000000
H 0.40584262 0.34003774 0.50000000
H 0.06067063 0.40436635 0.50000000
H 0.93420227 0.59414300 0.50000000
H 0.34370667 0.93930245 0.50000000
H 0.65995712 0.06582663 0.50000000
H 0.59652684 0.79826895 0.50000000
H 0.20175649 0.79824351 0.50000000
H 0.20173105 0.40347316 0.50000000

[PLY-TOT]
a = 14.87, 14.87, c = 29.40, alpha = 90.0, beta = 90.0, gamma = 120.0
C 0.62475421 0.55203091 0.50000000
C 0.23791833 0.60951466 0.50000000
C 0.46067056 0.21917181 0.50000000
C 0.40328882 0.94031211 0.50000000
C 0.79352144 0.38800325 0.50000000
C 0.40318895 0.77480069 0.50000000
C 0.46068738 0.38797187 0.50000000
C 0.07240120 0.60939750 0.50000000
C 0.79356353 0.55205338 0.50000000
C 0.62469888 0.21918859 0.50000000
C 0.56961169 0.60474771 0.50000000
C 0.29428959 0.55634156 0.50000000
C 0.40793861 0.11129191 0.50000000
C 0.90139735 0.44314871 0.50000000
C 0.45637782 0.71844597 0.50000000
C 0.40797564 0.44311963 0.50000000
C 0.90144125 0.60477410 0.50000000
C 0.56954731 0.11131416 0.50000000
C 0.57107615 0.44164413 0.50000000
C 0.29301215 0.71969097 0.50000000
C 0.57105449 0.27590137 0.50000000
C 0.29427246 0.88431376 0.50000000
C 0.73682511 0.44166627 0.50000000
C 0.12838427 0.71841089 0.50000000

C 0.46056188 0.55216606 0.50000000
C 0.40331446 0.60941846 0.50000000
C 0.46050582 0.05482714 0.50000000
C 0.95788742 0.55218881 0.50000000
C 0.73957648 0.61286038 0.50000000
C 0.12835535 0.55633764 0.50000000
C 0.39985519 0.27315249 0.50000000
C 0.45635623 0.88436416 0.50000000
C 0.73951115 0.27319256 0.50000000
C 0.23793391 0.77476497 0.50000000
C 0.62632566 0.38640631 0.50000000
H 0.61638173 0.68927900 0.50000000
H 0.25000035 0.47169672 0.50000000
H 0.32340706 0.07351081 0.50000000
H 0.93914306 0.39637570 0.50000000
H 0.54102394 0.76274803 0.50000000
H 0.32344372 0.39635248 0.50000000
H 0.93923169 0.68930499 0.50000000
H 0.61632151 0.07356845 0.50000000
H 0.54100399 0.92474249 0.50000000
H 0.08798527 0.47169104 0.50000000
H 0.24995849 0.92463315 0.50000000
H 0.08805584 0.76271499 0.50000000
O 0.30327174 0.22484877 0.50000000
O 0.78779340 0.22490521 0.50000000
O 0.78787745 0.70944322 0.50000000

[PLY-TRIH]
a = 14.92, 14.92, c = 29.40, alpha = 90.0, beta = 90.0, gamma = 120.0
C 0.62356245 0.55158807 0.49995907
C 0.23779562 0.60951224 0.50000479
C 0.46076237 0.21825272 0.50001455
C 0.40301030 0.93979942 0.50000598
C 0.79409017 0.38883037 0.49993035
C 0.40284257 0.77450627 0.50000390
C 0.46071153 0.38869085 0.50000647
C 0.07255072 0.60946319 0.50000406
C 0.79407182 0.55168874 0.50005707
C 0.62363913 0.21829900 0.49998074
C 0.56866541 0.60393059 0.49988489
C 0.29418314 0.55663037 0.49998590
C 0.40840899 0.11099543 0.49993594
C 0.90134217 0.44371585 0.49996948
C 0.45568613 0.71808939 0.49997948

C 0.40831888 0.44354541 0.49998327
C 0.90132873 0.60407646 0.50002797
C 0.56878977 0.11103519 0.50006838
C 0.57050027 0.44180487 0.49997539
C 0.29283436 0.71952146 0.50000659
C 0.57054650 0.27497908 0.49999692
C 0.29427939 0.88381400 0.49999937
C 0.73738881 0.44189665 0.49999464
C 0.12859219 0.71819260 0.50000090
C 0.45992523 0.55229764 0.49991769
C 0.40291450 0.60935585 0.49995458
C 0.46004235 0.05388554 0.50000419
C 0.95846701 0.55246581 0.50000199
C 0.73973113 0.61351955 0.50026108
C 0.12851644 0.55669369 0.50000544
C 0.39885825 0.27251213 0.50026744
C 0.45572680 0.88378866 0.50000937
C 0.73981846 0.27264581 0.49971026
C 0.23786164 0.77454557 0.50000185
C 0.62614630 0.38622526 0.49999941
H 0.61325272 0.68833937 0.49986864
H 0.24990815 0.47229502 0.49998867
H 0.32399993 0.07117364 0.49985659
H 0.94113336 0.39910361 0.49993880
H 0.54001927 0.76235991 0.49998620
H 0.32390894 0.39890090 0.50007387
H 0.94112504 0.68848986 0.50005953
H 0.61341569 0.07125453 0.50014808
H 0.54006249 0.92384982 0.50000095
H 0.34575730 0.24590333 0.47077248
H 0.76639839 0.24617540 0.52922169
H 0.08842946 0.47235923 0.50000060
H 0.76632221 0.66661947 0.47076608
H 0.25002643 0.92389124 0.49999713
H 0.08856965 0.76249995 0.49999715
H 0.76532119 0.66493315 0.53033869
H 0.34745315 0.24679942 0.53034830
H 0.76553693 0.24690847 0.46964723

[PLY-TPM]
a = 14.88, 14.88, c = 29.40, alpha = 90.0, beta = 90.0, gamma = 120.0
H 0.58568400 0.66405673 0.45786214
H 0.25130258 0.47113201 0.49888019
H 0.34826807 0.06786327 0.45787949

H	0.94450699	0.42671187	0.45794017
H	0.54123635	0.76104377	0.50101435
H	0.34824277	0.42673638	0.54205562
H	0.94453775	0.66407649	0.54213871
H	0.58564842	0.06781872	0.54204087
H	0.54123299	0.92646389	0.49883592
H	0.08591336	0.47113785	0.50105723
H	0.25132315	0.92641837	0.50106908
H	0.08595025	0.76104577	0.49898202
H	0.68209151	0.56946900	0.45850843
H	0.75352508	0.56945483	0.54157203
H	0.44282719	0.25887324	0.45852228
H	0.75349971	0.33036072	0.45849148
H	0.68200918	0.25882043	0.54150959
H	0.44289632	0.33039282	0.54156190
C	0.60941208	0.53869652	0.47732693
C	0.23750567	0.60889221	0.49995727
C	0.47362921	0.21698468	0.47732917
C	0.40280948	0.93950905	0.49994491
C	0.79539100	0.40300045	0.47734011
C	0.40348303	0.77486638	0.49993517
C	0.47367800	0.40302638	0.52270671
C	0.07285205	0.60955056	0.50002521
C	0.79539972	0.53867703	0.52273776
C	0.60934396	0.21694278	0.52267815
C	0.55533015	0.59198444	0.47716383
C	0.29403441	0.55583007	0.49999754
C	0.42035905	0.10961106	0.47716796
C	0.90276638	0.45706755	0.47722150
C	0.45653946	0.71832766	0.49987524
C	0.42035526	0.45707387	0.52278494
C	0.90277497	0.59197663	0.52285674
C	0.55529044	0.10956975	0.52277001
C	0.57002277	0.44237899	0.50002999
C	0.29283202	0.71953865	0.49995614
C	0.56997117	0.27392840	0.50001832
C	0.29404077	0.88444792	0.49992094
C	0.73843522	0.44235567	0.50003990
C	0.12791459	0.71832048	0.50008024
C	0.45975382	0.55261722	0.49995873
C	0.40280855	0.60955592	0.49993168
C	0.45973559	0.05340396	0.49995510
C	0.95895690	0.55261709	0.50003887
C	0.12790083	0.55583572	0.49993190

C 0.45653389 0.88447057 0.49998280
C 0.23751058 0.77484882 0.49998626
C 0.62614333 0.38621733 0.50003847

[TRI-TAM]
a = 17.20, 17.20, c = 29.40, alpha = 90.0, beta = 90.0, gamma = 120.0
C 0.66721432 0.47445797 0.50000000
C 0.33310291 0.52312297 0.50000000
C 0.52555072 0.19277118 0.50000000
C 0.47688439 0.81000029 0.50000000
C 0.80720863 0.33277301 0.50000000
C 0.19002041 0.66686667 0.50000000
C 0.47687703 0.66689709 0.50000000
C 0.52554203 0.33278568 0.50000000
C 0.18999971 0.52311561 0.50000000
C 0.80722882 0.47444927 0.50000000
C 0.33313333 0.80997959 0.50000000
C 0.66722699 0.19279137 0.50000000
C 0.62126088 0.52117443 0.50000000
C 0.38228702 0.47751178 0.50000000
C 0.47884045 0.10009104 0.50000000
C 0.52250562 0.90480607 0.50000000
C 0.89987818 0.37872030 0.50000000
C 0.09521226 0.61768550 0.50000000
C 0.52248822 0.61771298 0.50000000
C 0.47882557 0.37873912 0.50000000
C 0.09519393 0.47749437 0.50000000
C 0.89990896 0.52115955 0.50000000
C 0.38231450 0.90478774 0.50000000
C 0.62127969 0.10012182 0.50000000
C 0.61980700 0.38019300 0.50000000
C 0.38092030 0.61907970 0.50000000
C 0.61981422 0.23963219 0.50000000
C 0.38093161 0.76184099 0.50000000
C 0.76036781 0.38018578 0.50000000
C 0.23815901 0.61906839 0.50000000
C 0.52613681 0.47386319 0.50000000
C 0.47666748 0.52333252 0.50000000
C 0.52616587 0.05231126 0.50000000
C 0.47669519 0.95336185 0.50000000
C 0.94768874 0.47383413 0.50000000
C 0.04663814 0.52330481 0.50000000
C 0.23843754 0.47689993 0.50000000
C 0.52310007 0.76156246 0.50000000

C 0.23846677 0.76153323 0.50000000
C 0.66665761 0.33334239 0.50000000
C 0.33333870 0.66666130 0.50000000
O 0.76064266 0.52128440 0.50000000
O 0.47871560 0.23935734 0.50000000
O 0.76064455 0.23935545 0.50000000
H 0.65996226 0.59415738 0.50000000
H 0.34369204 0.40437981 0.50000000
H 0.40585699 0.06579773 0.50000000
H 0.59563365 0.93932937 0.50000000
H 0.93417336 0.34004288 0.50000000
H 0.06069755 0.65629333 0.50000000
H 0.59562019 0.65630796 0.50000000
H 0.40584262 0.34003774 0.50000000
H 0.06067063 0.40436635 0.50000000
H 0.93420227 0.59414300 0.50000000
H 0.34370667 0.93930245 0.50000000
H 0.65995712 0.06582663 0.50000000
H 0.59652684 0.79826895 0.50000000
H 0.20175649 0.79824351 0.50000000
H 0.20173105 0.40347316 0.50000000

[TRI-TOT]
a = 17.33, 17.33, c = 29.40, alpha = 90.0, beta = 90.0, gamma = 120.0
C 0.66535302 0.47545530 0.50000000
C 0.33306483 0.52430926 0.50000000
C 0.52455989 0.18989841 0.50000000
C 0.47570394 0.80875174 0.50000000
C 0.81011204 0.33466788 0.50000000
C 0.19126792 0.66693961 0.50000000
C 0.47570779 0.66695688 0.50000000
C 0.52457910 0.33468603 0.50000000
C 0.19126636 0.52430674 0.50000000
C 0.81012731 0.47544656 0.50000000
C 0.33306268 0.80873785 0.50000000
C 0.66532837 0.18990349 0.50000000
C 0.61801034 0.52065533 0.50000000
C 0.38181564 0.47902722 0.50000000
C 0.47936409 0.09734961 0.50000000
C 0.52098630 0.90278819 0.50000000
C 0.90265813 0.38201439 0.50000000
C 0.09723384 0.61818966 0.50000000
C 0.52099641 0.61821372 0.50000000
C 0.47938805 0.38203644 0.50000000

C	0.09723070	0.47901962	0.50000000
C	0.90267290	0.52063293	0.50000000
C	0.38180822	0.90277203	0.50000000
C	0.61797703	0.09735785	0.50000000
C	0.61929989	0.38073616	0.50000000
C	0.38052207	0.61949512	0.50000000
C	0.61928458	0.23857759	0.50000000
C	0.38051869	0.76101422	0.50000000
C	0.76145082	0.38072447	0.50000000
C	0.23899822	0.61949090	0.50000000
C	0.52444545	0.47559998	0.50000000
C	0.47533246	0.52469834	0.50000000
C	0.52441708	0.04884379	0.50000000
C	0.47531957	0.95063113	0.50000000
C	0.95117207	0.47557495	0.50000000
C	0.04938280	0.52467898	0.50000000
C	0.76383423	0.52762983	0.50000000
C	0.23917366	0.47832970	0.50000000
C	0.47238989	0.23620326	0.50000000
C	0.52168582	0.76084778	0.50000000
C	0.76380251	0.23619241	0.50000000
C	0.23917519	0.76082596	0.50000000
C	0.66667868	0.33334688	0.50000000
C	0.33334680	0.66666599	0.50000000
H	0.65807282	0.59316829	0.50000000
H	0.34394706	0.40642894	0.50000000
H	0.40685128	0.06488295	0.50000000
H	0.59358474	0.93753140	0.50000000
H	0.93510605	0.34195444	0.50000000
H	0.06249880	0.65604811	0.50000000
H	0.59359503	0.65608075	0.50000000
H	0.40687604	0.34198401	0.50000000
H	0.06248635	0.40642128	0.50000000
H	0.93514133	0.59314436	0.50000000
H	0.34393942	0.93749664	0.50000000
H	0.65803700	0.06491028	0.50000000
H	0.59453389	0.79727614	0.50000000
H	0.20274952	0.79724309	0.50000000
H	0.20273933	0.40548185	0.50000000
O	0.38948807	0.19475904	0.50000000
O	0.80524098	0.19473216	0.50000000
O	0.80528693	0.61053210	0.50000000

[TRI-TRIH]

a = 17.38, 17.38, c = 29.40, alpha = 90.0, beta = 90.0, gamma = 120.0

C	0.66471340	0.47550918	0.49999365
C	0.33324662	0.52471157	0.49999951
C	0.52479545	0.18936887	0.49992948
C	0.47546584	0.80862396	0.49999557
C	0.81090248	0.33556597	0.50003058
C	0.19160152	0.66694602	0.50000371
C	0.47549571	0.66702029	0.49999497
C	0.52486809	0.33568137	0.50003494
C	0.19162029	0.52468144	0.49999269
C	0.81095659	0.47542317	0.49998296
C	0.33318989	0.80858950	0.50000042
C	0.66459043	0.18933241	0.50007785
C	0.61764322	0.52047998	0.50002032
C	0.38197731	0.47963100	0.50002416
C	0.47982803	0.09732183	0.49995947
C	0.52056281	0.90244091	0.49999822
C	0.90294133	0.38263166	0.49993852
C	0.09779415	0.61823020	0.50000147
C	0.52063584	0.61835003	0.50001806
C	0.47995240	0.38279242	0.50011550
C	0.09780676	0.47955497	0.49999444
C	0.90299352	0.52034520	0.50006026
C	0.38187809	0.90241142	0.49999845
C	0.61747450	0.09729287	0.50003577
C	0.61911130	0.38126193	0.50002412
C	0.38053607	0.61967884	0.49999469
C	0.61904021	0.23802043	0.50000466
C	0.38050782	0.76095151	0.49999761
C	0.76227235	0.38117999	0.50000675
C	0.23925644	0.61964648	0.49999758
C	0.52428498	0.47614061	0.50008867
C	0.47531529	0.52502100	0.50005176
C	0.52412799	0.04827610	0.49999605
C	0.47520475	0.95041156	0.49999731
C	0.95197938	0.47598187	0.49999694
C	0.04982325	0.52489822	0.49999707
C	0.76442801	0.52860011	0.49973164
C	0.23958582	0.47898646	0.49999364
C	0.47170306	0.23597593	0.49972158
C	0.52117852	0.76068220	0.49999116
C	0.76429031	0.23586137	0.50030560
C	0.23953746	0.76061270	0.50000308
C	0.66680508	0.33348707	0.50000173

C 0.33343570 0.66676051 0.49999542
H 0.65595043 0.59293501 0.49993155
H 0.34406917 0.40724683 0.50001422
H 0.40737696 0.06317168 0.49992960
H 0.59294177 0.93690893 0.49999947
H 0.93708214 0.34432366 0.49986138
H 0.06333224 0.65614437 0.49999717
H 0.59301698 0.65629215 0.50001635
H 0.40750176 0.34451811 0.50013117
H 0.06334842 0.40717320 0.50000142
H 0.93718411 0.59279772 0.50013567
H 0.34394552 0.93685500 0.49999881
H 0.65575088 0.06312029 0.50006553
H 0.59383197 0.79701609 0.49999668
H 0.42614025 0.21315297 0.52922115
H 0.20319332 0.79691902 0.50000015
H 0.78700619 0.21305623 0.47078835
H 0.20325702 0.40632946 0.49999705
H 0.78727335 0.57416837 0.52923063
H 0.78650106 0.57272889 0.46965509
H 0.42757271 0.21400615 0.46964793
H 0.78637561 0.21377461 0.53036271

[TRI-TPM]
a = 17.35, 17.35, c = 29.40, alpha = 90.0, beta = 90.0, gamma = 120.0
H 0.63220553 0.57190291 0.54208606
H 0.34529031 0.40623319 0.50119920
H 0.42831244 0.06032671 0.54203547
H 0.59402133 0.93922348 0.50113111
H 0.93987349 0.36810550 0.54206681
H 0.06105925 0.65492527 0.50104438
H 0.59402206 0.65495151 0.49899877
H 0.42826701 0.36819759 0.45798328
H 0.06106795 0.40622939 0.49897467
H 0.93998022 0.57188967 0.45789595
H 0.34530777 0.93918743 0.49884892
H 0.63208935 0.06028115 0.45791759
H 0.59457476 0.79736379 0.50005820
H 0.20290852 0.79732517 0.50000255
H 0.20291065 0.40569911 0.50007888
H 0.71485566 0.49066606 0.54143953
H 0.50952222 0.22423403 0.54143930
H 0.77596462 0.28548170 0.54151817
H 0.50952797 0.28556267 0.45838439

H	0.77607472	0.49058969	0.45833174
H	0.71472999	0.22419098	0.45840817
C	0.65248259	0.46429807	0.52262729
C	0.33310019	0.52423805	0.49998807
C	0.53590585	0.18826089	0.52261558
C	0.47603632	0.80900304	0.49992785
C	0.81194224	0.34779281	0.52265595
C	0.19126675	0.66715207	0.49990802
C	0.47603728	0.66717355	0.50022674
C	0.53592991	0.34786300	0.47725794
C	0.19126820	0.52423007	0.50019660
C	0.81200252	0.46423681	0.47722285
C	0.33309404	0.80898692	0.50014825
C	0.65239666	0.18823684	0.47725249
C	0.60611102	0.51004271	0.52279927
C	0.38185067	0.47889458	0.50004988
C	0.49017682	0.09613994	0.52275526
C	0.52136252	0.90310180	0.49996720
C	0.90407133	0.39416101	0.52278132
C	0.09716814	0.61837998	0.49993579
C	0.52136606	0.61840883	0.50017746
C	0.49018289	0.39423356	0.47722044
C	0.09717046	0.47888760	0.50012297
C	0.90412785	0.51000162	0.47715878
C	0.38185445	0.90308499	0.50005414
C	0.60603260	0.09611652	0.47717168
C	0.61863230	0.38164216	0.49991526
C	0.38073736	0.61953429	0.50010639
C	0.61857366	0.23710710	0.49991931
C	0.38072938	0.76132581	0.50005585
C	0.76310765	0.38158299	0.49992337
C	0.23893305	0.61952566	0.50006777
C	0.52403162	0.47622865	0.50004987
C	0.47518411	0.52507288	0.50010512
C	0.52401601	0.04793006	0.49997913
C	0.47518264	0.95024476	0.50000287
C	0.95231258	0.47619338	0.49998793
C	0.05000589	0.52504955	0.50001885
C	0.23930835	0.47848776	0.50009139
C	0.52178619	0.76096482	0.50007031
C	0.23930361	0.76094033	0.50001657
C	0.66676538	0.33344343	0.49990885
C	0.33346705	0.66679413	0.50007620

[PLY-CC-TAM]

a = 19.10, 19.09, c = 29.40, alpha = 90.0, beta = 90.0, gamma = 120.0

C	0.57395829	0.50790913	0.50000000
C	0.19623171	0.62709912	0.50000000
C	0.44589545	0.25370745	0.50000000
C	0.32501844	0.88463143	0.50000000
C	0.69983650	0.37956940	0.50000000
C	0.32580992	0.75669741	0.50000000
C	0.44585770	0.37966964	0.50000000
C	0.06853993	0.62817764	0.50000000
C	0.69980037	0.50785000	0.50000000
C	0.57401916	0.25363263	0.50000000
C	0.53299631	0.55040072	0.50000000
C	0.23989610	0.58540686	0.50000000
C	0.40344720	0.17027935	0.50000000
C	0.78320727	0.42052238	0.50000000
C	0.36738849	0.71292833	0.50000000
C	0.40338713	0.42063486	0.50000000
C	0.78316496	0.55031690	0.50000000
C	0.53308556	0.17018339	0.50000000
C	0.53094384	0.42273500	0.50000000
C	0.23943890	0.71355026	0.50000000
C	0.53098106	0.29575663	0.50000000
C	0.24004967	0.84222048	0.50000000
C	0.65777896	0.42267933	0.50000000
C	0.11102926	0.71322692	0.50000000
C	0.44725014	0.50645394	0.50000000
C	0.32487321	0.62788641	0.50000000
C	0.44734776	0.12837889	0.50000000
C	0.82498623	0.50633396	0.50000000
C	0.36750448	0.58536820	0.50000000
C	0.40472553	0.54879226	0.50000000
C	0.36768713	0.96975754	0.50000000
C	0.40484915	0.04353981	0.50000000
C	0.98347752	0.58565445	0.50000000
C	0.90975364	0.54875721	0.50000000
C	0.11095354	0.58554347	0.50000000
C	0.36746259	0.84204480	0.50000000
C	0.57323420	0.38039064	0.50000000
C	0.19630926	0.75687050	0.50000000
O	0.65782542	0.54980151	0.50000000
O	0.40399786	0.29573480	0.50000000
O	0.65787737	0.29564180	0.50000000
H	0.56547124	0.61625992	0.50000000

H 0.20743496 0.51942264 0.50000000
H 0.33764394 0.13697114 0.50000000
H 0.81656961 0.38802457 0.50000000
H 0.43332060 0.74536007 0.50000000
H 0.33757992 0.38812266 0.50000000
H 0.81647272 0.61617540 0.50000000
H 0.56556389 0.13680077 0.50000000
H 0.43339343 0.87548413 0.50000000
H 0.07742770 0.51956207 0.50000000
H 0.07756326 0.74574572 0.50000000
H 0.20765159 0.87580541 0.50000000

[PLY-CC-TOT]
a = 19.24, 19.24, c = 29.40, alpha = 90.0, beta = 90.0, gamma = 119.9
C 0.57191902 0.50818784 0.50000000
C 0.19677939 0.62815639 0.50000000
C 0.44482807 0.25071524 0.50000000
C 0.32449754 0.88324027 0.50000000
C 0.70239789 0.38093019 0.50000000
C 0.32499988 0.75618818 0.50000000
C 0.44472262 0.38101687 0.50000000
C 0.06986944 0.62900928 0.50000000
C 0.70225648 0.50813819 0.50000000
C 0.57214541 0.25067175 0.50000000
C 0.52973999 0.54931477 0.50000000
C 0.23997773 0.58670008 0.50000000
C 0.40379905 0.16750162 0.50000000
C 0.78564940 0.42305240 0.50000000
C 0.36630033 0.71284201 0.50000000
C 0.40360526 0.42320686 0.50000000
C 0.78548801 0.54920311 0.50000000
C 0.53004465 0.16745184 0.50000000
C 0.53024041 0.42268744 0.50000000
C 0.23953339 0.71360395 0.50000000
C 0.53034611 0.29445457 0.50000000
C 0.24015728 0.84101375 0.50000000
C 0.65852715 0.42264316 0.50000000
C 0.11224872 0.71332875 0.50000000
C 0.44533267 0.50759499 0.50000000
C 0.32431822 0.62851552 0.50000000
C 0.44563893 0.12487084 0.50000000
C 0.82818216 0.50743748 0.50000000
C 0.36650809 0.58625041 0.50000000
C 0.40313451 0.54976145 0.50000000

C 0.36678667 0.96758582 0.50000000
C 0.40348710 0.04061585 0.50000000
C 0.98550168 0.58674440 0.50000000
C 0.91245811 0.54974601 0.50000000
C 0.66054786 0.55511391 0.50000000
C 0.11221886 0.58695573 0.50000000
C 0.39781356 0.29241080 0.50000000
C 0.36640036 0.84074292 0.50000000
C 0.66078634 0.29232472 0.50000000
C 0.57303749 0.37992417 0.50000000
C 0.19683782 0.75645521 0.50000000
H 0.56362055 0.61472972 0.50000000
H 0.20766827 0.52125960 0.50000000
H 0.33836378 0.13597218 0.50000000
H 0.81722302 0.38913907 0.50000000
H 0.43175384 0.74500610 0.50000000
H 0.33817108 0.38935176 0.50000000
H 0.81697614 0.61461594 0.50000000
H 0.56397808 0.13589812 0.50000000
H 0.43185536 0.87383233 0.50000000
H 0.07896752 0.52151796 0.50000000
H 0.07906446 0.74565257 0.50000000
H 0.20795085 0.87431401 0.50000000
O 0.32329757 0.25518961 0.50000000
O 0.69809507 0.25506082 0.50000000
O 0.69776074 0.62961149 0.50000000

[PLY-CC-TRIH]
a = 19.30, 19.30, c = 29.36, alpha = 90.0, beta = 90.0, gamma = 120.0
C 0.57150938 0.50846425 0.49986803
C 0.19704847 0.62811370 0.50007840
C 0.44502414 0.25034218 0.49993591
C 0.32429085 0.88296518 0.50003244
C 0.70309301 0.38183955 0.49989576
C 0.32521977 0.75637929 0.50004316
C 0.44506123 0.38206455 0.49991719
C 0.07041197 0.62888703 0.50009076
C 0.70312780 0.50834126 0.49985142
C 0.57144473 0.25023576 0.49989805
C 0.52956873 0.54937759 0.49994321
C 0.24037402 0.58695585 0.50006699
C 0.40416224 0.16752264 0.49999736
C 0.78591011 0.42376756 0.49999293
C 0.36643048 0.71310647 0.50004524

C	0.40423251	0.42408327	0.49998348
C	0.78593958	0.54915844	0.49993785
C	0.52946387	0.16740909	0.49995238
C	0.53014561	0.42336393	0.49986426
C	0.23973540	0.71361384	0.50006921
C	0.53011051	0.29402883	0.49988810
C	0.24016642	0.84080550	0.50005647
C	0.65937022	0.42324366	0.49984959
C	0.11250239	0.71302515	0.50009338
C	0.44532209	0.50834699	0.49999602
C	0.32450057	0.62896111	0.50005419
C	0.44521786	0.12430014	0.49999775
C	0.82908952	0.50803001	0.50001336
C	0.36670588	0.58681985	0.50004408
C	0.40329623	0.55036971	0.50003168
C	0.36645726	0.96727597	0.50002126
C	0.40312761	0.04028758	0.50001655
C	0.98610435	0.58667169	0.50008076
C	0.91310084	0.55005472	0.50006919
C	0.66120789	0.55615809	0.49972869
C	0.11261051	0.58692801	0.50008821
C	0.39729521	0.29235418	0.49985016
C	0.36632668	0.84083492	0.50002611
C	0.66113988	0.29212806	0.49976839
C	0.57320688	0.38021036	0.49983206
C	0.19694185	0.75634208	0.50007765
H	0.56199919	0.61473943	0.49994304
H	0.20828184	0.52167720	0.50006094
H	0.33880965	0.13462823	0.50002692
H	0.81882117	0.39129509	0.50004129
H	0.43169813	0.74525216	0.50003764
H	0.33887807	0.39169737	0.50001287
H	0.81886518	0.61452044	0.49993826
H	0.56187459	0.13443595	0.49993565
H	0.43159219	0.87399194	0.50000541
H	0.35672010	0.27210413	0.52957980
H	0.68147897	0.27175311	0.52944378
H	0.07952440	0.52164955	0.50008289
H	0.68155625	0.59684073	0.52941284
H	0.07932170	0.74514276	0.50010730
H	0.20797442	0.87391268	0.50004865
H	0.68123490	0.59622925	0.46977105
H	0.35712631	0.27228405	0.46993777
H	0.68115856	0.27206777	0.46980661

[PLY-CC-TPM]

a = 19.33, 19.32, c = 29.36, alpha = 90.0, beta = 90.0, gamma = 120.1

H	0.54429226	0.59752169	0.53953970
H	0.20828601	0.52152187	0.49963839
H	0.35602121	0.13416557	0.53994201
H	0.81863205	0.40961272	0.54039623
H	0.43181849	0.74520221	0.50041046
H	0.35622706	0.40938980	0.45991727
H	0.81957609	0.59678061	0.45963315
H	0.54377605	0.13447666	0.46013899
H	0.43191735	0.87390975	0.49959261
H	0.07955703	0.52132959	0.50053698
H	0.07975372	0.74468646	0.49970585
H	0.20850346	0.87350781	0.50060353
H	0.61785978	0.52341295	0.53995489
H	0.43019979	0.28211253	0.54023067
H	0.67070314	0.33617573	0.54072663
H	0.43034307	0.33582679	0.45951854
H	0.67164412	0.52282550	0.45922454
H	0.61739231	0.28240413	0.45962747
C	0.56112719	0.49893623	0.52178770
C	0.19718466	0.62786462	0.50011148
C	0.45457064	0.24984042	0.52205524
C	0.32467745	0.88265444	0.50011129
C	0.70324916	0.39271670	0.52237368
C	0.32544233	0.75621670	0.50006333
C	0.45478852	0.39256116	0.47769389
C	0.07072015	0.62853046	0.50012797
C	0.70377660	0.49853078	0.47762171
C	0.56071423	0.25000834	0.47785549
C	0.52003345	0.54035314	0.52184045
C	0.24042618	0.58679709	0.49979351
C	0.41312093	0.16717356	0.52218061
C	0.78588722	0.43372474	0.52246666
C	0.36655710	0.71301721	0.50029739
C	0.41337372	0.43365461	0.47762597
C	0.78642714	0.53985921	0.47756915
C	0.51959741	0.16735340	0.47785940
C	0.52986272	0.42377724	0.49978188
C	0.23997645	0.71335092	0.50010764
C	0.52960774	0.29386874	0.49992695
C	0.24060710	0.84044800	0.50042798
C	0.65959602	0.42372888	0.49997347
C	0.11285270	0.71262154	0.49986296

C	0.44516637	0.50849559	0.49976156
C	0.32450029	0.62891511	0.50001291
C	0.44484046	0.12416212	0.50006110
C	0.82924291	0.50824606	0.50001803
C	0.36665256	0.58679447	0.49993380
C	0.40312772	0.55040150	0.49984062
C	0.36679005	0.96707793	0.50012160
C	0.40294791	0.04006640	0.50012573
C	0.98630438	0.58630432	0.50008563
C	0.91333058	0.55007956	0.50003501
C	0.11273560	0.58660671	0.50040576
C	0.36665656	0.84069156	0.49977729
C	0.57299231	0.38047926	0.49989017
C	0.19729248	0.75598893	0.50013705

[PLY-CCCC-TAM]

a = 23.67, 23.67, c = 29.40, alpha = 90.0, beta = 90.0, gamma = 121.1

C	0.72422342	0.46372883	0.50000000
C	0.35674347	0.61960649	0.50000000
C	0.61785755	0.25629494	0.50000000
C	0.46357175	0.82971151	0.50000000
C	0.82518390	0.36076791	0.50000000
C	0.46321081	0.72600709	0.50000000
C	0.61895543	0.35845240	0.50000000
C	0.25308858	0.61935244	0.50000000
C	0.82642283	0.46487039	0.50000000
C	0.72199182	0.25757628	0.50000000
C	0.69138965	0.49781915	0.50000000
C	0.39179881	0.58617019	0.50000000
C	0.58267585	0.18822805	0.50000000
C	0.89316866	0.39460596	0.50000000
C	0.49658842	0.69089649	0.50000000
C	0.58482755	0.39124985	0.50000000
C	0.89450180	0.50007090	0.50000000
C	0.68816810	0.18959941	0.50000000
C	0.68847049	0.39422327	0.50000000
C	0.39267925	0.69013117	0.50000000
C	0.68734919	0.29128368	0.50000000
C	0.39415052	0.79440835	0.50000000
C	0.79145280	0.39538692	0.50000000
C	0.28844505	0.68876291	0.50000000
C	0.62128933	0.46133567	0.50000000
C	0.46122516	0.62147625	0.50000000
C	0.61808788	0.15471694	0.50000000

C	0.92803334	0.46467672	0.50000000
C	0.49793743	0.89900190	0.50000000
C	0.58387367	0.08575287	0.50000000
C	0.18379268	0.58501453	0.50000000
C	0.99700704	0.49890556	0.50000000
C	0.49530848	0.58736455	0.50000000
C	0.58735223	0.49526458	0.50000000
C	0.28708384	0.58508714	0.50000000
C	0.49778683	0.79565858	0.50000000
C	0.05745971	0.52671956	0.50000000
C	0.12341432	0.55721446	0.50000000
C	0.55758133	0.52509003	0.50000000
C	0.52507991	0.55760886	0.50000000
C	0.55614018	0.02531545	0.50000000
C	0.52570865	0.95937193	0.50000000
C	0.72242558	0.36029796	0.50000000
C	0.35803161	0.72482989	0.50000000
O	0.79267031	0.49843476	0.50000000
O	0.58426401	0.29001607	0.50000000
O	0.79038091	0.29238655	0.50000000
H	0.71829318	0.55152055	0.50000000
H	0.36490941	0.53237053	0.50000000
H	0.52895797	0.16056811	0.50000000
H	0.91991325	0.36851139	0.50000000
H	0.55039393	0.71773991	0.50000000
H	0.53111490	0.36432297	0.50000000
H	0.92215502	0.55378268	0.50000000
H	0.71427572	0.16286903	0.50000000
H	0.55160453	0.82341625	0.50000000
H	0.25927422	0.53127941	0.50000000
H	0.36808123	0.82127386	0.50000000
H	0.26160652	0.71485996	0.50000000

[PLY-CCCC-TOT]

a = 23.81, 23.82, c = 29.40, alpha = 90.0, beta = 90.0, gamma = 121.0

C	0.72239116	0.46419471	0.50000000
C	0.35692311	0.62024226	0.50000000
C	0.61703002	0.25367309	0.50000000
C	0.46285746	0.82875112	0.50000000
C	0.82755806	0.36216538	0.50000000
C	0.46254615	0.72578333	0.50000000
C	0.61797878	0.35970787	0.50000000
C	0.25392681	0.61997254	0.50000000
C	0.82865221	0.46544574	0.50000000

C	0.72048311	0.25501128	0.50000000
C	0.68850807	0.49724059	0.50000000
C	0.39174799	0.58700983	0.50000000
C	0.58292734	0.18568914	0.50000000
C	0.89551668	0.39697113	0.50000000
C	0.49577081	0.69094719	0.50000000
C	0.58477828	0.39343982	0.50000000
C	0.89668616	0.49965283	0.50000000
C	0.68577667	0.18708796	0.50000000
C	0.68780952	0.39439969	0.50000000
C	0.39254571	0.69020412	0.50000000
C	0.68686562	0.29011205	0.50000000
C	0.39391300	0.79370828	0.50000000
C	0.79231530	0.39564017	0.50000000
C	0.28900869	0.68888010	0.50000000
C	0.61940263	0.46249629	0.50000000
C	0.46070103	0.62203264	0.50000000
C	0.61668428	0.15145680	0.50000000
C	0.93105627	0.46603054	0.50000000
C	0.49701778	0.89755762	0.50000000
C	0.58271686	0.08289282	0.50000000
C	0.18509271	0.58585375	0.50000000
C	0.99965466	0.50004402	0.50000000
C	0.49455276	0.58809202	0.50000000
C	0.58560631	0.49624920	0.50000000
C	0.79495673	0.50313488	0.50000000
C	0.28774351	0.58594953	0.50000000
C	0.57916167	0.28718093	0.50000000
C	0.49687568	0.79492669	0.50000000
C	0.79293349	0.28975376	0.50000000
C	0.05962895	0.52775742	0.50000000
C	0.12515901	0.55818713	0.50000000
C	0.55617905	0.52597623	0.50000000
C	0.52399427	0.55841913	0.50000000
C	0.55502538	0.02294913	0.50000000
C	0.52463833	0.95745541	0.50000000
C	0.72233138	0.36005778	0.50000000
C	0.35811335	0.72464107	0.50000000
H	0.71649366	0.55068582	0.50000000
H	0.36506588	0.53357598	0.50000000
H	0.52947134	0.15941067	0.50000000
H	0.92089582	0.36972603	0.50000000
H	0.54923424	0.71759496	0.50000000
H	0.53130691	0.36536841	0.50000000

H 0.92288898 0.55307784 0.50000000
H 0.71312857 0.16181672 0.50000000
H 0.55034874 0.82249700 0.50000000
H 0.26014231 0.53250670 0.50000000
H 0.36799954 0.82042620 0.50000000
H 0.26230505 0.71480807 0.50000000
O 0.51838673 0.25627099 0.50000000
O 0.82290659 0.25990371 0.50000000
O 0.82576286 0.56387599 0.50000000

[PLY-CCCC-TRIH]
a = 23.83, 23.85, c = 29.40, alpha = 90.0, beta = 90.0, gamma = 120.8
C 0.72199271 0.46406037 0.49992221
C 0.35689438 0.62111450 0.50003040
C 0.61748764 0.25334233 0.49979211
C 0.46200151 0.82906238 0.50003921
C 0.82831164 0.36225571 0.49973646
C 0.46204158 0.72630891 0.50004551
C 0.61826374 0.36033035 0.49991796
C 0.25391358 0.62086385 0.50001867
C 0.82918287 0.46505579 0.49987290
C 0.72047164 0.25440984 0.49969449
C 0.68813601 0.49696705 0.50015833
C 0.39186831 0.58798848 0.50009155
C 0.58375777 0.18576433 0.49994505
C 0.89589820 0.39684030 0.49992816
C 0.49534512 0.69150627 0.50009946
C 0.58524827 0.39408815 0.50017195
C 0.89681779 0.49886337 0.50008681
C 0.68596954 0.18688518 0.49988012
C 0.68778574 0.39459526 0.49981352
C 0.39220959 0.69088557 0.50001451
C 0.68701801 0.28959132 0.49968853
C 0.39325263 0.79415585 0.49999294
C 0.79299877 0.39557511 0.49973139
C 0.28871719 0.68956331 0.49997977
C 0.61924425 0.46291774 0.50029044
C 0.46062037 0.62281598 0.50014828
C 0.61708484 0.15107548 0.50000131
C 0.93164541 0.46567636 0.50011023
C 0.49612050 0.89770949 0.50008981
C 0.58320974 0.08278143 0.50011800
C 0.18521452 0.58666773 0.50008772
C 0.99999726 0.49960237 0.50023089

C	0.49456122	0.58889936	0.50026761
C	0.58550903	0.49670437	0.50043151
C	0.79528605	0.50351135	0.49967243
C	0.28790245	0.58697848	0.50002200
C	0.57887304	0.28708335	0.49966306
C	0.49605499	0.79523990	0.50004747
C	0.79368168	0.28911450	0.49942316
C	0.05992025	0.52771583	0.50023165
C	0.12535871	0.55854663	0.50018877
C	0.55612053	0.52656779	0.50044050
C	0.52396658	0.55912256	0.50036400
C	0.55509534	0.02290576	0.50014717
C	0.52425447	0.95751867	0.50013404
C	0.72260524	0.35992543	0.49965860
C	0.35765892	0.72526715	0.49998401
H	0.71487207	0.55027969	0.50020729
H	0.36539743	0.53474203	0.50011283
H	0.53039701	0.15850036	0.50001343
H	0.92245852	0.37070841	0.49991635
H	0.54863926	0.71806976	0.50011763
H	0.53189141	0.36730089	0.50025580
H	0.92402229	0.55218373	0.50019385
H	0.71219227	0.16041583	0.49991037
H	0.54935485	0.82267259	0.50007332
H	0.54554601	0.27016934	0.52923470
H	0.81023206	0.27258529	0.52889900
H	0.26057285	0.53372257	0.50004181
H	0.81218172	0.53689764	0.52919709
H	0.36727555	0.82080653	0.49997674
H	0.26194170	0.71541391	0.49996210
H	0.81170214	0.53601800	0.46966488
H	0.54626557	0.27063415	0.46969732
H	0.80973772	0.27314803	0.46936096

[PLY-CCCC-TPM]

a = 23.85, 23.86, c = 29.40, alpha = 90.0, beta = 90.0, gamma = 120.8

H	0.70006527	0.53574564	0.54083955
H	0.36512866	0.53484519	0.50000273
H	0.54615503	0.15775965	0.54009075
H	0.92153606	0.38415546	0.54085315
H	0.54827422	0.71813381	0.50069029
H	0.54654134	0.38293789	0.46001708
H	0.92484831	0.53663471	0.45954861
H	0.69851711	0.16124067	0.45845371

H	0.54903791	0.82267584	0.50023732
H	0.26034932	0.53381427	0.50014404
H	0.36714537	0.82085730	0.50019759
H	0.26178987	0.71543200	0.49963857
H	0.75952357	0.47604625	0.54057837
H	0.60592877	0.27840933	0.54011719
H	0.80083009	0.32434148	0.54096264
H	0.60613192	0.32336956	0.45949217
H	0.80410612	0.47673561	0.45950351
H	0.75820049	0.28187677	0.45830250
C	0.71329414	0.45604408	0.52238877
C	0.35664865	0.62118740	0.50014826
C	0.62581095	0.25276792	0.52168679
C	0.46177536	0.82906995	0.50022326
C	0.82796086	0.37043792	0.52261759
C	0.46174090	0.72635105	0.50032766
C	0.62630805	0.36945022	0.47786808
C	0.25376399	0.62092780	0.49987323
C	0.82981420	0.45684209	0.47784097
C	0.71213992	0.25472392	0.47671150
C	0.68010962	0.48940947	0.52282933
C	0.39159418	0.58808118	0.50011762
C	0.59239939	0.18537484	0.52195567
C	0.89535241	0.40378786	0.52283234
C	0.49500953	0.69157009	0.50053815
C	0.59299476	0.40269384	0.47787421
C	0.89724810	0.49032757	0.47758817
C	0.67886454	0.18736221	0.47652485
C	0.68751109	0.39503554	0.49999533
C	0.39195209	0.69094139	0.50018661
C	0.68681897	0.28956657	0.49930962
C	0.39308227	0.79420876	0.50021436
C	0.79305947	0.39577355	0.50011386
C	0.28853530	0.68959309	0.49979330
C	0.61918534	0.46348463	0.50044655
C	0.46028728	0.62291672	0.50036822
C	0.61829924	0.15117077	0.49938220
C	0.93150758	0.46442543	0.50008983
C	0.49613239	0.89776334	0.50020567
C	0.58497949	0.08288589	0.49972042
C	0.18503907	0.58652730	0.49975351
C	0.99983059	0.49782054	0.49985745
C	0.49426815	0.58904604	0.50042032
C	0.58556178	0.49734136	0.50054382

C 0.28769386 0.58705924 0.50010898
C 0.49576581 0.79526158 0.50021769
C 0.05973754 0.52638429 0.49978720
C 0.12519866 0.55779120 0.49973599
C 0.55608195 0.52706097 0.50053509
C 0.52381481 0.55947111 0.50046950
C 0.55636812 0.02300591 0.49992102
C 0.52492720 0.95757694 0.50009886
C 0.72237873 0.36020170 0.49976400
C 0.35745424 0.72531532 0.50006435

[PLY-Ph-TAM]
a = 22.09, 22.10, c = 29.40, alpha = 90.0, beta = 90.0, gamma = 120.0
C 0.74489347 0.48362987 0.49973447
C 0.37385663 0.63277596 0.50003363
C 0.63468279 0.26425916 0.49966913
C 0.48547101 0.85505401 0.50004856
C 0.85413289 0.37335644 0.49981865
C 0.63445298 0.37300779 0.50026956
C 0.48580127 0.74435882 0.50004592
C 0.85387741 0.48379799 0.50043840
C 0.26342673 0.63370363 0.50008562
C 0.74536027 0.26441523 0.50030746
C 0.70903140 0.51999795 0.49952939
C 0.41175059 0.59687601 0.50005730
C 0.59829957 0.19206494 0.49949692
C 0.92635487 0.40918350 0.49973861
C 0.59781990 0.40859921 0.50036737
C 0.52136454 0.70612677 0.49990295
C 0.92609434 0.52037588 0.50061330
C 0.70972912 0.19221479 0.50040056
C 0.58528944 0.61904466 0.47495352
C 0.53441024 0.49575964 0.52460852
C 0.49919113 0.96927714 0.47508421
C 0.62253195 0.04178377 0.52472438
C 0.14920035 0.53327612 0.47506327
C 0.07656656 0.58362761 0.52505922
C 0.49830276 0.53215396 0.52467223
C 0.62159131 0.58286110 0.47498120
C 0.14905935 0.61988088 0.52506406
C 0.07671506 0.49681694 0.47516486
C 0.58622522 0.96932369 0.52478713
C 0.53529853 0.04173306 0.47511412
C 0.70791713 0.41019405 0.50002356

C	0.41125830	0.70728623	0.50007416
C	0.70815082	0.30068276	0.50001053
C	0.41233477	0.81845659	0.50003532
C	0.81766101	0.41036018	0.50010805
C	0.30032132	0.70680830	0.50024442
C	0.63521757	0.48237531	0.49991093
C	0.48487753	0.63301177	0.49994741
C	0.63591365	0.15586942	0.49993296
C	0.96249535	0.48296390	0.50018936
C	0.52310654	0.59443469	0.49986378
C	0.59681701	0.52055510	0.49983099
C	0.52395212	0.93181121	0.49996550
C	0.59767245	0.07930553	0.49991936
C	0.18663303	0.59536167	0.50006370
C	0.03908407	0.52136636	0.50015709
C	0.74457086	0.37374837	0.50005177
C	0.37412485	0.74470248	0.50012981
C	0.30007891	0.59722112	0.49994940
C	0.52167888	0.81812613	0.50012629
O	0.81750561	0.52005089	0.50009918
O	0.59822590	0.30041987	0.49996556
O	0.81798648	0.30075574	0.50006043
H	0.60406662	0.66612816	0.45431268
H	0.51549030	0.44864773	0.54524485
H	0.45206838	0.94094521	0.45446691
H	0.66963896	0.07001601	0.54537262
H	0.17758924	0.51469819	0.45429996
H	0.04827821	0.60234625	0.54580002
H	0.45117263	0.51337389	0.54528782
H	0.66867282	0.60169469	0.45430566
H	0.17732827	0.66690910	0.54576334
H	0.04854252	0.44982921	0.45441749
H	0.60506172	0.94102369	0.54540531
H	0.51637413	0.06992854	0.45446806
H	0.73757712	0.57690740	0.50035408
H	0.38301573	0.53984713	0.49894179
H	0.54136695	0.16377830	0.50031294
H	0.95472780	0.38061949	0.50059946
H	0.54089003	0.37987430	0.49949480
H	0.57841392	0.73456932	0.50095875
H	0.95425019	0.57728333	0.49977498
H	0.73848749	0.16403437	0.49955150
H	0.57872800	0.84640004	0.49903186
H	0.27153705	0.54018882	0.50097627

H 0.38384167 0.84702003 0.50114433
H 0.27195468 0.73550260 0.49923016

[PLY-Ph-TOT]
a = 22.24, 22.24, c = 29.40, alpha = 90.0, beta = 90.0, gamma = 120.0
C 0.74337711 0.48440387 0.49960774
C 0.37421214 0.63312914 0.50006086
C 0.63361385 0.26165134 0.49973757
C 0.48491566 0.85398724 0.50024178
C 0.85642850 0.37471042 0.49973002
C 0.63349247 0.37441190 0.50012644
C 0.48518153 0.74392477 0.50022433
C 0.85629778 0.48453766 0.50024762
C 0.26429325 0.63372969 0.49996254
C 0.74362170 0.26182878 0.50030384
C 0.70646534 0.51966615 0.49933695
C 0.41188255 0.59752759 0.50006623
C 0.59833167 0.18947724 0.49964799
C 0.92862718 0.41161045 0.49957104
C 0.59807572 0.41117140 0.50019797
C 0.52062620 0.70610533 0.50017298
C 0.92849664 0.51995508 0.50042529
C 0.70686091 0.18965808 0.50054356
C 0.58444880 0.61998818 0.47505905
C 0.53360019 0.49707476 0.52451266
C 0.49823435 0.96725615 0.47536242
C 0.62106724 0.03936725 0.52493731
C 0.15094186 0.53382141 0.47506875
C 0.07891493 0.58461149 0.52461434
C 0.49779555 0.53327649 0.52472336
C 0.62049063 0.58402333 0.47502445
C 0.15094447 0.62053962 0.52468209
C 0.07891308 0.49766095 0.47517344
C 0.58496465 0.96735601 0.52504464
C 0.53409567 0.03925901 0.47543122
C 0.70738763 0.41053462 0.49990800
C 0.41125937 0.70703353 0.50014191
C 0.70749803 0.29956059 0.49999566
C 0.41222064 0.81732302 0.50013643
C 0.81852568 0.41067089 0.49997926
C 0.30110570 0.70639778 0.50004891
C 0.63366069 0.48395797 0.49971767
C 0.48457941 0.63342276 0.50008630
C 0.63406797 0.15244246 0.50014349

C	0.96570725	0.48439604	0.49998882
C	0.52264890	0.59521239	0.49997081
C	0.59557389	0.52198297	0.49971920
C	0.52308027	0.93025959	0.50022198
C	0.59608637	0.07637374	0.50018912
C	0.18799975	0.59568778	0.49989675
C	0.04182441	0.52255141	0.49993024
C	0.74446961	0.37358955	0.49996355
C	0.37438957	0.74405578	0.50011457
C	0.82008701	0.52501677	0.49995045
C	0.30091860	0.59768108	0.49995356
C	0.59298673	0.29770330	0.49992369
C	0.52080889	0.81720875	0.50031692
C	0.82032405	0.29803440	0.50001979
H	0.60323714	0.66692411	0.45454370
H	0.51460738	0.44991476	0.54480326
H	0.45133898	0.93910873	0.45479266
H	0.66822254	0.06756757	0.54524568
H	0.17907054	0.51505475	0.45451343
H	0.05074867	0.60358591	0.54492869
H	0.45094499	0.51451608	0.54533597
H	0.66758078	0.60300207	0.45464999
H	0.17905659	0.66748206	0.54520216
H	0.05075057	0.45049512	0.45487976
H	0.60377245	0.93927144	0.54560597
H	0.51511393	0.06738280	0.45508787
H	0.73630589	0.57632671	0.49985479
H	0.38336314	0.54084549	0.49898167
H	0.54165837	0.16263957	0.50018673
H	0.95544283	0.38177136	0.50013588
H	0.54140139	0.38121316	0.49961801
H	0.57732534	0.73450128	0.50124083
H	0.95522649	0.57661065	0.49988964
H	0.73682885	0.16297767	0.50002620
H	0.57751473	0.84539571	0.49936537
H	0.27258645	0.54099363	0.50091540
H	0.38381450	0.84560109	0.50111141
H	0.27294698	0.73492075	0.49901480
O	0.52840012	0.26533276	0.49991413
O	0.85264953	0.26579807	0.50003033
O	0.85235907	0.58958875	0.49997988

[PLY-Ph-TRIH]

a = 22.29, 22.31, c = 29.40, alpha = 90.0, beta = 90.0, gamma = 120.0

C	0.74279427	0.48424813	0.49955274
C	0.37448923	0.63344321	0.50002116
C	0.63373613	0.26119079	0.49953911
C	0.48458607	0.85378898	0.50023797
C	0.85699883	0.37524718	0.49939739
C	0.63358785	0.37504170	0.50029926
C	0.48522655	0.74416013	0.50019450
C	0.85683886	0.48437770	0.50018193
C	0.26469298	0.63388814	0.49994698
C	0.74309094	0.26128942	0.50016504
C	0.70607965	0.51935068	0.49938889
C	0.41227482	0.59808271	0.50007834
C	0.59864891	0.18941465	0.49934202
C	0.92883460	0.41195785	0.49918229
C	0.59835705	0.41165452	0.50059022
C	0.52068207	0.70645976	0.50011899
C	0.92867030	0.51958819	0.50046152
C	0.70649923	0.18951057	0.50045983
C	0.58448029	0.62015621	0.47524272
C	0.53389285	0.49798353	0.52490108
C	0.49823375	0.96707790	0.47524250
C	0.62041035	0.03897466	0.52489991
C	0.15136410	0.53428688	0.47487674
C	0.07933232	0.58414377	0.52483980
C	0.49821858	0.53418870	0.52497779
C	0.62027073	0.58406816	0.47523771
C	0.15121428	0.62003925	0.52489055
C	0.07948317	0.49826920	0.47490685
C	0.58435393	0.96714899	0.52506221
C	0.53416579	0.03890415	0.47515847
C	0.70713730	0.41076479	0.49989193
C	0.41136906	0.70723808	0.50011512
C	0.70728369	0.29902521	0.49982943
C	0.41207770	0.81720195	0.50011037
C	0.81909351	0.41088424	0.49979398
C	0.30122394	0.70634912	0.50009599
C	0.63347252	0.48421974	0.50000873
C	0.48478311	0.63400235	0.50010687
C	0.63388643	0.15204173	0.49991294
C	0.96621006	0.48452543	0.49984769
C	0.52282806	0.59586218	0.50012264
C	0.59563105	0.52226656	0.50003972
C	0.52266474	0.92992346	0.50020668
C	0.59596634	0.07622043	0.49997329

C 0.18850442   0.59578174   0.49989436
C 0.04209920   0.52252950   0.49987127
C 0.74450627   0.37355559   0.49981615
C 0.37437860   0.74409540   0.50011366
C 0.82045416   0.52565485   0.49977755
C 0.30132449   0.59801614   0.49987540
C 0.59228858   0.29742286   0.49988482
C 0.52057496   0.81726586   0.50032438
C 0.82076989   0.29762645   0.49969644
H 0.60325646   0.66686577   0.45462498
H 0.51505733   0.45124306   0.54548657
H 0.45150969   0.93900242   0.45463323
H 0.66715033   0.06700444   0.54550136
H 0.17945815   0.51577646   0.45415103
H 0.05127091   0.60269631   0.54556103
H 0.45152042   0.51567566   0.54560678
H 0.66697016   0.60262002   0.45460823
H 0.17922875   0.66659813   0.54562932
H 0.05149298   0.45166782   0.45420709
H 0.60296486   0.93914516   0.54577945
H 0.51545949   0.06685216   0.45446481
H 0.73468038   0.57593124   0.50002430
H 0.38393506   0.54158065   0.49894264
H 0.54202495   0.16141893   0.49999217
H 0.95689124   0.38339042   0.49975147
H 0.54172629   0.38301850   0.50000380
H 0.57722521   0.73479857   0.50122506
H 0.95657349   0.57617435   0.49990424
H 0.73519915   0.16158230   0.49986151
H 0.57712365   0.84545701   0.49929603
H 0.55718761   0.27950641   0.52960946
H 0.83879445   0.28047563   0.52938247
H 0.27319438   0.54150723   0.50086535
H 0.83769198   0.56084735   0.52944283
H 0.38364785   0.84532062   0.50113151
H 0.27301325   0.73468664   0.49905372
H 0.83812882   0.56044435   0.46990452
H 0.55734599   0.28033808   0.47007807
H 0.83787034   0.27985489   0.46985281

[PLY-Ph-TPM]
a = 22.34, 22.38, c = 29.40, alpha = 90.0, beta = 90.0, gamma = 120.1
H 0.61783247   0.67995954   0.47290356
H 0.50026527   0.43633579   0.52548038

H	0.43887977	0.93962663	0.47178904
H	0.68237176	0.06794370	0.52522559
H	0.17790132	0.50114516	0.47285410
H	0.05112683	0.61751821	0.52593249
H	0.43782239	0.50087616	0.52746220
H	0.68163405	0.61675317	0.47471672
H	0.17843915	0.68034059	0.52780637
H	0.05057077	0.43693330	0.47484132
H	0.61826977	0.94076486	0.52739331
H	0.50158991	0.06678334	0.47339340
H	0.72027070	0.56219975	0.53778914
H	0.38314754	0.54196859	0.49792491
H	0.55523243	0.16008542	0.53686580
H	0.95689888	0.39795609	0.53835485
H	0.55478901	0.39704819	0.46221836
H	0.57687153	0.73507442	0.50227892
H	0.95736417	0.56292987	0.46254678
H	0.72159092	0.16173326	0.46175245
H	0.57715368	0.84429021	0.49734400
H	0.27402756	0.54202570	0.50272544
H	0.38399067	0.84430835	0.50260851
H	0.27455702	0.73522083	0.49796931
H	0.78331109	0.49783080	0.53896698
H	0.61924945	0.28763102	0.53834491
H	0.82914266	0.33472013	0.53927934
H	0.61940816	0.33423869	0.46076518
H	0.78408969	0.28928995	0.46067237
H	0.82963259	0.49819087	0.46095151
C	0.73400182	0.47629954	0.52108669
C	0.37464732	0.63359837	0.50024870
C	0.64109880	0.26024445	0.52048125
C	0.48532278	0.85377906	0.49989312
C	0.85705438	0.38398033	0.52141920
C	0.64091497	0.38339008	0.47875095
C	0.48529073	0.74389831	0.50002817
C	0.85732158	0.47680306	0.47898437
C	0.26475715	0.63387352	0.50034852
C	0.73469219	0.26116528	0.47842793
C	0.69854215	0.51230516	0.52060998
C	0.41222032	0.59828062	0.49963548
C	0.60530069	0.18870297	0.51984121
C	0.92870829	0.41954123	0.52107537
C	0.60477286	0.41873365	0.47937150
C	0.52047183	0.70618750	0.50061571

C	0.92897322	0.51301506	0.47970264
C	0.69954567	0.18961986	0.47886126
C	0.59199790	0.62709872	0.48576423
C	0.52590831	0.48988938	0.51380878
C	0.49169868	0.96716903	0.48483953
C	0.62885706	0.03952439	0.51335139
C	0.15071395	0.52667581	0.48587944
C	0.07920164	0.59211738	0.51420533
C	0.49072009	0.52633867	0.51455233
C	0.62802277	0.59147224	0.48640351
C	0.15102198	0.62752643	0.51487138
C	0.07889186	0.49042831	0.48661483
C	0.59268022	0.96779797	0.51419489
C	0.52703208	0.03887362	0.48536029
C	0.70650126	0.41087539	0.49987053
C	0.41163415	0.70714276	0.50017495
C	0.70677577	0.29872889	0.49953237
C	0.41280484	0.81687122	0.50074399
C	0.81911011	0.41129941	0.50007253
C	0.30188197	0.70629220	0.49973449
C	0.63309572	0.48405110	0.50001855
C	0.48475253	0.63375152	0.50014010
C	0.63403136	0.15204717	0.49931517
C	0.96601344	0.48486040	0.50043908
C	0.52271160	0.59555396	0.50016109
C	0.59537450	0.52203438	0.50005847
C	0.52347477	0.92999031	0.49963688
C	0.59641447	0.07627151	0.49930378
C	0.18842984	0.59582815	0.50037776
C	0.04191250	0.52273919	0.50044964
C	0.74407385	0.37362719	0.49979143
C	0.37494437	0.74392124	0.50023467
C	0.30157723	0.59833428	0.50091708
C	0.52077122	0.81687377	0.49927221

[TRI-CC-TAM]

a = 21.56, 21.56, c = 29.40, alpha = 90.0, beta = 90.0, gamma = 120.0

C	0.66735474	0.44625827	0.50000000
C	0.33295279	0.55183191	0.50000000
C	0.55371093	0.22109312	0.50000000
C	0.44819136	0.78111872	0.50000000
C	0.77886423	0.33270847	0.50000000
C	0.21891906	0.66699470	0.50000000
C	0.44816816	0.66704767	0.50000000

C	0.55374066	0.33264303	0.50000000
C	0.21888105	0.55180742	0.50000000
C	0.77890548	0.44629061	0.50000000
C	0.33300386	0.78108028	0.50000000
C	0.66729538	0.22113777	0.50000000
C	0.63108842	0.48386880	0.50000000
C	0.37180840	0.51509457	0.50000000
C	0.51607730	0.14719038	0.50000000
C	0.48495251	0.85673390	0.50000000
C	0.85276275	0.36898781	0.50000000
C	0.14331045	0.62812734	0.50000000
C	0.48490584	0.62819237	0.50000000
C	0.51612879	0.36890813	0.50000000
C	0.14326590	0.51504575	0.50000000
C	0.85280806	0.48392644	0.50000000
C	0.37187092	0.85668903	0.50000000
C	0.63101877	0.14723886	0.50000000
C	0.62918387	0.37081521	0.50000000
C	0.37140283	0.62859720	0.50000000
C	0.62915128	0.25840087	0.50000000
C	0.37142829	0.74277156	0.50000000
C	0.74159905	0.37085012	0.50000000
C	0.25722786	0.62857066	0.50000000
C	0.55507257	0.44492407	0.50000000
C	0.44716505	0.55283600	0.50000000
C	0.55501163	0.11015066	0.50000000
C	0.44721604	0.89431508	0.50000000
C	0.88984976	0.44499392	0.50000000
C	0.10568469	0.55278235	0.50000000
C	0.48484272	0.51515742	0.50000000
C	0.51751773	0.48247907	0.50000000
C	0.48487601	0.96967512	0.50000000
C	0.51746535	0.03503604	0.50000000
C	0.03032521	0.51512378	0.50000000
C	0.96496372	0.48253825	0.50000000
C	0.25749752	0.51499918	0.50000000
C	0.48500004	0.74250294	0.50000000
C	0.25755628	0.74244246	0.50000000
C	0.66664548	0.33335445	0.50000000
C	0.33335300	0.66664658	0.50000000
O	0.74170982	0.48341223	0.50000000
O	0.51658812	0.25828770	0.50000000
O	0.74163638	0.25836765	0.50000000
H	0.65989418	0.54220527	0.50000000

H 0.34310343 0.45664213 0.50000000
H 0.45774149 0.11762745 0.50000000
H 0.54340469 0.88651651 0.50000000
H 0.88228963 0.34019757 0.50000000
H 0.11356724 0.65681755 0.50000000
H 0.54335851 0.65689821 0.50000000
H 0.45779215 0.34010121 0.50000000
H 0.11348374 0.45659366 0.50000000
H 0.88236979 0.54226214 0.50000000
H 0.34318025 0.88643160 0.50000000
H 0.65980913 0.11771196 0.50000000
H 0.54355468 0.77179406 0.50000000
H 0.22828787 0.77171001 0.50000000
H 0.22820722 0.45644484 0.50000000

[TRI-CC-TOT]
a = 21.70, 21.71, c = 29.40, alpha = 90.0, beta = 90.0, gamma = 120.0
C 0.66552118 0.44677693 0.50000000
C 0.33285191 0.55244700 0.50000000
C 0.55262812 0.21828729 0.50000000
C 0.44729570 0.78000256 0.50000000
C 0.78115764 0.33404041 0.50000000
C 0.21968304 0.66673035 0.50000000
C 0.44726758 0.66683696 0.50000000
C 0.55266441 0.33394605 0.50000000
C 0.21965415 0.55238928 0.50000000
C 0.78120110 0.44682860 0.50000000
C 0.33290690 0.77993965 0.50000000
C 0.66545992 0.21835361 0.50000000
C 0.62816489 0.48322480 0.50000000
C 0.37141822 0.51597535 0.50000000
C 0.51614067 0.14445432 0.50000000
C 0.48380634 0.85506807 0.50000000
C 0.85500243 0.37141754 0.50000000
C 0.14460574 0.62812960 0.50000000
C 0.48374298 0.62827281 0.50000000
C 0.51620880 0.37129408 0.50000000
C 0.14457079 0.51586152 0.50000000
C 0.85505073 0.48331888 0.50000000
C 0.37149748 0.85499879 0.50000000
C 0.62808263 0.14452895 0.50000000
C 0.62849723 0.37096915 0.50000000
C 0.37104494 0.62865398 0.50000000
C 0.62845915 0.25712837 0.50000000

C	0.37107306	0.74193763	0.50000000
C	0.74235835	0.37101878	0.50000000
C	0.25771719	0.62859663	0.50000000
C	0.55325141	0.44618770	0.50000000
C	0.44621673	0.55348968	0.50000000
C	0.55317695	0.10663844	0.50000000
C	0.44628926	0.89229225	0.50000000
C	0.89289813	0.44630731	0.50000000
C	0.10731388	0.55335029	0.50000000
C	0.48360717	0.51604070	0.50000000
C	0.51592632	0.48355637	0.50000000
C	0.48366538	0.96713069	0.50000000
C	0.51587800	0.03194130	0.50000000
C	0.03244992	0.51593729	0.50000000
C	0.96761877	0.48364421	0.50000000
C	0.74419879	0.48844778	0.50000000
C	0.25792402	0.51578420	0.50000000
C	0.51100051	0.25528376	0.50000000
C	0.48392058	0.74175063	0.50000000
C	0.74412861	0.25538871	0.50000000
C	0.25798513	0.74164204	0.50000000
C	0.66643668	0.33303636	0.50000000
C	0.33327568	0.66639286	0.50000000
H	0.65817924	0.54126611	0.50000000
H	0.34297979	0.45792593	0.50000000
H	0.45808877	0.11639302	0.50000000
H	0.54187061	0.88474652	0.50000000
H	0.88301407	0.34141166	0.50000000
H	0.11496967	0.65652257	0.50000000
H	0.54180658	0.65670669	0.50000000
H	0.45815157	0.34125977	0.50000000
H	0.11491664	0.45781110	0.50000000
H	0.88311209	0.54135997	0.50000000
H	0.34308840	0.88462070	0.50000000
H	0.65808769	0.11651678	0.50000000
H	0.54207594	0.77085118	0.50000000
H	0.22892687	0.77070605	0.50000000
H	0.22883838	0.45764079	0.50000000
O	0.44492576	0.22223455	0.50000000
O	0.77715241	0.22237452	0.50000000
O	0.77725036	0.55451168	0.50000000

[TRI-CC-TRIH]
a = 21.76, 21.76, c = 29.40, alpha = 90.0, beta = 90.0, gamma = 120.0

C	0.66532106	0.44723660	0.49994659
C	0.33303363	0.55291702	0.49998284
C	0.55297639	0.21820542	0.49992118
C	0.44721442	0.78016125	0.50000461
C	0.78194996	0.33496435	0.50007168
C	0.22001861	0.66703186	0.50000837
C	0.44718189	0.66710186	0.49998966
C	0.55307572	0.33503082	0.49997360
C	0.21998491	0.55288558	0.49999963
C	0.78205934	0.44721067	0.49993332
C	0.33308618	0.78011992	0.50000465
C	0.66514511	0.21819106	0.50008599
C	0.62813145	0.48349649	0.50003785
C	0.37158063	0.51653806	0.49999132
C	0.51669328	0.14472369	0.49996302
C	0.48363234	0.85511551	0.50000531
C	0.85541361	0.37216596	0.50000235
C	0.14507805	0.62846748	0.50000810
C	0.48357329	0.62857342	0.49999698
C	0.51687611	0.37227860	0.50009084
C	0.14504159	0.51646378	0.50000197
C	0.85552870	0.48343344	0.50000827
C	0.37164503	0.85507399	0.50000105
C	0.62788350	0.14471426	0.50004481
C	0.62857908	0.37171649	0.49995767
C	0.37111996	0.62899271	0.49998871
C	0.62848484	0.25701110	0.49999372
C	0.37115213	0.74215126	0.50000406
C	0.74319623	0.37168907	0.49999228
C	0.25798036	0.62895902	0.50000352
C	0.55337954	0.44704272	0.50012477
C	0.44624341	0.55390452	0.50001542
C	0.55313948	0.10644845	0.50000019
C	0.44630001	0.89240796	0.50000139
C	0.89373966	0.44692054	0.50000319
C	0.10775012	0.55379989	0.50000344
C	0.48365855	0.51657551	0.50007787
C	0.51613174	0.48428646	0.50013423
C	0.48365354	0.96716417	0.49999773
C	0.51590320	0.03194204	0.49999694
C	0.03300444	0.51643199	0.49999879
C	0.96823413	0.48412823	0.49999905
C	0.74490949	0.48961980	0.49969403
C	0.25826041	0.51641500	0.49999082

C 0.51063911   0.25543145   0.49970412
C 0.48367804   0.74188287   0.49999821
C 0.74472183   0.25537260   0.50031850
C 0.25831878   0.74181302   0.50000548
C 0.66675119   0.33346960   0.49996370
C 0.33341691   0.66670139   0.49999918
H 0.65691683   0.54149260   0.50000422
H 0.34317134   0.45860982   0.49999210
H 0.45870700   0.11548369   0.49993196
H 0.54155224   0.88464525   0.50000375
H 0.88460016   0.34338184   0.49997081
H 0.11558777   0.65688077   0.50000584
H 0.54149243   0.65700985   0.49999844
H 0.45889035   0.34353807   0.50011856
H 0.11552103   0.45853497   0.49999896
H 0.88481511   0.54142878   0.50004180
H 0.34325620   0.88458723   0.49999971
H 0.65659521   0.11545622   0.50007871
H 0.54169572   0.77090281   0.50000057
H 0.47430169   0.23723629   0.52921810
H 0.22932510   0.77082158   0.50000135
H 0.76285574   0.23719391   0.47079517
H 0.22924320   0.45838856   0.49999785
H 0.76310573   0.52596700   0.52920771
H 0.76258023   0.52493339   0.46966184
H 0.47533291   0.23781422   0.46967199
H 0.76238316   0.23771424   0.53033968

[TRI-CC-Ph3]
a = 21.79, 21.78, c = 29.40, alpha = 90.0, beta = 90.0, gamma = 120.1
H 0.64061929   0.52564224   0.54048615
H 0.34310888   0.45866031   0.49914121
H 0.47472719   0.11511399   0.54044886
H 0.54159869   0.88460367   0.49912326
H 0.88458427   0.35964167   0.54019912
H 0.11582584   0.65649288   0.49887838
H 0.54138339   0.65706530   0.50101675
H 0.47474088   0.35978723   0.45982020
H 0.11558392   0.45833740   0.50075211
H 0.88515677   0.52540998   0.45939179
H 0.34347521   0.88427612   0.50097754
H 0.64049625   0.11563841   0.45974147
H 0.54168554   0.77091225   0.50000866
H 0.22955634   0.77047659   0.49995617

H	0.22925583	0.45830727	0.49996077
H	0.70597680	0.45995948	0.54070556
H	0.54036513	0.24642274	0.54067509
H	0.75388915	0.45991795	0.45934195
H	0.70577913	0.24692962	0.45934125
H	0.75332809	0.29449284	0.54074269
H	0.54042185	0.29442735	0.45946006
C	0.65581052	0.43846492	0.52239701
C	0.33302781	0.55288222	0.49978173
C	0.56184316	0.21780670	0.52235569
C	0.44727988	0.78006701	0.49977742
C	0.78217066	0.34458253	0.52233434
C	0.22019927	0.66676605	0.49969408
C	0.44716552	0.66709252	0.50024652
C	0.56192209	0.34458746	0.47780088
C	0.22006459	0.55271949	0.50014855
C	0.78248910	0.43841412	0.47764394
C	0.33325034	0.77988169	0.50021380
C	0.65566312	0.21809757	0.47770722
C	0.61931849	0.47516572	0.52253923
C	0.37153864	0.51657063	0.49949680
C	0.52517524	0.14447942	0.52249480
C	0.48371094	0.85503388	0.49947554
C	0.85546289	0.38093550	0.52228825
C	0.14526979	0.62813768	0.49927825
C	0.48349544	0.62860151	0.50063599
C	0.52522287	0.38107691	0.47773706
C	0.14512711	0.51624673	0.50041435
C	0.85579669	0.47499692	0.47740216
C	0.37184031	0.85484238	0.50059555
C	0.61922913	0.14477929	0.47766533
C	0.62831370	0.37207326	0.50008115
C	0.37114240	0.62893393	0.49999772
C	0.62819331	0.25691203	0.50002511
C	0.37125920	0.74200282	0.49998340
C	0.74339597	0.37209940	0.50003825
C	0.25808959	0.62876772	0.49994008
C	0.55318775	0.44720736	0.50015785
C	0.44612782	0.55398619	0.50010009
C	0.55316197	0.10641634	0.50011819
C	0.44642183	0.89220654	0.50006844
C	0.89382178	0.44695585	0.49975552
C	0.10794468	0.55352300	0.49979176
C	0.48352734	0.51664449	0.50016021

C 0.51593915 0.48441205 0.50017826
C 0.48382303 0.96707804 0.50012722
C 0.51601678 0.03183491 0.50015211
C 0.03310606 0.51611224 0.49969934
C 0.96837365 0.48400813 0.49965845
C 0.25828527 0.51631881 0.49996363
C 0.48369462 0.74185982 0.50000628
C 0.25851251 0.74153200 0.49995712
C 0.66661387 0.33371896 0.50005665
C 0.33349566 0.66656865 0.49996963

[TRI-CCCC-TAM]
a = 26.01, 26.01, c = 29.40, alpha = 90.0, beta = 90.0, gamma = 120.0
C 0.66724408 0.42697160 0.50000000
C 0.33300703 0.57150593 0.50000000
C 0.57302019 0.24028793 0.50000000
C 0.42852181 0.76152504 0.50000000
C 0.75968839 0.33277597 0.50000000
C 0.23850307 0.66694258 0.50000000
C 0.42849410 0.66699297 0.50000000
C 0.57302843 0.33275592 0.50000000
C 0.23847494 0.57147822 0.50000000
C 0.75971205 0.42697984 0.50000000
C 0.33305742 0.76149690 0.50000000
C 0.66722403 0.24031158 0.50000000
C 0.63722005 0.45817009 0.50000000
C 0.36516742 0.54101588 0.50000000
C 0.54181821 0.17904933 0.50000000
C 0.45904250 0.82419544 0.50000000
C 0.82092063 0.36280416 0.50000000
C 0.17583363 0.63475959 0.50000000
C 0.45898412 0.63483258 0.50000000
C 0.54182991 0.36277995 0.50000000
C 0.17580456 0.54095750 0.50000000
C 0.82095067 0.45818179 0.50000000
C 0.36524041 0.82416637 0.50000000
C 0.63719584 0.17907937 0.50000000
C 0.63559226 0.36440774 0.50000000
C 0.36487557 0.63512443 0.50000000
C 0.63558238 0.27121281 0.50000000
C 0.36490298 0.72975165 0.50000000
C 0.72878716 0.36441762 0.50000000
C 0.27024832 0.63509702 0.50000000
C 0.57412454 0.42587546 0.50000000

C 0.42766608 0.57233392 0.50000000
C 0.57411418 0.14828340 0.50000000
C 0.42773381 0.85535442 0.50000000
C 0.85171658 0.42588582 0.50000000
C 0.14464556 0.57226619 0.50000000
C 0.45892091 0.91772659 0.50000000
C 0.54307855 0.08620604 0.50000000
C 0.08227342 0.54107906 0.50000000
C 0.91379398 0.45692142 0.50000000
C 0.45885161 0.54114836 0.50000000
C 0.54308450 0.45691547 0.50000000
C 0.27046853 0.54095961 0.50000000
C 0.45904042 0.72953147 0.50000000
C 0.27051874 0.72948126 0.50000000
C 0.96832202 0.48417297 0.50000000
C 0.02779919 0.51387353 0.50000000
C 0.51582388 0.48417609 0.50000000
C 0.48608413 0.51391585 0.50000000
C 0.51582700 0.03167796 0.50000000
C 0.48612644 0.97220079 0.50000000
C 0.66665121 0.33334882 0.50000000
C 0.33334184 0.66665819 0.50000000
O 0.72886135 0.45772441 0.50000000
O 0.54227562 0.27113865 0.50000000
O 0.72883107 0.27116893 0.50000000
H 0.66110317 0.50652251 0.50000000
H 0.34139159 0.49257483 0.50000000
H 0.49346736 0.15454811 0.50000000
H 0.50748331 0.84887903 0.50000000
H 0.84538863 0.33893608 0.50000000
H 0.15117086 0.65852253 0.50000000
H 0.50742517 0.65860841 0.50000000
H 0.49347749 0.33889683 0.50000000
H 0.15112099 0.49251669 0.50000000
H 0.84545191 0.50653264 0.50000000
H 0.34147747 0.84882915 0.50000000
H 0.66106392 0.15461139 0.50000000
H 0.50755959 0.75379510 0.50000000
H 0.24626627 0.75373373 0.50000000
H 0.24620490 0.49244041 0.50000000

[TRI-CCCC-TOT]
a = 26.15, 26.16, c = 29.40, alpha = 90.0, beta = 90.0, gamma = 120.0
C 0.66561863 0.42737464 0.50000000

C	0.33297520	0.57192306	0.50000000
C	0.57196810	0.23774553	0.50000000
C	0.42792004	0.76075842	0.50000000
C	0.76168631	0.33383434	0.50000000
C	0.23898908	0.66676143	0.50000000
C	0.42791642	0.66683112	0.50000000
C	0.57199239	0.33376869	0.50000000
C	0.23899704	0.57188834	0.50000000
C	0.76170470	0.42742100	0.50000000
C	0.33297532	0.76072056	0.50000000
C	0.66561520	0.23778951	0.50000000
C	0.63467809	0.45767175	0.50000000
C	0.36495409	0.54161223	0.50000000
C	0.54164522	0.17650618	0.50000000
C	0.45825613	0.82305110	0.50000000
C	0.82295231	0.36477130	0.50000000
C	0.17666428	0.63476211	0.50000000
C	0.45824423	0.63486466	0.50000000
C	0.54166980	0.36468293	0.50000000
C	0.17667850	0.54154023	0.50000000
C	0.82297511	0.45774282	0.50000000
C	0.36496100	0.82301352	0.50000000
C	0.63467528	0.17655171	0.50000000
C	0.63492544	0.36446988	0.50000000
C	0.36465335	0.63515752	0.50000000
C	0.63491495	0.26999383	0.50000000
C	0.36465645	0.72917654	0.50000000
C	0.72945730	0.36450937	0.50000000
C	0.27057723	0.63512376	0.50000000
C	0.57241830	0.42691318	0.50000000
C	0.42709091	0.57275567	0.50000000
C	0.57242475	0.14507066	0.50000000
C	0.42709466	0.85398486	0.50000000
C	0.85445014	0.42699961	0.50000000
C	0.14570470	0.57265950	0.50000000
C	0.45809508	0.91597172	0.50000000
C	0.54154751	0.08329042	0.50000000
C	0.08368334	0.54160161	0.50000000
C	0.91627403	0.45788581	0.50000000
C	0.45806373	0.54171863	0.50000000
C	0.54149584	0.45779758	0.50000000
C	0.73094938	0.46196260	0.50000000
C	0.27077773	0.54150865	0.50000000
C	0.53739086	0.26846537	0.50000000

C 0.45832779 0.72900424 0.50000000
C 0.73094811 0.26852977 0.50000000
C 0.27077678 0.72893336 0.50000000
C 0.97042447 0.48489481 0.50000000
C 0.02956962 0.51446582 0.50000000
C 0.51445558 0.48488741 0.50000000
C 0.48497044 0.51453246 0.50000000
C 0.51456481 0.02916994 0.50000000
C 0.48508602 0.97006040 0.50000000
C 0.66643525 0.33299385 0.50000000
C 0.33329700 0.66648712 0.50000000
H 0.65957618 0.50582850 0.50000000
H 0.34135497 0.49345130 0.50000000
H 0.49346683 0.15320287 0.50000000
H 0.50643742 0.84764844 0.50000000
H 0.84621338 0.33987964 0.50000000
H 0.15206600 0.65831939 0.50000000
H 0.50642526 0.65845561 0.50000000
H 0.49349158 0.33977021 0.50000000
H 0.15208699 0.49337950 0.50000000
H 0.84627006 0.50589823 0.50000000
H 0.34137855 0.84758630 0.50000000
H 0.65955881 0.15328177 0.50000000
H 0.50658974 0.75313518 0.50000000
H 0.24664925 0.75304151 0.50000000
H 0.24664589 0.49326777 0.50000000
O 0.48259775 0.24109275 0.50000000
O 0.75834321 0.24115591 0.50000000
O 0.75832399 0.51673190 0.50000000

[TRI-CCCC-TRIH]
a = 26.21, 26.21, c = 29.40, alpha = 90.0, beta = 90.0, gamma = 120.0
C 0.66557456 0.42789653 0.49998937
C 0.33306806 0.57224129 0.50000124
C 0.57229279 0.23777018 0.49993597
C 0.42785109 0.76087279 0.50000029
C 0.76239169 0.33466759 0.50002396
C 0.23923877 0.66697041 0.50000091
C 0.42782613 0.66700079 0.50000083
C 0.57238445 0.33473620 0.50003413
C 0.23920939 0.57222435 0.49999988
C 0.76246646 0.42786251 0.49998311
C 0.33311909 0.76085177 0.50000031
C 0.66542092 0.23774816 0.50006726

C 0.63475192	0.45802839	0.50001969
C 0.36501493	0.54198154	0.50000097
C 0.54213959	0.17679620	0.49996794
C 0.45812646	0.82310133	0.50000050
C 0.82335571	0.36549306	0.49992301
C 0.17701902	0.63500996	0.50000096
C 0.45807615	0.63504204	0.50000009
C 0.54229216	0.36559425	0.50012947
C 0.17698989	0.54195156	0.49999955
C 0.82342944	0.45796817	0.50007388
C 0.36508132	0.82307834	0.50000005
C 0.63454925	0.17677614	0.50003366
C 0.63507340	0.36519381	0.50001735
C 0.36468569	0.63538648	0.50000063
C 0.63498610	0.26998264	0.49999957
C 0.36471128	0.72932776	0.50000025
C 0.73021064	0.36516071	0.50000164
C 0.27075406	0.63536894	0.50000027
C 0.57261722	0.42773165	0.50009620
C 0.42704299	0.57300563	0.49999804
C 0.57241933	0.14496537	0.49999783
C 0.42710346	0.85408114	0.50000029
C 0.85519265	0.42762594	0.49999660
C 0.14601957	0.57298230	0.50000020
C 0.45808230	0.91604115	0.50000062
C 0.54158271	0.08330985	0.49999721
C 0.08407068	0.54200396	0.50000064
C 0.91683293	0.45843584	0.49999798
C 0.45801586	0.54201978	0.49999181
C 0.54177463	0.45853692	0.50006080
C 0.73163498	0.46306222	0.49972847
C 0.27098460	0.54193728	0.50000048
C 0.53716229	0.26867281	0.49972806
C 0.45813528	0.72909236	0.50000050
C 0.73147060	0.26860725	0.50029674
C 0.27103750	0.72905781	0.50000063
C 0.97094451	0.48548236	0.50000204
C 0.03001664	0.51499734	0.50000193
C 0.51464772	0.48551956	0.50001346
C 0.48505565	0.51500886	0.49999070
C 0.51457218	0.02918922	0.50000062
C 0.48507539	0.97010398	0.50000117
C 0.66675700	0.33344500	0.49999500
C 0.33338259	0.66669258	0.50000024

H	0.65863556	0.50616106	0.49993456
H	0.34140510	0.49390563	0.50000126
H	0.49401284	0.15253976	0.49994152
H	0.50619578	0.84758758	0.50000090
H	0.84761101	0.34163393	0.49985092
H	0.15255092	0.65860050	0.50000140
H	0.50614698	0.65864799	0.49999997
H	0.49416593	0.34174539	0.50013991
H	0.15250121	0.49387606	0.49999948
H	0.84774583	0.50609903	0.50014731
H	0.34149153	0.84754691	0.50000001
H	0.65838791	0.15250035	0.50005722
H	0.50629119	0.75317895	0.50000043
H	0.50694920	0.25353863	0.52920390
H	0.24696745	0.75313210	0.50000064
H	0.74653736	0.25348228	0.47081096
H	0.24690001	0.49377639	0.50000037
H	0.74678413	0.49328097	0.52920332
H	0.74627700	0.49232024	0.46967130
H	0.50790607	0.25409841	0.46967229
H	0.74610522	0.25396570	0.53033742

[TRI-CCCC-TPM]

a = 26.23, 26.23, c = 29.40, alpha = 90.0, beta = 90.0, gamma = 120.1

H	0.64473078	0.49270035	0.54076730
H	0.34139641	0.49385708	0.49956209
H	0.50757878	0.15217028	0.54089282
H	0.50642353	0.84760395	0.49963480
H	0.84745641	0.35545550	0.54091433
H	0.15270686	0.65833615	0.49964356
H	0.50621811	0.65869778	0.50045865
H	0.50750948	0.35545406	0.45918342
H	0.15251621	0.49366087	0.50038191
H	0.84802535	0.49259944	0.45913181
H	0.34177134	0.84741976	0.50041198
H	0.64465580	0.15267498	0.45907888
H	0.50643438	0.75320768	0.50004368
H	0.24718050	0.75293057	0.50001750
H	0.24689812	0.49364199	0.49997764
H	0.69914379	0.43829174	0.54079400
H	0.56192315	0.26120384	0.54087229
H	0.73844968	0.30114069	0.54098060
H	0.56193943	0.30105680	0.45913695
H	0.73900342	0.43828027	0.45913760

H	0.69900059	0.26169713	0.45901412
C	0.65753250	0.42050729	0.52241315
C	0.33310125	0.57216574	0.49991266
C	0.57968200	0.23742903	0.52246539
C	0.42802024	0.76082677	0.49993525
C	0.76244670	0.34271793	0.52255870
C	0.23939246	0.66679119	0.49993334
C	0.42791838	0.66699347	0.50014590
C	0.57970432	0.34266168	0.47752923
C	0.23928054	0.57206773	0.50010788
C	0.76275868	0.42050112	0.47754419
C	0.33330890	0.76071756	0.50011862
C	0.65742794	0.23770669	0.47743713
C	0.62719517	0.45091567	0.52266739
C	0.36502529	0.54194304	0.49975071
C	0.54931345	0.17657813	0.52273987
C	0.45834221	0.82307606	0.49979113
C	0.82328219	0.37298520	0.52278720
C	0.17715182	0.63477552	0.49979502
C	0.45813709	0.63506609	0.50029654
C	0.54929358	0.37299585	0.47727548
C	0.17703965	0.54174714	0.50024524
C	0.82360230	0.45084799	0.47726685
C	0.36532415	0.82296687	0.50026281
C	0.62713766	0.17686196	0.47720356
C	0.63475440	0.36544159	0.49997773
C	0.36477050	0.63532042	0.50003331
C	0.63467759	0.26988053	0.49995413
C	0.36487227	0.72923615	0.50002942
C	0.73029125	0.36548972	0.50004137
C	0.27086807	0.63522113	0.50002471
C	0.57237733	0.42782113	0.49996784
C	0.42704480	0.57303939	0.50002282
C	0.57239528	0.14495632	0.49999125
C	0.42733648	0.85398133	0.50003013
C	0.85519753	0.42772564	0.49999540
C	0.14613653	0.57275502	0.50002388
C	0.45836544	0.91599398	0.50003178
C	0.54167166	0.08328204	0.50002034
C	0.08413013	0.54172498	0.50001840
C	0.91686207	0.45841823	0.49996214
C	0.45801505	0.54208366	0.50000830
C	0.54158028	0.45861084	0.49996097
C	0.27100980	0.54180769	0.49999635

C	0.45827356	0.72909100	0.50004045
C	0.27122121	0.72888588	0.50002375
C	0.97097750	0.48531580	0.49997530
C	0.03008301	0.51477060	0.49999946
C	0.51455379	0.48562315	0.49996220
C	0.48502081	0.51511660	0.49998082
C	0.51476838	0.02916244	0.50002237
C	0.48531322	0.97005061	0.50002538
C	0.66655662	0.33362214	0.49999098
C	0.33350485	0.66659204	0.50002988

[TRI-Ph-TAM]

a = 24.57, 24.57, c = 29.40, alpha = 90.0, beta = 90.0, gamma = 120.1

C	0.66711130	0.43241325	0.49965737
C	0.33306715	0.56588309	0.50016634
C	0.56750879	0.23491838	0.49968495
C	0.43421072	0.76702381	0.50014493
C	0.76497911	0.33301989	0.49966114
C	0.23309581	0.66675720	0.50019752
C	0.56757240	0.33286784	0.50034031
C	0.43412344	0.66694166	0.49983094
C	0.76507195	0.43249601	0.50031738
C	0.23298259	0.56579154	0.49985005
C	0.66699328	0.23502591	0.50034165
C	0.33323793	0.76690194	0.49979039
C	0.63499187	0.46521620	0.49945165
C	0.36749112	0.53390886	0.50018974
C	0.53464891	0.16991953	0.49949116
C	0.46625615	0.83350767	0.50015793
C	0.82996139	0.36518754	0.49945801
C	0.16662409	0.63229834	0.50024641
C	0.53475936	0.36497585	0.50054425
C	0.46610408	0.63252195	0.49981332
C	0.83007231	0.46536424	0.50051442
C	0.16649897	0.53374211	0.49983933
C	0.63483452	0.17004625	0.50054490
C	0.36769317	0.83337242	0.49974601
C	0.52357222	0.55419233	0.47486753
C	0.47827435	0.44374187	0.52508833
C	0.44586114	0.96921894	0.47482149
C	0.55625058	0.03477530	0.52506771
C	0.03046034	0.47628006	0.47491903
C	0.96555115	0.52182507	0.52508483
C	0.44580131	0.47643093	0.52514606

C	0.55623764	0.52169631	0.47491437
C	0.03078816	0.55414054	0.52518380
C	0.96521935	0.44376605	0.47492799
C	0.52372575	0.96953772	0.52508112
C	0.47819004	0.03445502	0.47491857
C	0.63368668	0.36630209	0.49999897
C	0.36680287	0.63320419	0.49999954
C	0.63362350	0.26768712	0.50001177
C	0.36689062	0.73333142	0.49997411
C	0.73231149	0.36638252	0.49999100
C	0.26666970	0.63310915	0.50001716
C	0.56856392	0.43140311	0.49999822
C	0.43331574	0.56669815	0.50000444
C	0.56841834	0.13734367	0.50002539
C	0.43351577	0.86651269	0.49993176
C	0.86265571	0.43160238	0.49997770
C	0.13349275	0.56647866	0.50006962
C	0.46782487	0.53218294	0.50000804
C	0.53414021	0.46582521	0.49999958
C	0.46800318	0.93561764	0.49992891
C	0.53403271	0.06842272	0.50001766
C	0.06438657	0.53199679	0.50007460
C	0.93157830	0.46598708	0.49998070
C	0.66653821	0.33345902	0.49999930
C	0.33345333	0.66654925	0.49999644
C	0.26684126	0.53350940	0.50000301
C	0.46649536	0.73316828	0.49999256
C	0.26702843	0.73296866	0.49999087
O	0.73247281	0.46518612	0.49998524
O	0.53480998	0.26750745	0.50001437
O	0.73234050	0.26767312	0.50000160
H	0.54029214	0.59638138	0.45402509
H	0.46143389	0.40154332	0.54593447
H	0.40369580	0.94358817	0.45396447
H	0.59843915	0.06029235	0.54591564
H	0.05583801	0.45947560	0.45409919
H	0.94028249	0.53874704	0.54590986
H	0.40362166	0.45973044	0.54599777
H	0.59843153	0.53852089	0.45406358
H	0.05642589	0.59630283	0.54604338
H	0.93969851	0.40158515	0.45407520
H	0.54052662	0.94415325	0.54589712
H	0.46127238	0.05972907	0.45409709
H	0.66081673	0.51645678	0.50017404

H 0.34180081 0.48256246 0.49921802
H 0.48340889 0.14443471 0.50022446
H 0.51760595 0.85922127 0.49920821
H 0.85536144 0.33940834 0.50020608
H 0.14100369 0.65796448 0.49926667
H 0.48351765 0.33914181 0.49982072
H 0.51745216 0.65821318 0.50078466
H 0.85555333 0.51660428 0.49978192
H 0.14078590 0.48239295 0.50079097
H 0.66062147 0.14465368 0.49979880
H 0.34202564 0.85899125 0.50072912
H 0.51791490 0.75889005 0.49999456
H 0.24134526 0.75864517 0.49998706
H 0.24112276 0.48208955 0.49999804

[TRI-Ph-TAM]
a = 24.57, 24.57, c = 29.40, alpha = 90.0, beta = 90.0, gamma = 120.1
C 0.66711130 0.43241325 0.49965737
C 0.33306715 0.56588309 0.50016634
C 0.56750879 0.23491838 0.49968495
C 0.43421072 0.76702381 0.50014493
C 0.76497911 0.33301989 0.49966114
C 0.23309581 0.66675720 0.50019752
C 0.56757240 0.33286784 0.50034031
C 0.43412344 0.66694166 0.49983094
C 0.76507195 0.43249601 0.50031738
C 0.23298259 0.56579154 0.49985005
C 0.66699328 0.23502591 0.50034165
C 0.33323793 0.76690194 0.49979039
C 0.63499187 0.46521620 0.49945165
C 0.36749112 0.53390886 0.50018974
C 0.53464891 0.16991953 0.49949116
C 0.46625615 0.83350767 0.50015793
C 0.82996139 0.36518754 0.49945801
C 0.16662409 0.63229834 0.50024641
C 0.53475936 0.36497585 0.50054425
C 0.46610408 0.63252195 0.49981332
C 0.83007231 0.46536424 0.50051442
C 0.16649897 0.53374211 0.49983933
C 0.63483452 0.17004625 0.50054490
C 0.36769317 0.83337242 0.49974601
C 0.52357222 0.55419233 0.47486753
C 0.47827435 0.44374187 0.52508833
C 0.44586114 0.96921894 0.47482149

C	0.55625058	0.03477530	0.52506771
C	0.03046034	0.47628006	0.47491903
C	0.96555115	0.52182507	0.52508483
C	0.44580131	0.47643093	0.52514606
C	0.55623764	0.52169631	0.47491437
C	0.03078816	0.55414054	0.52518380
C	0.96521935	0.44376605	0.47492799
C	0.52372575	0.96953772	0.52508112
C	0.47819004	0.03445502	0.47491857
C	0.63368668	0.36630209	0.49999897
C	0.36680287	0.63320419	0.49999954
C	0.63362350	0.26768712	0.50001177
C	0.36689062	0.73333142	0.49997411
C	0.73231149	0.36638252	0.49999100
C	0.26666970	0.63310915	0.50001716
C	0.56856392	0.43140311	0.49999822
C	0.43331574	0.56669815	0.50000444
C	0.56841834	0.13734367	0.50002539
C	0.43351577	0.86651269	0.49993176
C	0.86265571	0.43160238	0.49997770
C	0.13349275	0.56647866	0.50006962
C	0.46782487	0.53218294	0.50000804
C	0.53414021	0.46582521	0.49999958
C	0.46800318	0.93561764	0.49992891
C	0.53403271	0.06842272	0.50001766
C	0.06438657	0.53199679	0.50007460
C	0.93157830	0.46598708	0.49998070
C	0.66653821	0.33345902	0.49999930
C	0.33345333	0.66654925	0.49999644
C	0.26684126	0.53350940	0.50000301
C	0.46649536	0.73316828	0.49999256
C	0.26702843	0.73296866	0.49999087
O	0.73247281	0.46518612	0.49998524
O	0.53480998	0.26750745	0.50001437
O	0.73234050	0.26767312	0.50000160
H	0.54029214	0.59638138	0.45402509
H	0.46143389	0.40154332	0.54593447
H	0.40369580	0.94358817	0.45396447
H	0.59843915	0.06029235	0.54591564
H	0.05583801	0.45947560	0.45409919
H	0.94028249	0.53874704	0.54590986
H	0.40362166	0.45973044	0.54599777
H	0.59843153	0.53852089	0.45406358
H	0.05642589	0.59630283	0.54604338

H 0.93969851 0.40158515 0.45407520
H 0.54052662 0.94415325 0.54589712
H 0.46127238 0.05972907 0.45409709
H 0.66081673 0.51645678 0.50017404
H 0.34180081 0.48256246 0.49921802
H 0.48340889 0.14443471 0.50022446
H 0.51760595 0.85922127 0.49920821
H 0.85536144 0.33940834 0.50020608
H 0.14100369 0.65796448 0.49926667
H 0.48351765 0.33914181 0.49982072
H 0.51745216 0.65821318 0.50078466
H 0.85555333 0.51660428 0.49978192
H 0.14078590 0.48239295 0.50079097
H 0.66062147 0.14465368 0.49979880
H 0.34202564 0.85899125 0.50072912
H 0.51791490 0.75889005 0.49999456
H 0.24134526 0.75864517 0.49998706
H 0.24112276 0.48208955 0.49999804

[TRI-Ph-TRIH]
a = 24.77, 24.77, c = 29.40, alpha = 90.0, beta = 90.0, gamma = 120.1
C 0.66528398 0.43322193 0.49954878
C 0.33313532 0.56669293 0.50015769
C 0.56677414 0.23214227 0.49986745
C 0.43341047 0.76622923 0.50010303
C 0.76785152 0.33487416 0.49986697
C 0.23390460 0.66679769 0.50016103
C 0.56682885 0.33476747 0.50062210
C 0.43334552 0.66689805 0.49989787
C 0.76792344 0.43328821 0.50025904
C 0.23380871 0.56665003 0.49987356
C 0.66518408 0.23222153 0.50060624
C 0.33327408 0.76615827 0.49981850
C 0.63228391 0.46486212 0.49921931
C 0.36730674 0.53496562 0.50009620
C 0.53508484 0.16744664 0.49944907
C 0.46520171 0.83218661 0.49998351
C 0.83253790 0.36790555 0.49952410
C 0.16795708 0.63260689 0.50012102
C 0.53518469 0.36776064 0.50084642
C 0.46508120 0.63273418 0.49993090
C 0.83262564 0.46497876 0.50046083
C 0.16785101 0.53487896 0.49989533
C 0.63214788 0.16753579 0.50074747

C	0.36748263	0.83210371	0.49975983
C	0.52234351	0.55513929	0.47499989
C	0.47720369	0.44532723	0.52492861
C	0.44496747	0.96701576	0.47483436
C	0.55473130	0.03193275	0.52479655
C	0.03283396	0.47758460	0.47506865
C	0.96832737	0.52300545	0.52482413
C	0.44490505	0.47770267	0.52494627
C	0.55471016	0.52282717	0.47501335
C	0.03305635	0.55519931	0.52488205
C	0.96810445	0.44529861	0.47506361
C	0.52246904	0.96720739	0.52475077
C	0.47714517	0.03174847	0.47493098
C	0.63307723	0.36698039	0.50013667
C	0.36657106	0.63346720	0.50003610
C	0.63302626	0.26625120	0.50025815
C	0.36663904	0.73281428	0.49998876
C	0.73382211	0.36703646	0.50012195
C	0.26723662	0.63342123	0.50002512
C	0.56688492	0.43315772	0.50001208
C	0.43262141	0.56742422	0.49999886
C	0.56675590	0.13376623	0.50001492
C	0.43279147	0.86502957	0.49981871
C	0.86629910	0.43330366	0.49996566
C	0.13501770	0.56730051	0.50000419
C	0.46689146	0.53315741	0.49997671
C	0.53274168	0.46729611	0.49997550
C	0.46703288	0.93361875	0.49976711
C	0.53265963	0.06542719	0.49990551
C	0.06643112	0.53307028	0.49999268
C	0.93463830	0.46741973	0.49993471
C	0.66664276	0.33342540	0.50021372
C	0.33348157	0.66656769	0.50003029
C	0.73524452	0.47051127	0.49966722
C	0.26742422	0.53461641	0.50000377
C	0.52954508	0.26481076	0.50044013
C	0.46542871	0.73260283	0.50001804
C	0.73513266	0.26493166	0.50045102
C	0.26758001	0.73249128	0.50000522
H	0.53909147	0.59714179	0.45430298
H	0.46041281	0.40334415	0.54564904
H	0.40298108	0.94166874	0.45412288
H	0.59668420	0.05722742	0.54553911
H	0.05802420	0.46074788	0.45442075

H 0.94316634 0.53987868 0.54546903
H 0.40290478 0.46096214 0.54564747
H 0.59669715 0.53961183 0.45429404
H 0.05841026 0.59721918 0.54555698
H 0.94278142 0.40326745 0.45439908
H 0.53927230 0.94201275 0.54542869
H 0.46028417 0.05690957 0.45427491
H 0.65811363 0.51586463 0.49958616
H 0.34179667 0.48404350 0.49902880
H 0.48408187 0.14220369 0.49994869
H 0.51612591 0.85763515 0.49892058
H 0.85772369 0.34213403 0.50017803
H 0.14257330 0.65810990 0.49906443
H 0.48417960 0.34193046 0.50040599
H 0.51600887 0.65824700 0.50097941
H 0.85787571 0.51597664 0.49974105
H 0.14237658 0.48395596 0.50092694
H 0.65794953 0.14238116 0.50016177
H 0.34199608 0.85750074 0.50077982
H 0.51642660 0.75811743 0.50003063
H 0.49768571 0.24920788 0.47085399
H 0.24210714 0.75797611 0.50000771
H 0.75074911 0.24867260 0.47088847
H 0.24189440 0.48361341 0.49998406
H 0.75090370 0.50244217 0.52921307
H 0.75113948 0.50167254 0.46968640
H 0.49830867 0.24885270 0.53038047
H 0.75102859 0.24966528 0.53042000

[TRI-Ph-TPM]
a = 24.82, 24.83, c = 29.40, alpha = 90.0, beta = 90.0, gamma = 120.2
H 0.55278255 0.60975115 0.47324133
H 0.44691843 0.38976946 0.52521331
H 0.39065211 0.94232983 0.47315578
H 0.61078074 0.05821008 0.52487134
H 0.05650582 0.44681713 0.47309545
H 0.94306809 0.55298490 0.52481053
H 0.39037939 0.44764824 0.52699076
H 0.61050138 0.55305636 0.47521130
H 0.05774060 0.60938968 0.52656393
H 0.94178786 0.38923502 0.47509859
H 0.55317104 0.94353352 0.52679890
H 0.44706887 0.05697027 0.47499707
H 0.64538918 0.50393032 0.53786833

H	0.34148255	0.48466774	0.49601340
H	0.49613626	0.14126928	0.53775905
H	0.51591473	0.85683895	0.49673536
H	0.85772841	0.35472212	0.53787765
H	0.14374136	0.65816091	0.49600866
H	0.49597815	0.35470128	0.46267039
H	0.51564032	0.65883193	0.50397165
H	0.85849399	0.50375410	0.46229354
H	0.14332991	0.48419714	0.50307446
H	0.64532890	0.14244381	0.46205013
H	0.34179716	0.85625646	0.50354499
H	0.51637729	0.75812420	0.50034509
H	0.24250440	0.75747888	0.49969618
H	0.24213556	0.48383692	0.49946222
H	0.55399071	0.25627788	0.53910646
H	0.74271150	0.29815867	0.53927197
H	0.55394272	0.29794626	0.46139979
H	0.74344644	0.44585749	0.46122329
H	0.70182528	0.25742651	0.46089676
H	0.70203700	0.44585536	0.53912256
C	0.65756162	0.42638052	0.52132383
C	0.33331024	0.56691338	0.49931618
C	0.57348130	0.23150072	0.52116729
C	0.43346326	0.76610850	0.49975975
C	0.76790811	0.34253544	0.52136878
C	0.23425559	0.66646707	0.49924016
C	0.57345554	0.34237376	0.47924409
C	0.43332731	0.66693504	0.50045541
C	0.76832217	0.42641229	0.47908632
C	0.23405061	0.56666945	0.49994637
C	0.65744655	0.23214998	0.47879985
C	0.33358248	0.76581276	0.50029789
C	0.62571298	0.45887557	0.52078457
C	0.36746818	0.53538798	0.49848931
C	0.54113024	0.16699816	0.52058917
C	0.46515786	0.83196392	0.49894319
C	0.83241701	0.37433175	0.52073804
C	0.16840621	0.63221510	0.49831707
C	0.54103625	0.37429936	0.47977165
C	0.46490900	0.63283139	0.50142479
C	0.83285342	0.45879438	0.47950635
C	0.16817077	0.53493215	0.50079705
C	0.62568943	0.16766452	0.47928594
C	0.36779461	0.83164204	0.50126892

C	0.52957359	0.56203173	0.48599384
C	0.46998309	0.43811038	0.51367092
C	0.43840843	0.96719595	0.48584820
C	0.56240478	0.03254076	0.51337301
C	0.03215814	0.47012027	0.48576162
C	0.96818950	0.52985226	0.51330537
C	0.43813859	0.47076189	0.51429148
C	0.56213578	0.53010058	0.48673145
C	0.03285657	0.56162915	0.51390926
C	0.96748116	0.43762182	0.48652847
C	0.52989213	0.96788471	0.51409673
C	0.47018594	0.03183789	0.48650046
C	0.63259821	0.36726510	0.50027409
C	0.36667431	0.63354105	0.49985858
C	0.63253252	0.26609280	0.50004755
C	0.36680958	0.73258538	0.49996558
C	0.73383558	0.36739824	0.50025271
C	0.26753829	0.63330068	0.49966220
C	0.56671125	0.43330038	0.50026939
C	0.43277229	0.56754795	0.50000937
C	0.56680025	0.13388801	0.49994194
C	0.43310234	0.86504948	0.50008480
C	0.86609545	0.43319088	0.50004736
C	0.13502964	0.56693278	0.49961571
C	0.46702843	0.53327052	0.50011345
C	0.53269867	0.46741965	0.50022634
C	0.46734150	0.93375129	0.50001152
C	0.53289353	0.06559429	0.49993370
C	0.06630529	0.53268491	0.49976278
C	0.93441548	0.46713315	0.49993842
C	0.66628522	0.33361094	0.50021419
C	0.33367282	0.66647568	0.49982005
C	0.26763454	0.53476819	0.49954458
C	0.46543078	0.73258846	0.50020851
C	0.26793354	0.73209572	0.49973420
